# Bioinformatics and *In Vitro* Studies Reveal the Importance of p53, PPARG and Notch Signaling Pathway in Inhibition of Breast Cancer Stem Cells by Hesperetin

**DOI:** 10.34172/apb.2021.033

**Published:** 2020-04-19

**Authors:** Adam Hermawan, Muthi Ikawati, Annisa Khumaira, Herwandhani Putri, Riris Istighfari Jenie, Sonia Meta Angraini, Haruma Anggraini Muflikhasari

**Affiliations:** ^1^Laboratory of Macromolecular Engineering, Department of Pharmaceutical Chemistry, Faculty of Pharmacy, Universitas Gadjah Mada, Sekip Utara II, 55281 Yogyakarta, Indonesia.; ^2^Cancer Chemoprevention Research Center, Faculty of Pharmacy, Universitas Gadjah Mada, Sekip Utara II, 55281 Yogyakarta, Indonesia.

**Keywords:** Hesperetin, Breast cancer stem cells, Bioinformatics, In vitro, Mammosphere, Targeted therapy

## Abstract

***Purpose:*** The failure of chemotherapy in breast cancer is caused by breast cancer stem cells (BCSCs), a minor population of cells in bulk mammary tumors. Previously, hesperetin, a citrus flavonoid, showed cytotoxicity in several cancer cells and increased cytotoxicity of doxorubicin and cisplatin. Hesperetin also inhibited osteogenic and adipocyte differentiation, however, a study of the effect of hesperetin on BCSCs has not yet been performed.

***Methods:*** In this study, we combined bioinformatics and *in vitro* works. A bioinformatic approach was performed to identify molecular targets, key proteins, and molecular mechanisms of hesperetin targeted at BCSCs, and genetic alterations among key genes. In addition, an *in vitro* study was carried out to measure the effects of hesperetin on BCSCs using the spheroids model of MCF-7 breast cancer cells (mammospheres).

***Results:*** Using a bioinformatics approach, we identified P53, PPARG, and Notch signaling as potential targets of hesperetin in inhibition of BCSCs. The *in vitro* study showed that hesperetin exhibits cytotoxicity on mammospheres, inhibits mammosphere and colony formation, and inhibits migration. Hesperetin modulates the cell cycle and induces apoptosis in mammospheres. Moreover, hesperetin treatment modulates the expression of *p53*, *PPARG*, and *NOTCH1*.

***Conclusion:*** Taken together, hesperetin has potential for the treatment of BCSC by targeting p53, PPARG and Notch signaling. Further investigation of the molecular mechanisms involved is required for the development of hesperetin as a BCSC-targeted drug.

## Introduction

Drug discovery in the era of information technology has become easier, faster and directed to molecularly targets with the aid of artificial intelligence, cheminformatics, and data mining, as well as high throughput screening.^1^ One application is the use of an integrated bioinformatics approach to obtain molecular targets, identification of key proteins, and molecular mechanisms of a drug candidate.^2,3^ Hence, drug development for certain diseases, such as cancer, can be performed in a faster and more strategic way using integrated bioinformatics analysis.

Breast cancer is the leading cause of death among women worldwide.^4^ The failure of chemotherapy in breast cancer is caused by breast cancer stem cells (BCSCs), a minor population of cells in bulk mammary tumors.^5^ BCSCs are considered to possess stem-cell characteristics, which are self-renewal and differentiation, and thus are responsible for tumor relapse and metastasis.^6^ Targeted BCSC therapy has proven to be effective as a companion to chemotherapy in breast cancer, namely combination therapy.^7^ Candidate compounds for combination therapy derive mostly from natural ingredients that exhibit potent cytotoxicity toward cancer cells but low toxicity to normal cells.^8^

**Figure 1:**
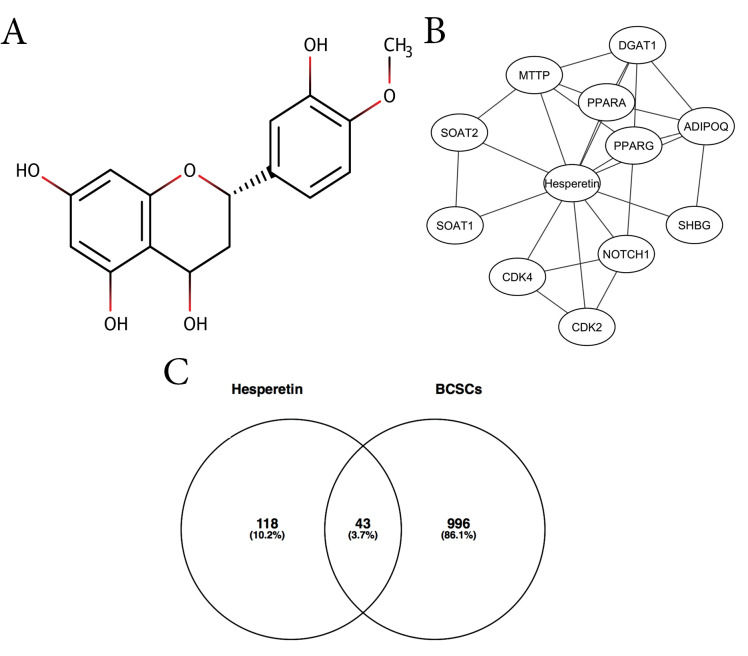
**(A)** The structure of hesperetin. **(B)** Interactions of hesperetin and its DTPs. **(C)** A Venn diagram of hesperetin potential targets (HTs) against BCSCs.

One natural compound that can be developed for combination with chemotherapy is hesperetin (Figure 1A), a flavonoid that is found in many citrus species. Previous studies have shown that hesperetin exhibited cytotoxicity by inducing apoptosis and modulating the cell-cycle in various types of cancer cells, such as breast cancer cells,^9^ cervical, colon, prostate cancer cells,^10^ leukemia cells,^11^ gastric cancer cells,^12^ esophageal cancer cells,^13^ skin carcinoma cells,^14^ and hepatocellular carcinoma cells.^15^ Moreover, hesperetin exhibited anticancer activity in animal cancer models, such as rat colon cancer,^16^ DMBA-induced rat mammary tumor,^17^ DMBA-induced hamster buccal pouch carcinogenesis,^18^ and benzo(a)pyrene-induced lung carcinogenesis in Swiss albino mice.^19^

In addition, hesperetin has also been shown to increase the effectiveness of chemotherapy such as that mediated by doxorubicin and cisplatin, on various cancer cells. Hesperetin increased cytotoxicity of doxorubicin on MCF-7 breast cancer cells.^20^ Furthermore, hesperetin showed synergism to platinum-based chemotherapy by inhibiting UGT1A3 and increasing levels of reactive oxygen species (ROS) in lung adenocarcinoma cells.^21^ Hesperetin also has an effect on stem cells, which inhibit osteogenic differentiationand adipocyte differentiation.^22,23^ However, a study on hesperetin-targeted BCSCs has not yet been conducted.

In this study, we combined bioinformatics and *in vitro* work. A bioinformatic approach was performed to identify molecular targets, key proteins, and molecular mechanisms of hesperetin targeted at BCSCs. In addition, an *in vitro* study was carried out to measure the effects of hesperetin on BCSCs using the spheroid model of MCF-7 breast cancer cells (mammospheres). This study is expected to be the basis for the development of hesperetin as a BCSC-targeted drug for overcoming chemotherapy resistance in breast cancer therapy. We identified possible specific molecular targets of hesperetin in BCSC inhibition. This analysis suggested that p53, PPARG, and Notch signaling could be developed as targets of hesperetin for targeting BCSCs.

## Materials and Methods

### Acquisition of direct target proteins, indirect protein targets, and BCSC regulatory genes

Direct target proteins (DTPs) of hesperetin were searched from STITCH (http://stitch.embl.de).^24^ The indirect protein targets (ITPs) of each DTP were obtained from the STRING database (https://string-db.org),^25^ with a minimum interaction score of 0.7 and the maximum number of interactors as 20. The ITPs of all DTPs were generated after removing repetitive proteins. BCSC regulatory genes were retrieved from PubMed with the keywords “breast cancer stem cells”. A Venn diagram between all ITPs, DTPs and BCSC regulatory genes was constructed using Venny 2.1 (http://bioinfogp.cnb.csic.es/tools/venny/).^26^ The overlapping genes were considered as hesperetin targets (HTs) in BCSCs.

### Protein-protein interaction (PPI) network, gene ontology and KEGG-pathway enrichment of the HT

PPI network analysis among HTs was conducted with STRING-DB v11.0 with confidence scores >0.7 and visualized by Cytoscape software.^25,27^ Genes with a degree score of more than 10, analyzed using CytoHubba plugin, were selected as hub proteins.^28^ Analysis of gene ontology (GO) and Kyoto Encyclopedia of Genes and Genomes (KEGG) pathway enrichment were conducted using The Database for Annotation, Visualization and Integrated Discovery (DAVID) v6.8 with P < 00.05 selected as the cut-off value.^29^

### Analysis of genetic alterations among hub proteins

The genetic alterations of selected genes were analyzed using cBioPortal (http://www.cbioportal.org).^30,31^ Protein genes including *TP53, PPARG, PCNA, HES1*, and *MYC* were screened for genetic alterations in all breast cancer studies available in the cBioportal database. The breast cancer study with the highest number of genetic alterations was chosen for Oncoprint and connectivity analysis.

### Cell culture and mammosphere generation

MCF-7 cells were cultured using DMEM high glucose media containing 10% fetal bovine serum (FBS) and penicillin-streptomycin, incubated in CO_2_ incubators at 37°C. The formation of mammospheres was carried out with modifications from previous studies.^32-34^ Briefly, MCF-7 cells were seeded (40 000 cells/mL) on a 50 mg/mL poly-HEMA-coated plate. Mammospheres were grown in DMEM media containing 10 ng/mL epidermal growth factor (EGF), 10 ng/mL basic fibroblast growth factor (bFGF), 5 µg/mL insulin, and penicillin-streptomycin, and incubated in a CO_2_ incubator at 37°C. Mammospheres were allowed to grow for a maximum of 7 days before being tested with hesperetin (HST, Sigma-Aldrich) and other compounds (doxorubicin or DXR, Sigma-Aldrich; metformin or MET, Sigma-Aldrich).

### Cytotoxicity test

Cytotoxicity tests were carried out using MTT methods.^35^ Briefly, cells were seeded in a 96-well plate to form monolayer cells and mammospheres. Cells were then treated with hesperetin and incubated for 72 hours. At the end of incubation, the MTT solution was added followed by incubation for 3 hours. After that, a 10% SDS in 0.01 M HCl solution was added until formazan crystal completely dissolved. Absorbance was then read with a microplate reader at a wavelength of 570 nm.

### Mammosphere-forming potential

The effect of hesperetin in mammosphere forming was examined based on a previous study.^33^ Briefly, cells were pre-treated with hesperetin for 72 hours. The medium was changed with fresh medium and cells were then incubated for the following 24 hours. Recovered cells were then seeded (40  000 cells/well) in poly-HEMA-coated 96-well plates and incubated for 96 hours. At the end of the observation, the number of mammospheres formed was calculated manually. Results are expressed as mammosphere-forming potential (MFP).

### Colony formation assay

Cells were seeded (1000 cells/well) in a 6-well plate and incubated for 24 hours. Cells were then treated with drugs for 72 hours. At the end of treatment, the medium was changed with a fresh medium and followed by 14 days of incubation. At the end of the incubation, the cells were fixed with paraformaldehyde and stained with a Gentian violet solution. The surviving colonies were counted and analyzed with ColonyArea.^36^

### Wound-healing assay

Mammosphere-derived MCF-7 cells were seeded (10 000 cells/well) into 24-well plates and incubated for 24 hours. Starvation was carried out by replacing culture media with starvation media containing only 0.5% of FBS and incubated for the following 24 hours. Scratching was performed by using a yellow tip on each well. Cells were washed once with PBS and 1 mL of media containing drugs was added. Observation and documentation of cell conditions were carried out at intervals of 0, 18, 24, and 48 hours using an inverted microscope. Images were analyzed using ImageJ and calculated as percent closure after 48 hours of treatment.

### Analysis of cell cycle

Cell cycle observations were carried out as in a previous study with modification.^37^ Briefly, cells were seeded, incubated, and treated with hesperetin for 72 hours. At the end of treatment, cells were harvested with trypsin to obtain a single-cell suspension. Cells were then fixed with cold methanol, and stained for 20 minutes in dark conditions using a solution containing 100 mg/mL of propidium iodide (PI), 50 mg/mL RNAse, and triton X. Cells were then analyzed using the BD FACSCalibur flow cytometry system. Cell cycle profile was presented as percent of cell population in G0/G1, S, and G2/M phase.

### Apoptosis assay

Cells were seeded, incubated, and treated with hesperetin for 96 hours. At the end of treatment, cells were harvested with trypsin to obtain a single-cell suspension. Apoptotic observations were carried out using the Annexin-V-FLUOS staining kit according to the manufacturer’s instructions. Briefly, cell suspensions were prepared by trypsinization, and Annexin-V-FLUOS staining kit containing binding buffer, Annexin and PI was added and the mixture incubated in the dark for 10 minutes. Cells were then examined using the BD FACSCalibur flow cytometry system to measure the percentage of cells undergoing apoptosis.

### q-RT PCR

Cells were seeded, incubated, and treated with hesperetin for 96 hours. Total mRNA was isolated using GeneJet RNA Purification Kit (Thermo Fisher Scientific), according to the manufacturer’s instructions. Next, cDNA was synthesized using RevertAid First Strand cDNA Synthesis Kit (Thermo Fisher Scientific). SsoFast EvaGreen Supermix (Bio-Rad) was used to quantify PCR. Expression of regulatory genes was conducted using selected primers (Supplementary file 1 , Table S1). GAPDH was used as a housekeeping gene. The results were analyzed using the comparative threshold cycle (ΔΔCT method).

### Statistical analysis

All statistical analyses were conducted with GraphPad Prism 5.0 software.

## Results and Discussion

### Acquisition of DTPs, ITPs and BCSC regulatory genes

This study aimed to explore the molecular target of hesperetin in the inhibition of BCSCs using integrated bioinformatics and *in vitro* studies. We obtained 11 DTPs of hesperetin, including PPARA, PPARG, ADIPOQ, DGAT1, MMTP, SOAT2, SOAT1, CDK4, CDK2, NOTCH1, and SHBG (Figure 1B, Table 1). We showed interactions among the DTPs, including CDK4-NOTCH1-CDK2, PPARG-ADIPOQ, DGAT1-ADIPOQ, MTTP-ADIPOQ, and PPARA-DGAT1. These interactions indicated that proteins played a critical role in the molecular function mediated by hesperetin. In total, we retrieved hesperetin mediated proteins consisting of 11 DTPs and 98 ITPs (Table S2), and 1041 BCSC regulatory genes from PubMed (Table S3). A Venn diagram generated 43 hesperetin targets in BCSCs or HT (Figure 1C, Table S4).

**Table 1:** Direct protein targets of hesperetin, from DrugBank and STITCH

**Gene symbol**	**Protein name**
*SOAT1*	Sterol O-acyltransferase 1
*SOAT2*	Sterol O-acyltransferase 2;
*MTTP*	Microsomal triglyceride transfer protein large subunit
*SHBG*	Sex hormone-binding globulin
*CDK2*	Cyclin-dependent kinase 2
*NOTCH1*	Neurogenic locus notch homolog protein 1
*CDK4*	Cyclin-dependent kinase 4
*PPARG*	Peroxisome proliferator-activated receptor gamma
*ADIPOQ*	Adiponectin
*PPARA*	Peroxisome proliferator-activated receptor alpha
*DGAT1*	Diacylglycerol O-acyltransferase 1

### Protein-protein interaction network analysis, gene ontology, and KEGG-pathway enrichment of the HT

**Figure 2:**
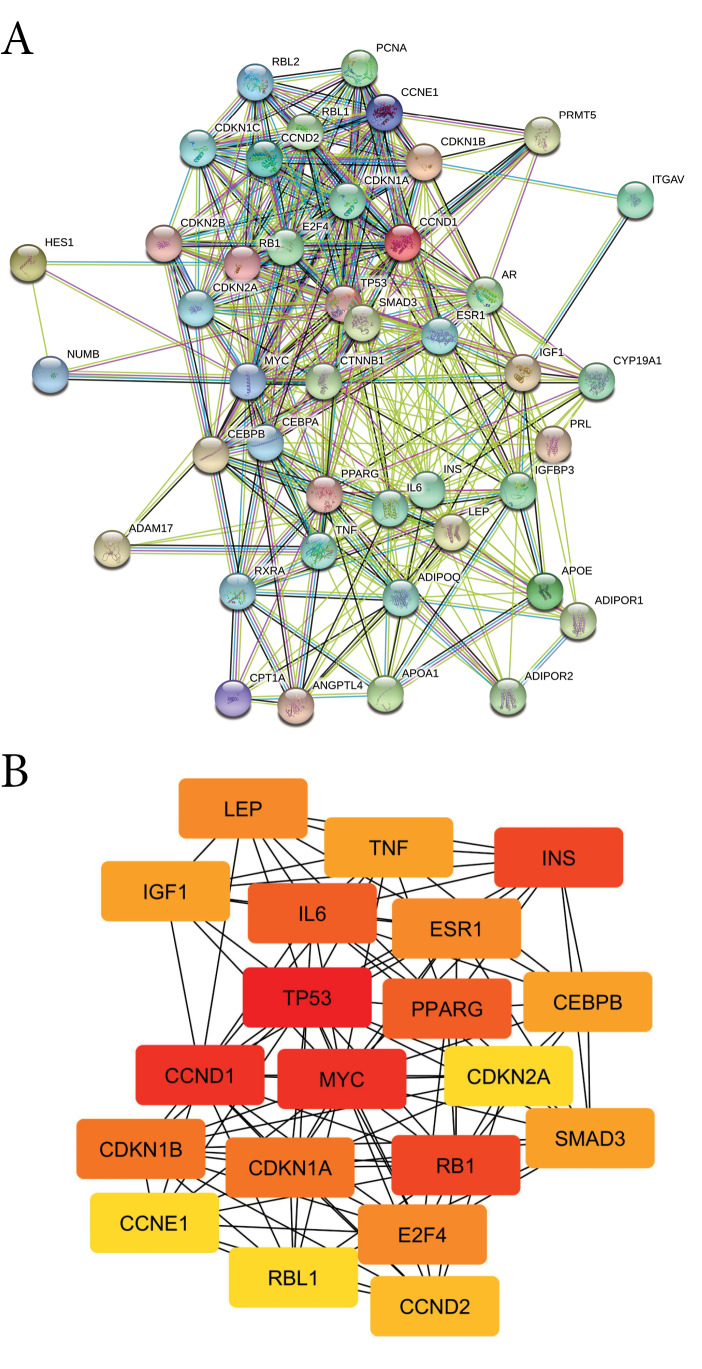
** (A)** PPI network of hesperetin protein targets. **(B)** Top 20 hub proteins based on degree score, analyzed by CytoScape.

The PPI network of HT consists of 43 nodes, 234 edges, an average node degree of 17, and a high-confidence interaction (0.7; Figure 2A). Furthermore, hub proteins were selected from the PPI network based on their degree score (Table 2) including one DTP (PPARG). These results indicated that the biological effect of hesperetin is strongly correlated with PPARG. GO analysis of HT was classified into three groups, consisting of biological process, cellular component and molecular function (Table S5). HT was found to participate in the biological processes of cell cycle and cell proliferation. The HT is located in the nucleoplasm, cytosol extracellular matrix, and cytoplasm. Moreover, the HT has a molecular function in cyclin-dependent protein kinase regulator and inhibitor activity. KEGG-pathway enrichment analysis showed 21 pathways regulated by HT (Table S6), including the cell cycle, and TGF-beta, PPAR, Wnt, and Notch signaling pathways.

**Table 2:** The top 20 hub protein based on highest degree score

**Gene symbol**	**Degree score**
*TP53*	25
*CCND1*	22
*MYC*	22
*RB1*	19
*INS*	19
*IL6*	17
*PPARG*	17
*CDKN1A*	16
*CDKN1B*	16
*E2F4*	15
*LEP*	15
*ESR1*	15
*CEBPB*	14
*TNF*	14
*IGF1*	14
*SMAD3*	14
*CCND2*	13
*RBL1*	12
*CCNE1*	12
*CDKN2A*	12

### Genetic alterations among the hub proteins

**Figure 3:**
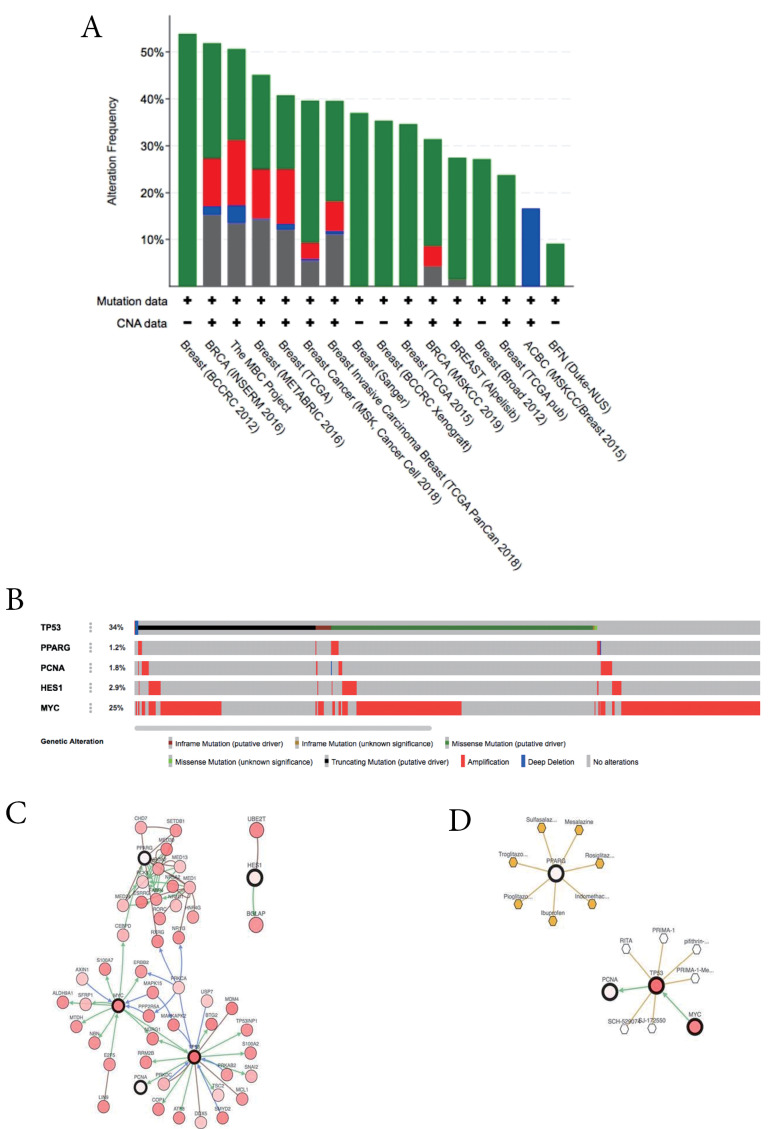
**(A)** Overview of changes in *TP53, PPARG, PCNA, HES1,* and * MYC* in genomics dataset from 12 studies of breast cancer. **(B)** Summary alterations of *TP53, PPARG, PCNA, HES1* and * MYC* across breast cancer samples (based on a study by Pereira et al^32^).

A total of five HT were analyzed using cBioportal to explore their genomic alterations across breast cancer studies. HT consists of *TP53, PPARG, MYC* (based on highest score degree), *TP53, PCNA* (based on KEGG pathway enrichment results in the cell cycle), *PPARG* (based on KEGG pathway enrichment results in PPAR signaling pathway), and *HES1* (based on KEGG pathway enrichment analysis in Notch signaling pathway). Among twelve breast cancer studies, a study namely METABRIC by Pereira et al was selected for further analysis.^38^ Oncoprint analysis showed that genetic alterations of HT occur in 1.2 to 34% of patients samples (Figure 3B), in which amplification is the most common gene alteration. Additional analysis of the interactive relationship between five selected genes and altered genes in the METABRIC study revealed a network contains five queries and neighbor genes (Figure 3C). In addition, *TP53* and *MYC* were the genes with the highest number of neighbor genes. Moreover, *TP53* and *MYC* were the main targets of most cancer drugs (Figure 3D), thus indicating the potential of p53 and Myc to be hesperetin targets in BCSCs.

### Mammosphere generation and characterization

**Figure 4:**
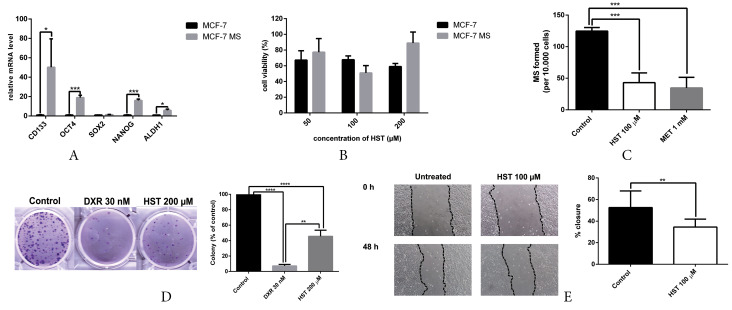
**(A)** Upregulation of BCSC markers in mammospheres from MCF-7 cells. Gene expression was measured by q-RT PCR. Results are shown as relative to monolayer cells and are expressed as mean±SD (n=3). **(B)** Hesperetin showed cytotoxicity on MCF-7 monolayers and mammospheres. Cytotoxicity was measured by MTT and shown as % cell viability compared to control (mean±SD; n=3). **(C)** Hesperetin inhibits mammosphere formation from MCF-7 cells. Results are expressed as mammospheres-forming potential (MFP) as mean±SD (n=3). **(D)** Hesperetin inhibits colony formation. The surviving colonies were counted and analyzed with ColonyArea, and the results are shown as mean±SD (n=3). **(E)** Hesperetin inhibits the migration of mammosphere-derived MCF-7 cells. Observation and documentation of cells were carried out from 0 to 48 hours. Results are expressed as mean±SD (n=6). Statistical analyses were conducted using Student's *t* test. ***P < 00.001, **** *P* <0.0001.

We successfully generated mammospheres to enrich BCSC properties in MCF-7 cells. Cells were cultured in poly-HEMA-coated plates using serum-free medium supplemented with insulin, EGF, and B27, as described in the methods section. BCSC characterization using q-RT PCR showed upregulation of BCSC markers, including *CD133*, *OCT4*, *NANOG* and *ALDH1* (Figure 4A). Taken together, the mammospheres were enriched with BCSC properties and could be used for further analysis.

### Hesperetin exhibits cytotoxicity, inhibits mammosphere and colony formation and hampers migration

Cytotoxicity assay with MTT showed that hesperetin exhibited cytotoxicity in MCF-7 cell monolayers (M) and mammospheres (MS) at a concentration of 100 µM (Figure 4B). We performed cytotoxicity assay at a different time point, i.e., 24, 48 and 72 hours. However, we only showed the data of 72 hours experiment, since hesperetin did not exhibit cytotoxicity on MS in 24 and 48 hours, shown by cell viability value above 90%.

A compound for combination therapy (i.e., alongside chemotherapy) should be potent, but also less toxic toward normal cells.^39^ Previous acute and chronic toxicity studies showed that hesperidin, a glycoside form of hesperetin, is not toxic in animals.^40^ Thus, those findings support the use of hesperetin as a combination agent in BCSC therapy.

Hesperetin also inhibited mammosphere forming based on MFP (Figure 4C). The clonogenic assay revealed that hesperetin inhibited colony formation from MCF-7 cells (Figure 4D). Mammosphere forming is a standard assay to measure the frequency of tumor-initiating cells in cancer cell lines,^41^ stem cell activity, and self-renewal.^42^ The colony formation assay is a standard assay of measuring the capability of single cells to grow into a colony and is a sensitive indicator of undifferentiated CSCs.^43^ One of the hallmarks of BCSCs is promoting migration and metastasis.^44^. Hesperetin hampers migration in mammosphere-derived MCF-7 cells (Figure 4E). Collectively, these findings highlighted the potential of hesperetin as a BCSC-targeted drug.

### Hesperetin inhibits cell cycle and induces apoptosis in mammospheres

**Figure 5:**
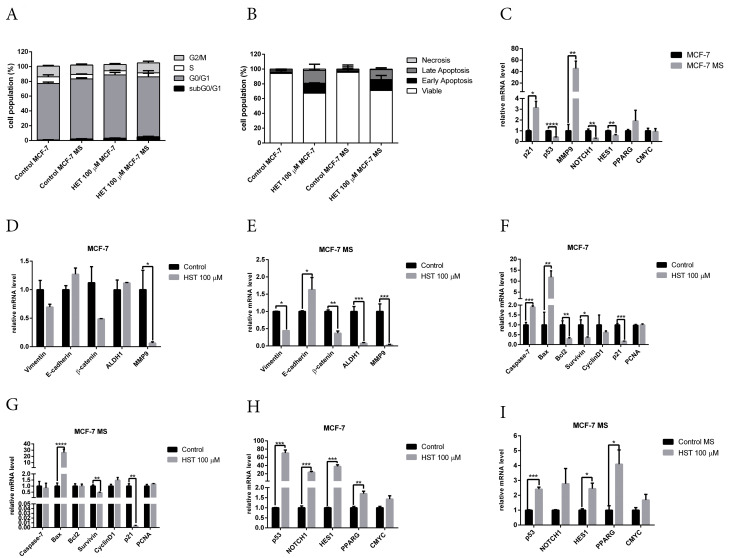
**(A)** Hesperetin modulates the cell cycle in monolayers and mammospheres. Results are shown as mean±SD (n=3). **(B)** Hesperetin induces apoptosis in monolayer and mammospheres. Results are shown as mean±SD (n=3). (C). Modulation of mRNA expression of *p21, p53, MMP9, NOTCH, HES1, PPARG* and * CMYC* in MCF-7 monolayers and mammospheres. mRNA expression of stemness regulatory genes *vimentin, E-cadherin, ß-catenin, ALDH1,* and * MMP9* upon hesperetin treatment in **(D)** MCF-7 monolayer cells and **(E)** MCF-7 mammospheres. mRNA expression of apoptosis and cell-cycle regulatory genes *caspase-7, Bax, BCL2, survivin, cyclinD, p21,* and * PCNA* upon hesperetin treatment in **(F)** MCF-7 monolayer cells and **(G)** MCF-7 mammospheres. mRNA expression of *p53, NOTCH1, HES1, PPARG,* and * CMYC* upon hesperetin treatment in **(H)** MCF-7 monolayer cells and **(I)** MCF-7 mammospheres. Gene-expression analysis was conducted using q-RT PCR with selected primers as described in the methods section. *GAPDH* was used as a housekeeping gene. Results are shown as mean±SD (n=3). Statistical analyses were conducted using Student's *t* test. ***P < 00.001, **** *P* <0.0001.

The cytotoxicity of hesperetin was further examined by measuring cell cycle and apoptosis profiles. Cell-cycle analysis results showed an increase in G0/G1 arrest in untreated mammospheres compared to monolayer MCF-7 cells (Figure 5A). In addition, hesperetin treatment increased G0/G1 arrest in monolayer cells. Moreover, hesperetin treatment showed a similar cell-cycle profile as untreated mammospheres. Apoptosis assay results revealed that hesperetin induced apoptosis in both monolayers and mammospheres (Figure 5B). In addition, the apoptosis population in monolayer cells is higher than in mammospheres.

### Effect of hesperetin in stemness properties and BCSC regulatory genes

We determined the expression of cell cycle, apoptosis, and stemness regulatory genes by q-RT PCR. To validate the results of bioinformatics analysis, we performed qRT-PCR on *p53*, *ESR1, NOTCH1, HES1, PPARG*, and *CMYC*. Gene-expression analysis with q-RT PCR revealed the downregulation of *p53, NOTCH1* and * HES1*, and the upregulation of *p21* and * MMP9* in mammospheres (Figure 5C). This result is supported by a previous study that demonstrated increased G1 arrest and p21 expression in mammospheres from MCF-7 cells.^45^

Hesperetin treatment significantly reduced *MMP9* mRNA levels in monolayer cells (Figure 5D). In mammospheres, hesperetin treatment reduced the mRNA level of *vimentin, ß-catenin, ALDH1,* and * MMP9*, and increased the mRNA level of *E-cadherin* (Figure 5E). Hesperetin treatment increased mRNA expression of apoptosis regulatory genes *caspase 7* and *BAX* in monolayer cells and reduced the mRNA level of *BCL2, survivin*, and * p21* (Figure 5F). In mammospheres, hesperetin increased the mRNA level of *BAX* and reduced the mRNA level of *p21* (Figure 5G). Hesperetin increased the mRNA level of *p53, ESR1, NOTCH1, HES1* and *PPARG* in monolayer cells (Figure 5H), and increased the mRNA level of *p53, HES1* and *PPARG* in mammospheres (Figure 5I). This result is supported by a previous study which showed that the activation of Notch signaling mediates G1/S cell cycle progression.^46^ However, hesperetin treatment did not affect the mRNA level of *CMYC* in either monolayer cells or mammospheres.

*P53* is a tumor suppressor gene and a transcription factor that regulates the cell cycle, apoptosis, and stemness.^47^ The results of this study showed the downregulation of *p53* in mammospheres (Figure 5C), which is supported by a review article which showed that *p53* is the barrier to cancer stem cell formation.^48^ Hesperetin treatment significantly increased the mRNA levels of *p53* in monolayer cells (Figure 5H) and mammospheres (Figure 5I). *p53* plays an important role in stemness properties, which is downregulated or loses function in stem cells of various cancers.^49^ A review article showed that *p53* is important in cell cycle arrest induction by hesperetin.^50^ Collectively, the future study of the role of *p53* in hesperetin treatment in BCSCs is warranted.

KEGG-pathway enrichment analysis revealed that the Notch and PPAR signaling pathways are regulated by hesperetin (Table S5). Notch1 protein, a member of the Notch family of receptors, plays a crucial role in the biological processes of cell proliferation, cell fate, differentiation, and cell death.^51^ Activation of the Notch signaling pathway plays a pivotal role in cancer development and maintenance of CSCs properties.^52,53^ Signaling by Notch is initiated upon ligand binding to the Notch receptor, followed by proteolytic cleavage by ADAM-family proteases and gamma-secretase and release of the Notch intracellular domain and translocation of Notch intracellular domain into the nucleus to bind the repressor of Notch target gene activator.^54^ Notch signaling induces transcription of target genes, including *HES1, CYMC, p21, p53*, and *CyclinD1*.^55^ Overexpression of Notch1 leads to constitutive activation of Notch signaling and apoptosis in human fibroblast cells.^56^ The results of this study showed that hesperetin increased the mRNA level of *NOTCH1, HES1*, and *p53* (Figure 5H and 5I). Previous studies showed that hesperetin suppresses proliferation and induces apoptosis by inducing *NOTCH1* expression in human gastrointestinal cancer cells.^57^ In addition, hesperetin activated the Notch1 signaling pathway and suppressed the proliferation of HTh7 anaplastic thyroid cancer cells.^58^ Taken together, the molecular mechanism of hesperetin in inhibition of BCSCs through Notch signaling needs to be explored further.

Peroxisome proliferator-activated receptor-gamma (PPAR-γ) or PPARG is a nuclear receptor that regulates the biological process of lipid metabolism.^59^ Recent studies highlight the emerging role of PPARG in cancer biology, including regulating cell-cycle arrest, apoptosis, angiogenesis, invasion, and cell migration.^60^ Activation of PPARG inhibits stemness in glioblastoma and colorectal cancer cells.^61,62^ Activation of PPARG inhibits metastasis by blocking β-catenin signaling and inhibiting MMP9 expression and activity.^63^ Activators of PPARG inhibit *MMP9* expression via inhibition of NFkB activation.^64^ Activation of PPARG by a PPARG agonist inhibits migration and invasion in hepatocellular carcinoma.^65^ The results of this present study showed inhibition of migration, upregulation of PPARG and downregulation of MMP9 upon hesperetin treatment (Figures 4E, 5E, and 5I), which is supported by previous studies. Hesperidin is a glycoside form of hesperetin that showed cardioprotective activity in diabetic rats via activation of PPARG.^66^ Hesperetin activates PPARG in adipocyte differentiation.^67^ A review article discussed the possibility that PPARG agonists might inhibit cell proliferation by regulating several pathways that are hallmarks of cancer, for instance, PI3K/mTOR, MAPK, and Wnt/ß-catenin.^68^ Moreover, a bioinformatics study demonstrated the potential of hesperetin as PPARG agonist for anticancer drugs.^69^ Taken together, these results suggest that PPARG plays an important role in hesperetin activity against BCSCs. However, the molecular mechanisms involved need to be clarified further.

Crosstalk between p53, PPARG and Notch signaling is also an interesting topic for future study. Activation of PPARG induces cell cycle arrest and apoptosis in MCF-7 cells via crosstalk between PPARG and p53.^70^ This study showed mRNA upregulation of *PPARG* and downregulation of *β-catenin* upon hesperetin treatment in mammospheres (Figure 5E and 5I). This result is supported by a previous study that demonstrated that a PPARG agonist induces β-catenin inhibition in type-2 diabetes and colon cancer.^71^ A study by Yun et al showed that PPARG promotes tumor suppressor activity by inhibition of cell proliferation via increasing p21 and downregulation of β-catenin and stem-cell-mediated eradication of MMP2 and MMP9 activity.^63^ Notch signaling also appears to be an important regulator of PPARλ.^72^ Taken together, these results suggest that p53, PPARG, and Notch signaling are potential targets of hesperetin in inhibition of BCSCs, but the molecular mechanisms involved need further investigation.

## Conclusion

In this study, using a bioinformatics approach, we identified P53, PPARG, and Notch signaling as potential targets of hesperetin in the inhibition of BCSCs. Moreover, an *in vitro* study showed that hesperetin exhibits cytotoxicity on mammospheres, inhibits mammosphere and colony formation, and inhibits migration. Moreover, hesperetin treatment modulates the expression of *p53, PPARG,* and * NOTCH1*. Taken together, these results suggest that hesperetin has potential for BCSC treatment by targeting p53, PPARG and Notch signaling. Further investigation of the molecular mechanisms involved is required for the development of hesperetin as a BCSC-targeted drug.

## Ethical Issues

Not applicable.

## Conflict of Interest

The authors declare no conflict of interest.

## Acknowledgments

The authors thank Badan Penerbit dan Publikasi Universitas Gadjah Mada Yogyakarta for their assistance in writing the manuscript.

## Funding

This work was supported by the Penelitian Unggulan Perguruan Tinggi (PUPT) 2017 and 2018, Contract No. 2398/UN1.P.III/DIT-LIT/LT/2017) and No. 189/UN1/DITLIT/DIT-LIT/LT/2018.

## Supplementary Materials

Supplementary file 1 contains Tables S1-S6.

## References

[1] Sharma S, Sharma D (2018). Intelligently applying artificial intelligence in chemoinformatics. Curr Top Med Chem.

[2] Hermawan A, Putri H, Utomo RY (2020). Comprehensive bioinformatics study reveals targets and molecular mechanism of hesperetin in overcoming breast cancer chemoresistance. Mol Divers.

[3] Gong L, Zhang D, Dong Y, Lei Y, Qian Y, Tan X (2018). Integrated bioinformatics analysis for identificating the therapeutic targets of aspirin in small cell lung cancer. J Biomed Inform.

[4] Azamjah N, Soltan-Zadeh Y, Zayeri F (2019). Global trend of breast cancer mortality rate: a 25-year study. Asian Pac J Cancer Prev.

[5] Yang F, Xu J, Tang L, Guan X (2017). Breast cancer stem cell: the roles and therapeutic implications. Cell Mol Life Sci.

[6] Dittmer J (2018). Breast cancer stem cells: features, key drivers and treatment options. Semin Cancer Biol.

[7] Palomeras S, Ruiz-Martínez S, Puig T (2018). Targeting breast cancer stem cells to overcome treatment resistance. Molecules.

[8] Dandawate PR, Subramaniam D, Jensen RA, Anant S (2016). Targeting cancer stem cells and signaling pathways by phytochemicals: novel approach for breast cancer therapy. Semin Cancer Biol.

[9] Palit S, Kar S, Sharma G, Das PK (2015). Hesperetin induces apoptosis in breast carcinoma by triggering accumulation of ROS and activation of ASK1/JNK pathway. J Cell Physiol.

[10] Sambantham S, Radha M, Paramasivam A, Anandan B, Malathi R, Chandra SR (2013). Molecular mechanism underlying hesperetin-induced apoptosis by in silico analysis and in prostate cancer PC-3 cells. Asian Pac J Cancer Prev.

[11] Adan A, Baran Y (2015). The pleiotropic effects of fisetin and hesperetin on human acute promyelocytic leukemia cells are mediated through apoptosis, cell cycle arrest, and alterations in signaling networks. Tumour Biol.

[12] Zhang J, Wu D, Vikash Vikash, Song J, Wang J, Yi J (2015). Hesperetin induces the apoptosis of gastric cancer cells via activating mitochondrial pathway by increasing reactive oxygen species. Dig Dis Sci.

[13] Wu D, Zhang J, Wang J, Li J, Liao F, Dong W (2016). Hesperetin induces apoptosis of esophageal cancer cells via mitochondrial pathway mediated by the increased intracellular reactive oxygen species. Tumour Biol.

[14] Smina TP, Mohan A, Ayyappa KA, Sethuraman S, Krishnan UM (2015). Hesperetin exerts apoptotic effect on A431 skin carcinoma cells by regulating mitogen activated protein kinases and cyclins. Cell Mol Biol (Noisy-le-grand).

[15] Bawazeer NA, Choudhry H, Zamzami MA, Abdulaal WH, Middleton B, Moselhy SS (2016). Role of hesperetin in LDL-receptor expression in hepatoma HepG2 cells. BMC Complement Altern Med.

[16] Aranganathan S, Selvam JP, Nalini N (2008). Effect of hesperetin, a citrus flavonoid, on bacterial enzymes and carcinogen-induced aberrant crypt foci in colon cancer rats: a dose-dependent study. J Pharm Pharmacol.

[17] Choi EJ, Kim GH (2011). Anti-/pro-apoptotic effects of hesperetin against 7,12-dimetylbenz(a)anthracene-induced alteration in animals. Oncol Rep.

[18] Babukumar S, Vinothkumar V, Velu P, Ramachandhiran D (2018). Hesperetin on cell surface glycoconjugates abnormalities and immunohistochemical staining with cytokeratin in 7,12 dimethylbenz(a)anthracene induced Hamster Buccal pouch carcinogenesis. Indian J Clin Biochem.

[19] Bodduluru LN, Kasala ER, Barua CC, Karnam KC, Dahiya V, Ellutla M (2015). Antiproliferative and antioxidant potential of hesperetin against benzo(a)pyrene-induced lung carcinogenesis in Swiss albino mice. Chem Biol Interact.

[20] Sarmoko Sarmoko, Dewi Pamungkas Putri D, Susidarti RA, Nugroho AE, Meiyanto E (2014). Increasing sensitivity of MCF-7/DOX cells towards doxorubicin by hesperetin through suppression of P-glycoprotein expression. Indones J Pharm.

[21] Wang Y, Liu S, Dong W, Qu X, Huang C, Yan T (2019). Combination of hesperetin and platinum enhances anticancer effect on lung adenocarcinoma. Biomed Pharmacother.

[22] Kim SY, Lee JY, Park YD, Kang KL, Lee JC, Heo JS (2013). Hesperetin alleviates the inhibitory effects of high glucose on the osteoblastic differentiation of periodontal ligament stem cells. PLoS One.

[23] Subash-Babu P, Alshatwi AA (2015). Hesperetin inhibit adipocyte differentiation and enhance Bax- and p21-mediated adipolysis in human mesenchymal stem cell adipogenesis. J Biochem Mol Toxicol.

[24] Kuhn M, von Mering C, Campillos M, Jensen LJ, Bork P (2008). STITCH: interaction networks of chemicals and proteins. Nucleic Acids Res.

[25] Szklarczyk D, Franceschini A, Wyder S, Forslund K, Heller D, Huerta-Cepas J (2015). STRING v10: protein-protein interaction networks, integrated over the tree of life. Nucleic Acids Res.

[26] Oliveros JC. Venny. An interactive tool for comparing lists with Venn’s diagrams. 2007. Available from: http://bioinfogp.cnb.csic.es/tools/venny/index.html.

[27] Shannon P, Markiel A, Ozier O, Baliga NS, Wang JT, Ramage D (2003). Cytoscape: a software environment for integrated models of biomolecular interaction networks. Genome Res.

[28] Chin CH, Chen SH, Wu HH, Ho CW, Ko MT, Lin CY (2014). cytoHubba: identifying hub objects and sub-networks from complex interactome. BMC Syst Biol.

[29] Huang DW, Sherman BT, Lempicki RA (2009). Bioinformatics enrichment tools: paths toward the comprehensive functional analysis of large gene lists. Nucleic Acids Res.

[30] Cerami E, Gao J, Dogrusoz U, Gross BE, Sumer SO, Aksoy BA (2012). The cBio cancer genomics portal: an open platform for exploring multidimensional cancer genomics data. Cancer Discov.

[31] Gao J, Aksoy BA, Dogrusoz U, Dresdner G, Gross B, Sumer SO (2013). Integrative analysis of complex cancer genomics and clinical profiles using the cBioPortal. Sci Signal.

[32] Grimshaw MJ, Cooper L, Papazisis K, Coleman JA, Bohnenkamp HR, Chiapero-Stanke L (2008). Mammosphere culture of metastatic breast cancer cells enriches for tumorigenic breast cancer cells. Breast Cancer Res.

[33] Oak PS, Kopp F, Thakur C, Ellwart JW, Rapp UR, Ullrich A (2012). Combinatorial treatment of mammospheres with trastuzumab and salinomycin efficiently targets HER2-positive cancer cells and cancer stem cells. Int J Cancer.

[34] Pickl M, Ries CH (2009). Comparison of 3D and 2D tumor models reveals enhanced HER2 activation in 3D associated with an increased response to trastuzumab. Oncogene.

[35] Mosmann T (1983). Rapid colorimetric assay for cellular growth and survival: application to proliferation and cytotoxicity assays. J Immunol Methods.

[36] Guzmán C, Bagga M, Kaur A, Westermarck J, Abankwa D (2014). ColonyArea: an ImageJ plugin to automatically quantify colony formation in clonogenic assays. PLoS One.

[37] Abdollahi P, Ebrahimi M, Motamed N, Samani FS (2015). Silibinin affects tumor cell growth because of reduction of stemness properties and induction of apoptosis in 2D and 3D models of MDA-MB-468. Anticancer Drugs.

[38] Pereira B, Chin SF, Rueda OM, Vollan HK, Provenzano E, Bardwell HA (2016). The somatic mutation profiles of 2,433 breast cancers refines their genomic and transcriptomic landscapes. Nat Commun.

[39] Yan X, Qi M, Li P, Zhan Y, Shao H (2017). Apigenin in cancer therapy: anti-cancer effects and mechanisms of action. Cell Biosci.

[40] Li Y, Kandhare AD, Mukherjee AA, Bodhankar SL (2019). Acute and sub-chronic oral toxicity studies of hesperidin isolated from orange peel extract in Sprague Dawley rats. Regul Toxicol Pharmacol.

[41] Rota LM, Lazzarino DA, Ziegler AN, LeRoith D, Wood TL (2012). Determining mammosphere-forming potential: application of the limiting dilution analysis. J Mammary Gland Biol Neoplasia.

[42] Shaw FL, Harrison H, Spence K, Ablett MP, Simões BM, Farnie G (2012). A detailed mammosphere assay protocol for the quantification of breast stem cell activity. J Mammary Gland Biol Neoplasia.

[43] Rajendran V, Jain MV (2018). In vitro tumorigenic assay: colony forming assay for cancer stem cells. Methods Mol Biol.

[44] Luo M, Brooks M, Wicha MS (2015). Epithelial-mesenchymal plasticity of breast cancer stem cells: implications for metastasis and therapeutic resistance. Curr Pharm Des.

[45] Taubenberger AV, Girardo S, Träber N, Fischer-Friedrich E, Kräter M, Wagner K (2019). Hydrogels: 3D microenvironment stiffness regulates tumor spheroid growth and mechanics via p21 and ROCK (Adv Biosys 9/2019). Adv Biosyst.

[46] Joshi I, Minter LM, Telfer J, Demarest RM, Capobianco AJ, Aster JC (2009). Notch signaling mediates G1/S cell-cycle progression in T cells via cyclin D3 and its dependent kinases. Blood.

[47] Chen J (2016). The cell-cycle arrest and apoptotic functions of p53 in tumor initiation and progression. Cold Spring Harb Perspect Med.

[48] Aloni-Grinstein R, Shetzer Y, Kaufman T, Rotter V (2014). p53: the barrier to cancer stem cell formation. FEBS Lett.

[49] Shetzer Y, Solomon H, Koifman G, Molchadsky A, Horesh S, Rotter V (2014). The paradigm of mutant p53-expressing cancer stem cells and drug resistance. Carcinogenesis.

[50] Ferreira de Oliveira JMP, Santos C, Fernandes E (2020). Therapeutic potential of hesperidin and its aglycone hesperetin: cell cycle regulation and apoptosis induction in cancer models. Phytomedicine.

[51] Kopan R (2012). Notch signaling. Cold Spring Harb Perspect Biol.

[52] Xiao YF, Yong X, Tang B, Qin Y, Zhang JW, Zhang D (2016). Notch and Wnt signaling pathway in cancer: crucial role and potential therapeutic targets (review). Int J Oncol.

[53] Xiao W, Gao Z, Duan Y, Yuan W, Ke Y (2017). Notch signaling plays a crucial role in cancer stem-like cells maintaining stemness and mediating chemotaxis in renal cell carcinoma. J Exp Clin Cancer Res.

[54] Venkatesh V, Nataraj R, Thangaraj GS, Karthikeyan M, Gnanasekaran A, Kaginelli SB (2018). Targeting Notch signalling pathway of cancer stem cells. Stem Cell Investig.

[55] Monahan P, Rybak S, Raetzman LT (2009). The notch target gene HES1 regulates cell cycle inhibitor expression in the developing pituitary. Endocrinology.

[56] Matsuno Y, Kiwamoto T, Morishima Y, Ishii Y, Hizawa N, Hogaboam CM (2018). Notch signaling regulates cell density-dependent apoptosis of NIH 3T3 through an IL-6/STAT3 dependent mechanism. Eur J Cell Biol.

[57] Zarebczan B, Pinchot SN, Kunnimalaiyaan M, Chen H (2011). Hesperetin, a potential therapy for carcinoid cancer. Am J Surg.

[58] Patel PN, Yu XM, Jaskula-Sztul R, Chen H (2014). Hesperetin activates the Notch1 signaling cascade, causes apoptosis, and induces cellular differentiation in anaplastic thyroid cancer. Ann Surg Oncol.

[59] Elix C, Pal SK, Jones JO (2018). The role of peroxisome proliferator-activated receptor gamma in prostate cancer. Asian J Androl.

[60] Vallée A, Lecarpentier Y (2018). Crosstalk between peroxisome proliferator-activated receptor gamma and the canonical WNT/β-catenin pathway in chronic inflammation and oxidative stress during carcinogenesis. Front Immunol.

[61] Pestereva E, Kanakasabai S, Bright JJ (2012). PPARγ agonists regulate the expression of stemness and differentiation genes in brain tumour stem cells. Br J Cancer.

[62] Moon CM, Kwon JH, Kim JS, Oh SH, Jin Lee K, Park JJ (2014). Nonsteroidal anti-inflammatory drugs suppress cancer stem cells via inhibiting PTGS2 (cyclooxygenase 2) and NOTCH/HES1 and activating PPARG in colorectal cancer. Int J Cancer.

[63] Yun SH, Han SH, Park JI (2018). Peroxisome proliferator-activated receptor γ and PGC-1α in cancer: dual actions as tumor promoter and suppressor. PPAR Res.

[64] Hetzel M, Walcher D, Grüb M, Bach H, Hombach V, Marx N (2003). Inhibition of MMP-9 expression by PPARgamma activators in human bronchial epithelial cells. Thorax.

[65] Hsu HT, Chi CW (2014). Emerging role of the peroxisome proliferator-activated receptor-gamma in hepatocellular carcinoma. J Hepatocell Carcinoma.

[66] Agrawal YO, Sharma PK, Shrivastava B, Ojha S, Upadhya HM, Arya DS (2014). Hesperidin produces cardioprotective activity via PPAR-γ pathway in ischemic heart disease model in diabetic rats. PLoS One.

[67] Gamo K, Miyachi H, Nakamura K, Matsuura N (2014). Hesperetin glucuronides induce adipocyte differentiation via activation and expression of peroxisome proliferator-activated receptor-γ. Biosci Biotechnol Biochem.

[68] Vella V, Nicolosi ML, Giuliano S, Bellomo M, Belfiore A, Malaguarnera R (2017). PPAR-γ agonists as antineoplastic agents in cancers with dysregulated IGF axis. Front Endocrinol (Lausanne).

[69] Gurula H, Loganathan T, Vashum Y, Pannerselvam S, Vetrivel U, Samuel S (2016). In silico screening of potent PPARgamma agonists among natural anticancer compounds of Indian origin. Asian J Pharm Clin Res.

[70] Bonofiglio D, Aquila S, Catalano S, Gabriele S, Belmonte M, Middea E (2006). Peroxisome proliferator-activated receptor-gamma activates p53 gene promoter binding to the nuclear factor-kappaB sequence in human MCF7 breast cancer cells. Mol Endocrinol.

[71] Lecarpentier Y, Claes V, Vallée A, Hébert JL (2017). Interactions between PPARgamma and the canonical Wnt/beta-catenin pathway in type 2 diabetes and colon cancer. PPAR Res.

[72] Call M, Fischesser K, Lunn M, Kao W (2013). Notch regulation of PPAR-gamma and development of meibomian gland dysfunction. Invest Ophthalmol Vis Sci.

