# Supplementary material for: Bioinformatics and In Vitro Studies Reveal the Importance of p53, PPARG and Notch Signaling Pathway in Inhibition of Breast Cancer Stem Cells by Hesperetin
Source: Adv Pharm Bull. 2020 Apr 19;11(2):351–60. doi: 10.34172/apb.2021.033 (PMC8046396; doi:10.34172/apb.2021.033)
Supplement: Supplementary file 1 — contains Tables S1-S6. [file apb-11-351-s001.pdf]

Supplementary file 1

Table S1. Primer sequences

**Gene name**	**Forward primer**	**Reverse primer**
Oct-4	5'-TCAGGTTGGACTGGGCCTAGT-3'	5'-GGAGGTTCCCTCTGAGTTGCTT-3'
Sox2	5'-GAGGGCTGGACTGCGAACT-3'	5'-TTTGCACCCCTCCCAATTC- 3'
Nanog	5´-GAAATCCCTTCCCTCGCCATC-3'	5'-CTCAGTAGCAGACCCTTGTAAGC-3'
ALDH1	5′-CTGCTGGCGACAATGGAGT-3'	5′-GTCAGCCCAACCTGCACAG-3′
CD133	5′-CAACCCTGAACTGAG GCAGC-3′	5′-TTGATAGCCCTGTTGGACCAG-3′
Survivin	5′-CAATTTGCCAAGCTCT GA-3′	5′-AGA TGGTCGTTTGGCTGAAT-3′
Vimentin	5′-TGAATACCAAGACCTGCTCAA-3´	5′-ATCAACCAGAGGGAGTGAAT C-3'
BAX	5´- GGGTGGTTGGGTGAGACTC -3´	5´-AGACACGTAAGGAAAACGCATTA-3'
BCL2	5´-TCCGCATCAGGAAGGCTAGA-3´	5´-AGGACCAGGCCTCCAAGCT-3'
caspase7	5′-CTACCGCCGTGGGAACGATGGCAGA-3'	5′-CGAAGGCCCATACCTGTCACTTTATC-3′
Cyclin D1	5′-AGGCCCTGGCTGCTACAAG-3′	5′-ACATCTGAGTGGGTCTGGAG-3′
PCNA	5′-CTGAAGCCGAAACAGCTAGACT-3′	5′-TCGTTGATGAGGTCTTGAGTGC-3′
CATB	5′-GCTTTCAGTTGAGCTGACCA-3′	5′-CAAGTCCA AGAT CAGC AGTC TC-3′
E-cadherin	5′-TCGTCACCACAAATCCAG TG-3′	5′-CATTCACATCCAGCACATCC-3′
MMP-9	5'-CCTGCCAGTTTCCATTCATC-3'	5'-GCCATTCACGTCGTCCTTAT-3'
P21	5'-GTCACTGTCTTGTACCCTTGTG-3'	5'-CGGCGTTTGGAGTGGTAGAAA-3'
P53	5'-GAGCTGAATGAGGCCTTGGA-3'	5'-CTGAGTCAGGCCCTTCTGTCTT-3'
NOTCH1	5'-CTGGTCAGGGAAATCGTG-3'	5'-TGGGCAGTGGCAGATGTAG-3'
HES1	5'-AGTGAAGCACCTCCGGAAC-3'	5'-CGTTCATGCACTCGCTGA-3'
PPARG	5´-TCT GGC CCA CCA ACT TTG GG-3'	5-CTT CAC AAG CAT GAA CTC CA-3'
CMYC	5´-GGA GGC TAT TCT GCC CAT TTG-3'	5´- GAG GCT GCT GGT TTT CCA CTA-3'
GAPDH	5′-ACGGGAAGCTCACTGGCA TGG-3′,	5′-GGTCCACCACCCTGTTGCTG TA-3′

Table S2. Direct protein targets and indirect protein targets of hesperetin.

Direct Protein Target	Indirect Protein Targets
SOAT1	DHCR24, CYP7A1, CH25H, LCAT, STS, CYP46A1, CYP11A1, DHCR7, ABCA1, SOAT2, VPS52, ABCG1, UBIAD1, HMGCR, AWAT1, MBOAT4, CYP27A1, APOE, SULT2B1, DGAT2
SOAT2	ABCG8, DHCR24, CYP7A1, CH25H, LCAT, STS, CYP46A1, CYP11A1, SOAT1, DHCR7, NPC1L1, VPS52, KRT2, ABCG5, AWAT1, AGPAT3, CYP27A1, APOB, SULT2B1, IGFBP6
MTTP	APOA2, APOA1, LDLR, APOB, PCSK9, ABCG8, DGAT1, APOA4, LPL, FABP1, P4HB, INS, NPC1L1, SREBF1, APOO, ABCG5, MLXIPL, NR0B2, DGAT2, APOC3
SHBG	IGF1, IGFBP3, AR, INS, LEP, ADIPOQ, ESR1, ALB, KLK3, PRL, CYP19A1, SERPINA7, GNRH1, SERPINA6, SULT2A1, CRP, IGFBP1
CDK2	CCNB2, MCM2, CCNA2, CDKN1A, CCNE1, CCND3, CKS1B, CCNE2, RB1, MCM4, CCNB1, CDC25A, PCNA, TP53, CCND2, CDKN1B, CDKN1C, RBL2, CCND1, CDC6
NOTCH1	MAML1, MAML2, RBPJ, MAML3, PSEN2, CTNNB1, HEY1, NUMB, PSEN1, HEY2, MYC, HES5, POFUT1, ADAM17, MFNG, ADAM10, DTX1, CCND1, HES1, LFNG
CDK4	CDKN1A, CCND2, CCNE1, CCND3, RB1, CCNA2, CDKN2A, E2F4, CDC25A, CCNB1, CDKN2B, CDKN2C, CDKN1B, RBL1, IFNAR1, CDKN1C, SMAD3, CDKN2D, PRMT5, CCND1
PPARG	NCOA3, NCOA1, NCOA2, RXRA, ADIPOQ, LEP, EP300, CEBPB, NCOR1, PLIN1, NCOR2, RXRG, TNF, MED1, RXRB, CEBPA, LPL, PPARGC1A, CREBBP, FABP4
ADIPOQ	PPARG, RXRA, LEP, EP300, CEBPB, NCOR1, NCOR2, IL6, CEBPA, CREBBP, INS, PPARGC1A, PRKAA2, NR3C1, ADIPOR2, ADIPOR1, CRP, FABP4, MLXIPL, RETN
PPARA	NCOA1, EP300, LPL, NCOR1, ACOX1, APOA5, NCOR2, CPT2, NRIP1, HELZ2, FABP1, CPT1A, MED1, HMGCS2, ACADM, PPARGC1A, ANGPTL4, FADS1, CREBBP, DUT
DGAT1	MOGAT1, CD36, PPAP2C, AGPAT2, PPAP2A, DHRS4, PPAPDC1B, PPAPDC1A, AWAT2, MOGAT3, DGAT2, PNPLA2, LPIN1, DEGS1, PLD1, SLC27A2, MOGAT2, RPE65, ITGAV, CYP26A1

Table S3. BCSC regulatory genes, retrieved from PubMed with the keywords “breast cancer stem cells“

No	tax_id	Org_name	Symbol	Aliases	description
1	9606	Homo sapiens	BRCA1	BRCAI, BRCC1, BROVCA1, FANCS, IRIS, PNCA4, PPP1R53, PSCP, RNF53	BRCA1 DNA repair associated
2	9606	Homo sapiens	TERT	CMM9, DKCA2, DKCB4, EST2, PFBMFT1, TCS1, TP2, TRT, hEST2, hTRT	telomerase reverse transcriptase
3	9606	Homo sapiens	ESR1	ER, ESR, ESRA, ESTRR, Era, NR3A1	estrogen receptor 1
4	9606	Homo sapiens	ABCG2	ABC15, ABCP, BCRP, BCRP1, BMDP, CD338, CDw338, EST157481, GOUT1, MRX, MXR, MXR-1, MXR1, UAQTL1	ATP binding cassette subfamily G member 2 (Junior blood group)
5	9606	Homo sapiens	CCND1	BCL1, D11S287E, PRAD1, U21B31	cyclin D1
6	9606	Homo sapiens	FGFR2	BBDS, BEK, BFR-1, CD332, CEK3, CFD1, ECT1, JWS, K-SAM, KGFR, TK14, TK25	fibroblast growth factor receptor 2
7	9606	Homo sapiens	PTHLH	BDE2, HHM, PLP, PTHR, PTHRP	parathyroid hormone like hormone
8	9606	Homo sapiens	CDKN2A	ARF, CDK4I, CDKN2, CMM2, INK4, INK4A, MLM, MTS-1, MTS1, P14, P14ARF, P16, P16-INK4A, P16INK4, P16INK4A, P19, P19ARF, TP16	cyclin dependent kinase inhibitor 2A
9	9606	Homo sapiens	MRTFA	BSAC, MAL, MKL, MKL1, MRTF-A	myocardin related transcription factor A
10	9606	Homo sapiens	MAP3K1	MAPKKK1, MEKK, MEKK 1, MEKK1, SRXY6	mitogen-activated protein kinase kinase kinase 1
11	9606	Homo sapiens	ERBB4	ALS19, HER4, p180erbB4	erb-b2 receptor tyrosine kinase 4
12	9606	Homo sapiens	CDKN2B	CDK4I, INK4B, MTS2, P15, TP15, p15INK4b	cyclin dependent kinase inhibitor 2B
13	9606	Homo sapiens	IGF1	IGF, IGF-I, IGFI, MGF	insulin like growth factor 1
14	9606	Homo sapiens	MYC	MRTLC, bHLHe39, c-Myc, MYC	MYC proto-oncogene, bHLH transcription factor
15	9606	Homo sapiens	KLF4	EZF, GKLF	Kruppel like factor 4
16	9606	Homo sapiens	TGFBR2	AAT3, FAA3, LDS1B, LDS2, LDS2B, MFS2, RIIC, TAAD2, TBR-ii, TBRII, TGFR-2, TGFbeta-RII	transforming growth factor beta receptor 2
17	9606	Homo sapiens	TCF7L2	TCF-4, TCF4	transcription factor 7 like 2
18	9606	Homo sapiens	TP53	BCC7, BMFS5, LFS1, P53, TRP53	tumor protein p53
19	9606	Homo sapiens	EGFR	ERBB, ERBB1, HER1, NISBD2, PIG61, mENA	epidermal growth factor receptor
20	9606	Homo sapiens	TNF	DIF-alpha, TNFA, TNFSF2, TNLG1F, TNF	tumor necrosis factor

21	9606	Homo sapiens	VEGFA	MVCD1, VEGF, VPF	vascular endothelial growth factor A
22	9606	Homo sapiens	IL6	BSF-2, BSF2, CDF, HGF, HSF, IFN-beta-2, IFNB2, IL-6	interleukin 6
23	9606	Homo sapiens	TGFB1	CED, DPD1, IBDIMDE, LAP, TGF-beta1, TGFB, TGFbeta	transforming growth factor beta 1
24	9606	Homo sapiens	HIF1A	HIF-1-alpha, HIF-1A, HIF-1alpha, HIF1, HIF1-ALPHA, MOP1, PASD8, bHLHe78	hypoxia inducible factor 1 subunit alpha
25	9606	Homo sapiens	DCLK1	CL1, CLICK1, DCAMKL1, DCDC3A, DCLK	doublecortin like kinase 1
26	9606	Homo sapiens	NFKB1	CVID12, EBP-1, KBF1, NF-kB, NF-kB1, NF-kappa-B1, NF-kappaB, NFKB-p105, NFKB-p50, NFkappaB, p105, p50	nuclear factor kappa B subunit 1
27	9606	Homo sapiens	AKT1	AKT, CWS6, PKB, PKB-ALPHA, PRKBA, RAC, RAC-ALPHA	AKT serine/threonine kinase 1
28	9606	Homo sapiens	STAT3	ADMIO, ADMIO1, APRF, HIES	signal transducer and activator of transcription 3
29	9606	Homo sapiens	IL1B	IL-1, IL1-BETA, IL1F2, IL1beta	interleukin 1 beta
30	9606	Homo sapiens	CTNNB1	CTNNB, EVR7, MRD19, NEDSDV, armadillo	catenin beta 1
31	9606	Homo sapiens	PTEN	10q23del, BZS, CWS1, DEC, GLM2, MHAM, MMAC11, PTENbeta, TEP1, PTEN	phosphatase and tensin homolog
32	9606	Homo sapiens	CXCL8	GCP-1, GCP1, IL8, LECT, LUCT, LYNAP, MDNCF, MONAP, NAF, NAP-1, NAP1	C-X-C motif chemokine ligand 8
33	9606	Homo sapiens	MTOR	FRAP, FRAP1, FRAP2, RAFT1, RAPT1, SKS	mechanistic target of rapamycin kinase
34	9606	Homo sapiens	CXCR4	CD184, D2S201E, FB22, HM89, HSY3RR, LAP-3, LAP3, LCR1, LESTR, NPY3R, NPYR, NPYRL, NPYY3R, WHIM, WHIMS	C-X-C motif chemokine receptor 4
35	9606	Homo sapiens	IFNG	IFG, IFI	interferon gamma
36	9606	Homo sapiens	JAK2	JTK10, THCYT3	Janus kinase 2
37	9606	Homo sapiens	MAPK1	ERK, ERK-2, ERK2, ERT1, MAPK2, P42MAPK, PRKM1, PRKM2, p38, p40, p41, p41mapk, p42-MAPK	mitogen-activated protein kinase 1
38	9606	Homo sapiens	CDKN1A	CAP20, CDKN1, CIP1, MDA-6, P21, SDI1, WAF1, p21CIP1	cyclin dependent kinase inhibitor 1A
39	9606	Homo sapiens	CD44	CDW44, CSPG8, ECMR-III, HCELL, HUTCH-I, IN, LHR, MC56, MDU2, MDU3, MIC4, Pgp1	CD44 molecule (Indian blood group)

40	9606	Homo sapiens	NOTCH1	AOS5, AOVD1, TAN1, hN1	notch receptor 1
41	9606	Homo sapiens	CXCL12	IRH, PBSF, SCYB12, SDF1, TLSF, TPAR1	C-X-C motif chemokine ligand 12
42	9606	Homo sapiens	MIR21	MIRN21, hsa-mir-21, miR-21, miRNA21	microRNA 21
43	9606	Homo sapiens	SIRT1	SIR2, SIR2L1, SIR2alpha	sirtuin 1
44	9606	Homo sapiens	TLR4	ARMD10, CD284, TLR-4, TOLL	toll like receptor 4
45	9606	Homo sapiens	GSK3B		glycogen synthase kinase 3 beta
46	9606	Homo sapiens	IGF1R	CD221, IGFIR, IGFR, JTK13	insulin like growth factor 1 receptor
47	9606	Homo sapiens	EZH2	ENX-1, ENX1b, KMT6, KMT6A, WVS, WVS2, EZH2	enhancer of zeste 2 polycomb repressive complex 2 subunit
48	9606	Homo sapiens	ITGB1	CD29, FNRB, GPIIA, MDF2, MSK12, VLA-BETA, VLAB	integrin subunit beta 1
49	9606	Homo sapiens	TP63	AIS, B(p51A), B(p51B), EEC3, KET, LMS, NBP, OFC8, RHS, SHFM4, TP53CP, TP53L, TP73L, p40, p51, p53CP, p63, p73H, p73L	tumor protein p63
50	9606	Homo sapiens	PTK2	FADK, FAK, FAK1, FRNK, PPP1R71, p125FAK, pp125FAK	protein tyrosine kinase 2
51	9606	Homo sapiens	HGF	DFNB39, F-TCFB, HPTA, SF, HGF	hepatocyte growth factor
52	9606	Homo sapiens	NTN1	MRMV4L, NTN1	netrin 1
53	9606	Homo sapiens	FGF2	BFGF, FGF-2, FGFB, HBGF-2	fibroblast growth factor 2
54	9606	Homo sapiens	YAP1	COB1, YAP, YAP2, YAP65, YKI	Yes associated protein 1
55	9606	Homo sapiens	CD274	B7-H, B7H1, PD-L1, PDCD1L1, PDCD1LG1, PDL1, hPD-L1	CD274 molecule
56	9606	Homo sapiens	EGF	HOMG4, URG	epidermal growth factor
57	9606	Homo sapiens	GJA1	AVSD3, CMDR, CX43, EKVP, EKVP3, GJAL, HLHS1, HSS, ODDD, PPKCA	gap junction protein alpha 1
58	9606	Homo sapiens	NFE2L2	HEBP1, IMDDHH, NRF2, Nrf-2	nuclear factor, erythroid 2 like 2
59	9606	Homo sapiens	MET	AUTS9, DFNB97, HGFR, RCCP2, c-Met	MET proto-oncogene, receptor tyrosine kinase
60	9606	Homo sapiens	POU5F1	OCT3, OCT4, OTF-3, OTF3, OTF4, Oct-3, Oct-4	POU class 5 homeobox 1
61	9606	Homo sapiens	ABCB1	ABC20, CD243, CLCS, GP170, MDR1, P-GP, PGY1	ATP binding cassette subfamily B member 1
62	9606	Homo sapiens	SOX2	ANOP3, MCOPS3	SRY-box transcription factor 2
63	9606	Homo sapiens	MIR34A	MIRN34A, miRNA34A, mir-34, mir-34a	microRNA 34a

64	9606	Homo sapiens	BMI1	FLVI2/BMI1, PCGF4, RNF51, flvi-2/bmi-1	BMI1 proto-oncogene, polycomb ring finger
65	9606	Homo sapiens	RUNX2	AML3, CBF-alpha-1, CBFA1, CCD, CCD1, CLCD, OSF-2, OSF2, PEA2aA, PEBP2aA	RUNX family transcription factor 2
66	9606	Homo sapiens	EPAS1	ECYT4, HIF2A, HLF, MOP2, PASD2, bHLHe73	endothelial PAS domain protein 1
67	9606	Homo sapiens	CDH1	Arc-1, BCDS1, CD324, CDHE, ECAD, LCAM, UVO	cadherin 1
68	9606	Homo sapiens	SOX9	CMD1, CMPD1, SRA1, SRXX2, SRXY10	SRY-box transcription factor 9
69	9606	Homo sapiens	ALDH1A1	ALDC, ALDH-E1, ALDH1, ALDH11, HEL-9, HEL-S-53e, HEL12, PUMB1, RALDH1	aldehyde dehydrogenase 1 family member A1
70	9606	Homo sapiens	RUNX1	AML1, AML1-EVI-1, AMLCR1, CBF2alpha, CBFA2, EVI-1, PEBP2aB, PEBP2alpha	RUNX family transcription factor 1
71	9606	Homo sapiens	ERBB2	CD340, HER-2, HER-2/neu, HER2, MLN 19, NEU, NGL, TKR1	erb-b2 receptor tyrosine kinase 2
72	9606	Homo sapiens	NES	Nbla00170	nestin
73	9606	Homo sapiens	IL10	CSIF, GVHDS, IL-10A, TGIF, IL10	interleukin 10
74	9606	Homo sapiens	CD24	CD24A	CD24 molecule
75	9606	Homo sapiens	KIT	C-Kit, CD117, MASTC, PBT, SCFR	KIT proto-oncogene, receptor tyrosine kinase
76	9606	Homo sapiens	NANOG		Nanog homeobox
77	9606	Homo sapiens	HLA-G	MHC-G	major histocompatibility complex, class I, G
78	9606	Homo sapiens	MAPK3	ERK-1, ERK1, ERT2, HS44KDAP, HUMKER1A, P44ERK1, P44MAPK, PRKM3, p44-ERK1, p44-MAPK	mitogen-activated protein kinase 3
79	9606	Homo sapiens	PPARG	CIMT1, GLM1, NR1C31, PPARG2, PPARgamma, PPARG	peroxisome proliferator activated receptor gamma
80	9606	Homo sapiens	EWSR1	EWS, EWS-FLI1, bK984G1.4	EWS RNA binding protein 1
81	9606	Homo sapiens	WNT1	BMND16, INT1, OI15	Wnt family member 1
82	9606	Homo sapiens	SMAD2	JV18, JV18-1, MADH2, MADR2, hMAD-2, hSMAD2	SMAD family member 2
83	9606	Homo sapiens	GLI1	GLI, PAPA8, PPD1	GLI family zinc finger 1
84	9606	Homo sapiens	ITPR1	ACV, CLA4, INSP3R1, IP3R, IP3R1, PPP1R94, SCA15, SCA16, SCA29	inositol 1,4,5-trisphosphate receptor type 1
85	9606	Homo sapiens	ATM	AT1, ATA, ATC, ATD, ATDC, ATE, TEL1, TELO1	ATM serine/threonine kinase
86	9606	Homo sapiens	FOXO3	AF6q21, FKHRL1, FKHRL1P2, FOXO2A, FOXO3	forkhead box O3

87	9606	Homo sapiens	BMP7	OP-1	bone morphogenetic protein 7
88	9606	Homo sapiens	IDO1	IDO, IDO-1, INDO	indoleamine 2,3-dioxygenase 1
89	9606	Homo sapiens	AR	AIS8, DHTR, HUMARA, HYSP1, KD, NR3C4, SBMA, SMAX1, TFM, AR	androgen receptor
90	9606	Homo sapiens	MAPK14	CSBP, CSBP1, CSBP2, CSPB1, EXIP, Mxi2, PRKM14, PRKM15, RK, SAPK2A, p38, p38ALPHA	mitogen-activated protein kinase 14
91	9606	Homo sapiens	FGF4	FGF-4, HBGF-4, HST, HST-1, HSTF-1, HSTF1, K-FGF, KFGF	fibroblast growth factor 4
92	9606	Homo sapiens	TET2	KIAA1546, MDS	tet methylcytosine dioxygenase 2
93	9606	Homo sapiens	MIR145	MIRN145, miR-145, miRNA145	microRNA 145
94	9606	Homo sapiens	WNT5A	hWNT5A	Wnt family member 5A
95	9606	Homo sapiens	FOXQ1	HFH1	forkhead box Q1
96	9606	Homo sapiens	LEP	LEPD, OB, OBS	leptin
97	9606	Homo sapiens	H19	ASM, ASM1, BWS, D11S813E, LINC00008, MIR675HG, NCRNA00008, WT2	H19 imprinted maternally expressed transcript
98	9606	Homo sapiens	MIR146A	MIRN146, MIRN146A, miR-146a, miRNA146A	microRNA 146a
99	9606	Homo sapiens	TAB2	CHTD2, MAP3K7IP2, TAB-2	TGF-beta activated kinase 1 (MAP3K7) binding protein 2
100	9606	Homo sapiens	SHH	HHG1, HLP3, HPE3, MCOPCB5, SMMCI, ShhNC, TPT, TPTPS	sonic hedgehog signaling molecule
101	9606	Homo sapiens	ZNF703	NLZ1, ZEPPO1, ZNF503L, ZPO1	zinc finger protein 703
102	9606	Homo sapiens	ITGA6	CD49fB, VLA-6, ITGA6	integrin subunit alpha 6
103	9606	Homo sapiens	HMOX1	HMOX1D, HO-1, HSP32, bK286B10	heme oxygenase 1
104	9606	Homo sapiens	FOXM1	FKHL16A, FOXM1B, FOXM1C, HFH-11, HFH11, HNF-3, INS-1, MPHOSPH2, MPP-2, MPP2, PIG29, TRIDENT, FOXM1	forkhead box M1
105	9606	Homo sapiens	MMP9	CLG4B, GELB, MANDP2, MMP-9	matrix metallopeptidase 9
106	9606	Homo sapiens	KRAS	'C-K-RAS, C-K-RAS, CFC2, K-RAS2A, K-RAS2B, K-RAS4A, K-RAS4B, K-Ras, K-Ras 2, KI-RAS1, KRAS2, NS, NS3, OES, RALD, RASK2, c-Ki-ras, c-Ki-ras2, KRAS	KRAS proto-oncogene, GTPase
107	9606	Homo sapiens	PTGS2	COX-2, COX2, GRIPGHS, PGG/HS, PGHS-2, PHS-2, hCox-2	prostaglandin-endoperoxide synthase 2

108	9606	Homo sapiens	BMP4	BMP2B, BMP2B1, MCOPS6, OFC11, ZYME	bone morphogenetic protein 4
109	9606	Homo sapiens	HOTAIR	HOXAS, HOXC-AS4, HOXC11-AS1, NCRNA00072	HOX transcript antisense RNA
110	9606	Homo sapiens	DNMT3B	ICF, ICF1, M.HsaIIIB	DNA methyltransferase 3 beta
111	9606	Homo sapiens	BCL2	Bcl-2, PPP1R50	BCL2 apoptosis regulator
112	9606	Homo sapiens	SMAD4	DPC4, JIP, MADH4, MYHRS	SMAD family member 4
113	9606	Homo sapiens	KRT19	CK19, K19, K1CS	keratin 19
114	9606	Homo sapiens	SMAD3	HSPC193, HsT17436, JV15-2, LDS1C, LDS3, MADH3	SMAD family member 3
115	9606	Homo sapiens	BCL2L1	BCL-XL/S, BCL2L, BCLX, Bcl-X, PPP1R52	BCL2 like 1
116	9606	Homo sapiens	BIRC5	API4, EPR-1	baculoviral IAP repeat containing 5
117	9606	Homo sapiens	CTH		cystathionine gamma-lyase
118	9606	Homo sapiens	IL17A	CTLA-8, CTLA8, IL-17, IL-17A, IL17	interleukin 17A
119	9606	Homo sapiens	WWTR1	TAZ	WW domain containing transcription regulator 1
120	9606	Homo sapiens	RELA	CMCU, NFKB3, p65	RELA proto-oncogene, NF-kB subunit
121	9606	Homo sapiens	DKK1	DKK-1, SK	dickkopf WNT signaling pathway inhibitor 1
122	9606	Homo sapiens	SPP1	BNSP, BSPI, ETA-1, OPN	secreted phosphoprotein 1
123	9606	Homo sapiens	ZEB1	AREB6, BZP, DELTAEF1, FECD6, NIL2A, PPCD3, TCF8, ZFHEP, ZFHX1A	zinc finger E-box binding homeobox 1
124	9606	Homo sapiens	CDKN1B	CDKN4, KIP1, MEN1B, MEN4, P27KIP1	cyclin dependent kinase inhibitor 1B
125	9606	Homo sapiens	RAC1	MIG5, MRD48, Rac-1, TC-25, p21-Rac1	Rac family small GTPase 1
126	9606	Homo sapiens	KDR	CD309, FLK1, VEGFR, VEGFR2	kinase insert domain receptor
127	9606	Homo sapiens	NOS3	ECNOS, eNOS	nitric oxide synthase 3
128	9606	Homo sapiens	RHOA	ARH12, ARHA, RHO12, RHOH12	ras homolog family member A
129	9606	Homo sapiens	ITGA5	CD49e, FNRA, VLA-5, VLA5A	integrin subunit alpha 5
130	9606	Homo sapiens	LIN28A	CSDD1, LIN-28, LIN28, ZCCHC1, lin-28A	lin-28 homolog A
131	9606	Homo sapiens	HDAC6	CPBHM, HD6, JM21, PPP1R90	histone deacetylase 6
132	9606	Homo sapiens	FLT1	FLT, FLT-1, VEGFR-1, VEGFR1	fms related tyrosine kinase 1
133	9606	Homo sapiens	CCL5	D17S136E, RANTES, SCYA5, SIS-delta, SISd, TCP228, eoCP	C-C motif chemokine ligand 5
134	9606	Homo sapiens	PROM1	AC133, CD133, CORD12, MCDR2, MSTP061, PROML1, RP41, STGD4	prominin 1

135	9606	Homo sapiens	MMP2	CLG4, CLG4A, MMP-2, MMP-II, MONA, TBE-1	matrix metallopeptidase 2
136	9606	Homo sapiens	DNMT1	ADCADN, AIM, CXXC9, DNMT, HSN1E, MCMT, m.HsaI	DNA methyltransferase 1
137	9606	Homo sapiens	SERPINE1	PAI, PAI-1, PAI1, PLANH1	serpin family E member 1
138	9606	Homo sapiens	PIK3CA	CLAPO, CLOVE, CWS5, MCAP, MCM, MCMTC, PI3K, PI3K-alpha, p110-alpha	phosphatidylinositol-4,5-bisphosphate 3-kinase catalytic subunit alpha
139	9606	Homo sapiens	VCAM1	CD106, INCAM-100	vascular cell adhesion molecule 1
140	9606	Homo sapiens	ICAM1	BB2, CD54, P3.58	intercellular adhesion molecule 1
141	9606	Homo sapiens	PKM	CTHBP, HEL-S-30, OIP3, PK32, TCB, THBP1, p58, PKM	pyruvate kinase M1/2
142	9606	Homo sapiens	CDK4	CMM3, PSK-J3	cyclin dependent kinase 4
143	9606	Homo sapiens	CAV1	BSCL3, CGL3, LCCNS, MSTP085, PPH3, VIP21	caveolin 1
144	9606	Homo sapiens	JUN	AP-1, AP1, c-Jun, cJUN, p39	Jun proto-oncogene, AP-1 transcription factor subunit
145	9606	Homo sapiens	LMNA	CDCD1, CDDC, CMD1A, CMT2B1, EMD2, FPL, FPLD, FPLD2, HGPS, IDC, LDP1, LFP, LGMD1B, LMN1, LMNC, LMNL1, MADA, PRO1	lamin A/C
146	9606	Homo sapiens	NGFR	CD271, Gp80-LNGFR, TNFRSF16, p75(NTR), p75NTR	nerve growth factor receptor
147	9606	Homo sapiens	NRP1	BDCA4, CD304, NP1, NRP, VEGF165R	neuropilin 1
148	9606	Homo sapiens	SNAI2	SLUG, SLUGH, SLUGH1, SNAIL2, WS2D	snail family transcriptional repressor 2
149	9606	Homo sapiens	PRKCA	AAG6, PKC-alpha, PKCA, PKCI+/-, PKCalpha, PRKACA	protein kinase C alpha
150	9606	Homo sapiens	KDM1A	AOF2, BHC110, CPRF, KDM1, LSD1	lysine demethylase 1A
151	9606	Homo sapiens	HMGA1	HMG-RA, HMGIY, HMGA1	high mobility group AT-hook 1
152	9606	Homo sapiens	MIR29A	MIRN29, MIRN29A, hsa-mir-29, hsa-mir-29a, miRNA29A, mir-29a	microRNA 29a
153	9606	Homo sapiens	LEPR	CD295, LEP-RD, OB-R, OBR, LEPR	leptin receptor
154	9606	Homo sapiens	MIR155	MIRN155, miRNA155, mir-155	microRNA 155
155	9606	Homo sapiens	IGF2	C11orf43, GRDF, IGF-II, PP9974	insulin like growth factor 2
156	9606	Homo sapiens	MIR27A	MIR27, MIRN27A, mir-27a	microRNA 27a
157	9606	Homo sapiens	BMP2	BDA2A, SSFSC, BMP2	bone morphogenetic protein 2
158	9606	Homo sapiens	MDM2	ACTFS, HDMX, hdm2	MDM2 proto-oncogene

159	9606	Homo sapiens	CCL2	GDCF-2, HC11, HSMCR30, MCAF, MCP-1, MCP1, SCYA2, SMC-CF	C-C motif chemokine ligand 2
160	9606	Homo sapiens	ITGAV	CD51, MSK8, VNRA, VTNR	integrin subunit alpha V
161	9606	Homo sapiens	CTLA4	ALPS5, CD, CD152, CELIAC3, CTLA-4, GRD4, GSE, IDDM12	cytotoxic T-lymphocyte associated protein 4
162	9606	Homo sapiens	LGR5	FEX, GPR49, GPR67, GRP49, HG38	leucine rich repeat containing G protein-coupled receptor 5
163	9606	Homo sapiens	LRRK2	AURA17, DARDARIN, PARK8, RIPK7, ROCO2	leucine rich repeat kinase 2
164	9606	Homo sapiens	TP53BP1	53BP1, TDRD30, p202, p53BP1	tumor protein p53 binding protein 1
165	9606	Homo sapiens	MMP1	CLG, CLGN	matrix metallopeptidase 1
166	9606	Homo sapiens	SP1		Sp1 transcription factor
167	9606	Homo sapiens	ADIPOQ	ACDC, ACRP30, ADIPQTL1, ADPN, APM-1, APM1, GBP28	adiponectin, C1Q and collagen domain containing
168	9606	Homo sapiens	BMP6	VGR, VGR1	bone morphogenetic protein 6
169	9606	Homo sapiens	BDNF	ANON2, BULN2	brain derived neurotrophic factor
170	9606	Homo sapiens	MAPK8	JNK, JNK-46, JNK1, JNK1A2, JNK21B1/2, PRKM8, SAPK1, SAPK1c	mitogen-activated protein kinase 8
171	9606	Homo sapiens	FN1	CIG, ED-B, FINC, FN, FNZ, GFND, GFND2, LETS, MSF, SMDCF	fibronectin 1
172	9606	Homo sapiens	HSPA5	BIP, GRP78, HEL-S-89n, MIF2	heat shock protein family A (Hsp70) member 5
173	9606	Homo sapiens	MCL1	BCL2L3, EAT-ES, MCL1L, MCL1S, Mcl-1, TM, bcl2-L-3, mcl1/EAT, MCL1	MCL1 apoptosis regulator, BCL2 family member
174	9606	Homo sapiens	TWIST1	ACS3, BPES2, BPES3, CRS, CRS1, CSO, SCS, SWCOS, TWIST, bHLHa38	twist family bHLH transcription factor 1
175	9606	Homo sapiens	NRG1	ARIA, GGF, GGF2, HGL, HRG, HRG1, HRGA, MST131, MSTP131, NDF-IT2, SMDF, NRG1	neuregulin 1
176	9606	Homo sapiens	FOXO1	FKH1, FKHRA, FOXO1	forkhead box O1
177	9606	Homo sapiens	CCR5	CC-CKR-5, CCCKR5, CCR-5, CD195, CKR-5, CKR5, CMKBR5, IDDM22	C-C motif chemokine receptor 5 (gene/pseudogene)
178	9606	Homo sapiens	NEWENTRY		Record to support submission of GeneRIFs for a gene not in Gene (human; man).
179	9606	Homo sapiens	ELAVL1	ELAV1, HUR, Hua, MelG	ELAV like RNA binding protein 1
180	9606	Homo sapiens	ERBB3	ErbB-3, FERLK, HER3, LCCS2, MDA-BF-1, c-	erb-b2 receptor tyrosine kinase 3

				erbB-3, c-erbB3, erbB3-S, p180-ErbB3, p45-sErbB3, p85-sErbB3	
181	9606	Homo sapiens	MIR125B1	MIRN125B1, mir-125b-1	microRNA 125b-1
182	9606	Homo sapiens	RUNX3	AML2, CBFA3, PEBP2aC	RUNX family transcription factor 3
183	9606	Homo sapiens	FAS	ALPS1A, APO-1, APT1, CD951, FASTM, TNFRSF6, FAS	Fas cell surface death receptor
184	9606	Homo sapiens	ESR2	ER-BETA, ESR-BETA, ESRB, ESTRB, Erb, NR3A2, ODG8	estrogen receptor 2
185	9606	Homo sapiens	CEBPB	C/EBP-beta, IL6DBP, NF-IL6, TCF5	CCAAT enhancer binding protein beta
186	9606	Homo sapiens	POSTN	OSF-2, OSF2, PDLPOSTN, PN	periostin
187	9606	Homo sapiens	RHOC	ARH9, ARHC, H9, RHOH9	ras homolog family member C
188	9606	Homo sapiens	FOXP3	AIID, DIETER, IPEX, JM2, PIDX, XPID	forkhead box P3
189	9606	Homo sapiens	CD34		CD34 molecule
190	9606	Homo sapiens	AGER	RAGE, SCARJ1	advanced glycosylation end-product specific receptor
191	9606	Homo sapiens	CASP3	CPP32, CPP32B, SCA-1	caspase 3
192	9606	Homo sapiens	TNFSF10	APO2L, Apo-2L, CD253, TL2, TNLG6A, TRAIL	TNF superfamily member 10
193	9606	Homo sapiens	MIR126	MIRN126, miRNA126, mir-126	microRNA 126
194	9606	Homo sapiens	BAX	BCL2L4	BCL2 associated X, apoptosis regulator
195	9606	Homo sapiens	LGALS3	CBP35, GAL3, GALBP, GALIG, L31, LGALS2, MAC2	galectin 3
196	9606	Homo sapiens	ACE	ACE1, CD143, DCP, DCP1	angiotensin I converting enzyme
197	9606	Homo sapiens	AXL	ARK, JTK11, Tyro7, UFO	AXL receptor tyrosine kinase
198	9606	Homo sapiens	JAG1	AGS, AGS1, AHD, AWS, CD339, DCHE, HJ1, JAGL1	jagged canonical Notch ligand 1
199	9606	Homo sapiens	MKI67	KIA, MIB-, MIB-1, PPP1R105	marker of proliferation Ki-67
200	9606	Homo sapiens	COL1A1	EDSARTH1, EDSC, OI1, OI2, OI3, OI4	collagen type I alpha 1 chain
201	9606	Homo sapiens	MIR200C	MIRN200C, mir-200c	microRNA 200c
202	9606	Homo sapiens	TLR9	CD289	toll like receptor 9
203	9606	Homo sapiens	F3	CD142, TF, TFA	coagulation factor III, tissue factor
204	9606	Homo sapiens	MIR143	MIRN143, mir-143	microRNA 143
205	9606	Homo sapiens	IGFBP3	BP-53, IBP3	insulin like growth factor binding protein 3
206	9606	Homo sapiens	AURKA	AIK, ARK1, AURA, BTAK, PPP1R47, STK15,	aurora kinase A

				STK6, STK7	
207	9606	Homo sapiens	TP73	P73	tumor protein p73
208	9606	Homo sapiens	PLK1	PLK, STPK13	polo like kinase 1
209	9606	Homo sapiens	BSG	5F7, CD147, EMMPRIN, EMPRIN, OK, SLC7A11, TCSF	basigin (Ok blood group)
210	9606	Homo sapiens	APEX1	APE, APE1, APEN, APEX, APX, HAP1, REF1	apurinic/apyrimidinic endodeoxyribonuclease 1
211	9606	Homo sapiens	TIMP1	CLGI, EPA, EPO, HCI, TIMP, TIMP-1	TIMP metallopeptidase inhibitor 1
212	9606	Homo sapiens	FGFR1	BFGFR, CD331, CEK, ECCL, FGFBR, FGFR-1, FLG, FLT-2, FLT2, HBGFR, HH2, HRTFDS, KAL2, N-SAM, OGD, bFGF-R-1	fibroblast growth factor receptor 1
213	9606	Homo sapiens	REST	DFNA27, GINGF5, HGF5, NRSF, WT6, XBR	RE1 silencing transcription factor
214	9606	Homo sapiens	MAPT	DDPAC, FTDP-17L, MSTD, MTBT1, MTBT2, PPND, PPP1R103, TAU, MAPT	microtubule associated protein tau
215	9606	Homo sapiens	MIRLET7A1	LET7A1, MIRNLET7A1, let-7a-1	microRNA let-7a-1
216	9606	Homo sapiens	MIR124-1	MIR124A, MIR124A1, MIRN124-1, MIRN124A1, mir-124-1	microRNA 124-1
217	9606	Homo sapiens	MIR125A	MIRN125A, miRNA125A, mir-125a	microRNA 125a
218	9606	Homo sapiens	TGM2	TG(C), TGC	transglutaminase 2
219	9606	Homo sapiens	CDC42	CDC42Hs, G25K, TKS	cell division cycle 42
220	9606	Homo sapiens	WNT3A		Wnt family member 3A
221	9606	Homo sapiens	CHEK1	CHK1	checkpoint kinase 1
222	9606	Homo sapiens	TGFBR1	AAT5, ACVRLK4, ALK-5, ALK5, ESS1, LDS1, LDS1A, LDS2A, MSSE, SKR4, TBR-i, TBRI, TGFR-1, tbetaR-I	transforming growth factor beta receptor 1
223	9606	Homo sapiens	MIR200A	MIRN200A, mir-200a	microRNA 200a
224	9606	Homo sapiens	APOE	AD2, APO-E, ApoE4, LDLCQ5, LPG	apolipoprotein E
225	9606	Homo sapiens	PRKAA1	AMPK, AMPKa1	protein kinase AMP-activated catalytic subunit alpha 1
226	9606	Homo sapiens	HMGA2	BABL, HMGI-C, HMGIC, LIPO, STQTL9	high mobility group AT-hook 2
227	9606	Homo sapiens	ABCC1	ABC29, ABCC, GS-X, MRP, MRP1	ATP binding cassette subfamily C member 1
228	9606	Homo sapiens	GATA3	HDR, HDRS	GATA binding protein 3
229	9606	Homo sapiens	EPCAM	DIAR5, EGP-2, EGP314, EGP40, ESA, HNPCC8,	epithelial cell adhesion molecule

				KS1/4, KSA, M4S1, MIC18, MK-1, TACSTD1, TROP1	
230	9606	Homo sapiens	HMGB1	HMG-1, HMG1, HMG3, SBP-1	high mobility group box 1
231	9606	Homo sapiens	CA9	CAIX, MN	carbonic anhydrase 9
232	9606	Homo sapiens	RB1	OSRC, PPP1R130, RB, p105-Rb, p110-RB1, pRb, pp110	RB transcriptional corepressor 1
233	9606	Homo sapiens	FBXW7	AGO, CDC4, FBW6, FBW7, FBX30, FBXO30, FBXW6, SEL-10, SEL10, hAgo, hCdc4	F-box and WD repeat domain containing 7
234	9606	Homo sapiens	ITGB3	BDPLT16, BDPLT2, CD61, GP3A, GPIIIa, GT	integrin subunit beta 3
235	9606	Homo sapiens	PARP1	ADPRT, ADPRT 1, ADPRT1, ARTD1, PARP, PARP-1, PPOL, pADPRT-1	poly(ADP-ribose) polymerase 1
236	9606	Homo sapiens	AGTR1	AG2SB, AT1, AT1AR, AT1B, AT1BR, AT1R, AT2R1, HAT1R, AGTR1	angiotensin II receptor type 1
237	9606	Homo sapiens	APC	BTPS2, DP2, DP2.5, DP3, GS, PPP1R46	APC regulator of WNT signaling pathway
238	9606	Homo sapiens	KRT18	CK-18, CYK18, K18	keratin 18
239	9606	Homo sapiens	CYP19A1	ARO, ARO1, CPV1, CYAR, CYP19, CYPXIX, P-450AROM	cytochrome P450 family 19 subfamily A member 1
240	9606	Homo sapiens	MIF	GIF, GLIF, MMIF	macrophage migration inhibitory factor
241	9606	Homo sapiens	SLC9A1	APNH, LIKNS, NHE-1, NHE1, PPP1R143	solute carrier family 9 member A1
242	9606	Homo sapiens	NOS2	HEP-NOS, INOS, NOSA, NOS2	nitric oxide synthase 2
243	9606	Homo sapiens	MIR210	MIRN210, mir-210	microRNA 210
244	9606	Homo sapiens	ADAM12	ADAM12-OT1, CAR10, MCMP, MCMPMltna, MLTN, MLTNA	ADAM metallopeptidase domain 12
245	9606	Homo sapiens	CASP8	ALPS2B, CAP4, Casp-8, FLICE, MACH, MCH5	caspase 8
246	9606	Homo sapiens	ABL1	ABL, BCR-ABL, CHDSKM, JTK7, bcr/abl, c-ABL, c-ABL1, p150, v-abl	ABL proto-oncogene 1, non-receptor tyrosine kinase
247	9606	Homo sapiens	MCAM	CD146, HEMCAM, METCAM, MUC18, MelCAM	melanoma cell adhesion molecule
248	9606	Homo sapiens	HDAC1	GON-10, HD1, KDAC1, RPD3, RPD3L1	histone deacetylase 1
249	9606	Homo sapiens	ACKR3	CMKOR1, CXC-R7, CXCR-7, CXCR7, GPR159, RDC-1, RDC1	atypical chemokine receptor 3
250	9606	Homo sapiens	LDHA	GSD11, HEL-S-133P, LDHM, PIG19	lactate dehydrogenase A
251	9606	Homo sapiens	E2F1	E2F-1, RBAP1, RBBP3, RBP3	E2F transcription factor 1
252	9606	Homo sapiens	OGG1	HMMH, HOGG1, MUTM, OGH1	8-oxoguanine DNA glycosylase

253	9606	Homo sapiens	SLC2A1	CSE, DYT17, DYT18, DYT9, EIG12, GLUT, GLUT-1, GLUT1, GLUT1DS, HTLVR, PED, SDCHCN	solute carrier family 2 member 1
254	9606	Homo sapiens	FASLG	ALPS1B, APT1LG1, APTL, CD178, CD95-L, CD95L, FASL, TNFSF6, TNLG1A	Fas ligand
255	9606	Homo sapiens	CDK1	CDC2, CDC28A, P34CDC2	cyclin dependent kinase 1
256	9606	Homo sapiens	RAD51	BRCC5, FANCR, HRAD51, HsRad51, HsT16930, MRMV2A, RECA, RAD51	RAD51 recombinase
257	9606	Homo sapiens	PLAUR	CD87, U-PAR, UPAR, URKR	plasminogen activator, urokinase receptor
258	9606	Homo sapiens	AHR	RP85, bHLHe76	aryl hydrocarbon receptor
259	9606	Homo sapiens	NAMPT	1110035O14Rik, PBEF, PBEF1, VF, VISFATIN	nicotinamide phosphoribosyltransferase
260	9606	Homo sapiens	NF1	NFNS, VRNF, WSS	neurofibromin 1
261	9606	Homo sapiens	PIK3R1	AGM7, GRB1, IMD36, p85, p85-ALPHA	phosphoinositide-3-kinase regulatory subunit 1
262	9606	Homo sapiens	MMP14	MMP-14, MMP-X1, MT-MMP, MT-MMP 1, MT1-MMP, MT1MMP, MTMMP1, WNCHRS	matrix metallopeptidase 14
263	9606	Homo sapiens	CHI3L1	ASRT7, CGP-39, GP-39, GP39, HC-gp39, HCGP-3P, YK-40, YKL-40, YKL40, YYL-40, hCGP-39	chitinase 3 like 1
264	9606	Homo sapiens	PLAU	ATF, BDPLT5, QPD, UPA, URK, u-PA	plasminogen activator, urokinase
265	9606	Homo sapiens	NEAT1	LINC00084, NCRNA00084, TncRNA, VINC	nuclear paraspeckle assembly transcript 1
266	9606	Homo sapiens	VIM		vimentin
267	9606	Homo sapiens	XIAP	API3, BIRC4, IAP-3, ILP1, MIHA, XLP2, hIAP-3, hIAP3	X-linked inhibitor of apoptosis
268	9606	Homo sapiens	RASSF1	123F2, NORE2AA, RDA32, REH3P21, RASSF1	Ras association domain family member 1
269	9606	Homo sapiens	MIR148A	MIRN148, MIRN148A, hsa-mir-148, mir-148a	microRNA 148a
270	9606	Homo sapiens	DICER1	DCR1, Dicer, Dicer1e, GLOW, HERNA, K12H4.8-LIKE, MNG1, RMSE2	dicer 1, ribonuclease III
271	9606	Homo sapiens	EZR	CVIL, CVL, HEL-S-105, VIL2	ezrin
272	9606	Homo sapiens	MIR22	MIRN22, hsa-mir-22, miR-22	microRNA 22
273	9606	Homo sapiens	STK11	LKB1, PJS, hLKB1	serine/threonine kinase 11
274	9606	Homo sapiens	BECN1	ATG6, VPS30, beclin1	beclin 1
275	9606	Homo sapiens	ANXA2	ANX2, ANX2L4, CAL1H, HEL-S-270, LIP2, LPC2, LPC2D, P36, PAP-IV	annexin A2

276	9606	Homo sapiens	IGFBP5	IBP5	insulin like growth factor binding protein 5
277	9606	Homo sapiens	PAWR	PAR4, Par-4	pro-apoptotic WT1 regulator
278	9606	Homo sapiens	TOP2A	TOP2, TP2A	DNA topoisomerase II alpha
279	9606	Homo sapiens	PIN1	DOD, UBL5	peptidylprolyl cis/trans isomerase, NIMA-interacting 1
280	9606	Homo sapiens	SOCS3	ATOD4, CIS3, Cish3, SOCS-3, SSI-3, SSI3	suppressor of cytokine signaling 3
281	9606	Homo sapiens	MALAT1	HCN, LINC00047, NCRNA00047, NEAT2, PRO2853	metastasis associated lung adenocarcinoma transcript 1
282	9606	Homo sapiens	ROCK1	P160ROCK, ROCK-I	Rho associated coiled-coil containing protein kinase 1
283	9606	Homo sapiens	MIR101-1	MIRN101-1, mir-101-1	microRNA 101-1
284	9606	Homo sapiens	CCNE1	CCNE, pCCNE1	cyclin E1
285	9606	Homo sapiens	S100A4	18A2, 42A, CAPL, FSP1, MTS1, P9KA, PEL98	S100 calcium binding protein A4
286	9606	Homo sapiens	NGF	Beta-NGF, HSAN5B, NGF	nerve growth factor
287	9606	Homo sapiens	PIK3CG	PI3CG, PI3K, PI3Kgamma, PIK3, p110gamma, p120-PI3K	phosphatidylinositol-4,5-bisphosphate 3-kinase catalytic subunit gamma
288	9606	Homo sapiens	ETS1	ETS-1, EWSR2, c-ets-1, p54	ETS proto-oncogene 1, transcription factor
289	9606	Homo sapiens	PRL	GHA1	prolactin
290	9606	Homo sapiens	MIR100	MIRN100, miR-100	microRNA 100
291	9606	Homo sapiens	RPS6KB1	PS6K, S6K, S6K-beta-1, S6K1, STK14A, p70 S6KA, p70(S6K)-alpha, p70-S6K, p70-alpha	ribosomal protein S6 kinase B1
292	9606	Homo sapiens	ANXA1	ANX1, LPC1	annexin A1
293	9606	Homo sapiens	TNFRSF10B	CD262, DR5, KILLER, KILLER/DR5, TRAIL-R2, TRAILR2, TRICK2, TRICK2A, TRICK2B, TRICKB, ZTNFR9	TNF receptor superfamily member 10b
294	9606	Homo sapiens	IL18	IGIF, IL-18, IL-1g, IL1F4	interleukin 18
295	9606	Homo sapiens	MMP13	CLG3, MANDP1, MDST, MMP-13	matrix metallopeptidase 13
296	9606	Homo sapiens	STAT5A	MGF, STAT5	signal transducer and activator of transcription 5A
297	9606	Homo sapiens	ANGPT1	AGP1, AGPT, ANG1	angiopoietin 1
298	9606	Homo sapiens	GPER1	CEPR, CMKRL2, DRY12, FEG-1, GPCR-Br, GPER, GPR30, LERGU, LERGU2, LyGPR, mER	G protein-coupled estrogen receptor 1
299	9606	Homo sapiens	MIR221	MIRN221, miRNA221, mir-221	microRNA 221

300	9606	Homo sapiens	WWOX	D16S432E, EIEE28, FOR, FRA16D, HHCMA56, PRO0128, SCAR12, SDR41C1, WOX1	WW domain containing oxidoreductase
301	9606	Homo sapiens	ID1	ID, bHLHb24	inhibitor of DNA binding 1, HLH protein
302	9606	Homo sapiens	CCR7	BLR2, CC-CKR-7, CCR-7, CD197, CDw197, CMKBR7, EBI1	C-C motif chemokine receptor 7
303	9606	Homo sapiens	CCN1	CYR61, GIG1, IGFBP10	cellular communication network factor 1
304	9606	Homo sapiens	EPOR	EPO-R	erythropoietin receptor
305	9606	Homo sapiens	STAT5B	STAT5	signal transducer and activator of transcription 5B
306	9606	Homo sapiens	RARB	HAP, MCOPS12, NR1B2, RARbeta1, RRB2	retinoic acid receptor beta
307	9606	Homo sapiens	MIR203A	MIR203, MIRN203, hsa-mir-203a, miR-203, miRNA203, mir-203a	microRNA 203a
308	9606	Homo sapiens	CDK6	MCPH12, PLSTIRE	cyclin dependent kinase 6
309	9606	Homo sapiens	PIWIL2	CT80, HILI, PIWIL1L, mili	piwi like RNA-mediated gene silencing 2
310	9606	Homo sapiens	MGMT		O-6-methylguanine-DNA methyltransferase
311	9606	Homo sapiens	LOX	AAT10	lysyl oxidase
312	9606	Homo sapiens	CXCL1	FSP, GRO1, GROa, MGSA, MGSA-a, NAP-3, SCYB1	C-X-C motif chemokine ligand 1
313	9606	Homo sapiens	TNFRSF11A	CD265, FEO, LOH18CR1, ODFR, OFE, OPTB7, OSTS, PDB2, RANK, TRANCER	TNF receptor superfamily member 11a
314	9606	Homo sapiens	FOXA1	HNF3A, TCF3A	forkhead box A1
315	9606	Homo sapiens	FANCD2	FA-D2, FA4, FACD, FAD, FAD2, FANCD	FA complementation group D2
316	9606	Homo sapiens	NT5E	CALJA, CD73, E5NT, NT, NT5, NTE, eN, eNT	5'-nucleotidase ecto
317	9606	Homo sapiens	SLC5A5	NIS, TDH1	solute carrier family 5 member 5
318	9606	Homo sapiens	INSR	CD220, HHF5	insulin receptor
319	9606	Homo sapiens	MIR196A2	MIRN196-2, MIRN196A2, mir-196a-2	microRNA 196a-2
320	9606	Homo sapiens	MIR200B	MIRN200B, mir-200b	microRNA 200b
321	9606	Homo sapiens	MIR222	MIRN222, miRNA222, mir-222	microRNA 222
322	9606	Homo sapiens	MIR30A	MIRN30A, mir-30a	microRNA 30a
323	9606	Homo sapiens	MIR182	MIRN182, miRNA182, mir-182	microRNA 182
324	9606	Homo sapiens	PPARA	NR1C1, PPAR, PPARalpha, hPPAR	peroxisome proliferator activated receptor alpha
325	9606	Homo sapiens	FOXC1	ARA, ASGD3, FKHL7, FREAC-3, FREAC3, IGDA,	forkhead box C1

				IHG1, IRID1, RIEG3	
326	9606	Homo sapiens	GRN	CLN11, GEP, GP88, PCDGF, PEPI, PGRN	granulin precursor
327	9606	Homo sapiens	MIR141	MIRN141, mir-141	microRNA 141
328	9606	Homo sapiens	ESRRA	ERR1, ERRa, ERRalpha, ESRL1, NR3B1	estrogen related receptor alpha
329	9606	Homo sapiens	NR5A2	B1F, B1F2, CPF, FTF, FTZ-F1, FTZ-F1beta, LRH-1, LRH1, hB1F-2	nuclear receptor subfamily 5 group A member 2
330	9606	Homo sapiens	MIR10B	MIRN10B, hsa-mir-10b, miRNA10B, mir-10b	microRNA 10b
331	9606	Homo sapiens	CEBPA	C/EBP-alpha, CEBP	CCAAT enhancer binding protein alpha
332	9606	Homo sapiens	PRKDC	DNA-PKC, DNA-PKcs, DNAPK, DNAPKc, DNPK1, HYRC, HYRC1, IMD26, XRCC7, p350	protein kinase, DNA-activated, catalytic subunit
333	9606	Homo sapiens	MIR199A1	MIR-199-s, MIRN199A1, mir-199a-1	microRNA 199a-1
334	9606	Homo sapiens	TET1	CXXC6, LCX, bA119F7.1	tet methylcytosine dioxygenase 1
335	9606	Homo sapiens	MIR9-1	MIRN9-1, hsa-mir-9-1, miRNA9-1, mir-9-1	microRNA 9-1
336	9606	Homo sapiens	ADAM17	ADAM18, CD156B, CSVP, NISBD, NISBD1, TACE	ADAM metallopeptidase domain 17
337	9606	Homo sapiens	F2R	CF2R, HTR, PAR-1, PAR1, TR	coagulation factor II thrombin receptor
338	9606	Homo sapiens	MIR183	MIRN183, miR-183, miRNA183	microRNA 183
339	9606	Homo sapiens	SPARC	BM-40, OI17, ON	secreted protein acidic and cysteine rich
340	9606	Homo sapiens	SOCS1	CIS1, CISH1, JAB, SOCS-1, SSI-1, SSI1, TIP-3, TIP3	suppressor of cytokine signaling 1
341	9606	Homo sapiens	XRCC6	CTC75, CTCBF, G22P1, KU70, ML8, TLAA	X-ray repair cross complementing 6
342	9606	Homo sapiens	MIR96	DFNA50, MIRN96, hsa-mir-96, miR-96, miRNA96	microRNA 96
343	9606	Homo sapiens	MIR335	MIRN335, hsa-mir-335, miRNA335, mir-335	microRNA 335
344	9606	Homo sapiens	CDH2	CD325, CDHN, CDw325, NCAD	cadherin 2
345	9606	Homo sapiens	IGFBP2	IBP2, IGF-BP53	insulin like growth factor binding protein 2
346	9606	Homo sapiens	TRAF4	CART1, MLN62, RNF83	TNF receptor associated factor 4
347	9606	Homo sapiens	EDN1	ARCND3, ET1, HDLCQ7, PPET1, QME	endothelin 1
348	9606	Homo sapiens	SRA1	SRA, SRAP, STRAA1, pp7684	steroid receptor RNA activator 1
349	9606	Homo sapiens	HOXB4	HOX-2.6, HOX2, HOX2F	homeobox B4
350	9606	Homo sapiens	BAD	BBC2, BCL2L8	BCL2 associated agonist of cell death
351	9606	Homo sapiens	PHB	HEL-215, HEL-S-54e1, PHB	prohibitin
352	9606	Homo sapiens	SFRP1	FRP, FRP-1, FRP1, FrzA, SARP2	secreted frizzled related protein 1

353	9606	Homo sapiens	SCN5A	CDCD2, CMD1E, CMPD2, HB1, HB2, HBBD, HH1, ICCD, IVF, LQT3, Nav1.5, PFHB1, SSS1, VF1	sodium voltage-gated channel alpha subunit 5
354	9606	Homo sapiens	NOTCH4	INT3	notch receptor 4
355	9606	Homo sapiens	TLR3	CD283, IIAE2	toll like receptor 3
356	9606	Homo sapiens	HSPB1	CMT2F, HEL-S-102, HMN2B, HS.76067, HSP27, HSP28, Hsp25, SRP27	heat shock protein family B (small) member 1
357	9606	Homo sapiens	PRKCD	ALPS3, CVID9, MAY1, PKCD, nPKC-delta	protein kinase C delta
358	9606	Homo sapiens	HES1	HES-1, HHL, HRY, bHLHb39	hes family bHLH transcription factor 1
359	9606	Homo sapiens	NEK2	HsPK21A, NLK1, PPP1R111, RP67, NEK2	NIMA related kinase 2
360	9606	Homo sapiens	PDGFRA	CD140A, PDGFR-2, PDGFR2	platelet derived growth factor receptor alpha
361	9606	Homo sapiens	STC1	STC	stanniocalcin 1
362	9606	Homo sapiens	NOTCH3	CADASIL, CADASIL1, CASIL, IMF2, LMNS	notch receptor 3
363	9606	Homo sapiens	MIR139	MIR139-3p, MIRN139, mir-139	microRNA 139
364	9606	Homo sapiens	H2AX	H2A.X, H2A/X, H2AFX	H2A.X variant histone
365	9606	Homo sapiens	NODAL	HTX5	nodal growth differentiation factor
366	9606	Homo sapiens	TIMP2	CSC-21K, DDC8	TIMP metallopeptidase inhibitor 2
367	9606	Homo sapiens	FUT4	CD15, ELFT, FCT3A, FUC-TIV, FUTIV, LeX, SSEA-1	fucosyltransferase 4
368	9606	Homo sapiens	GDF2	BMP-9, BMP9, HHT5	growth differentiation factor 2
369	9606	Homo sapiens	ESRRG	ERR-gamma, ERR3, ERRg, ERRgamma, NR3B3	estrogen related receptor gamma
370	9606	Homo sapiens	MIR26B	MIRN26B, hsa-mir-26b, miR-26b	microRNA 26b
371	9606	Homo sapiens	PDGFB	IBGC5, PDGF-2, PDGF2, SIS, SSV, c-sis	platelet derived growth factor subunit B
372	9606	Homo sapiens	EPHA2	ARCC2, CTPA, CTPP1, CTRCT6, ECK	EPH receptor A2
373	9606	Homo sapiens	UCHL1	HEL-117, HEL-S-53, NDGOA, PARK5, PGP 9.5, PGP9.5, PGP95, SPG79, Uch-L1	ubiquitin C-terminal hydrolase L1
374	9606	Homo sapiens	TBX3	TBX3-ISO, UMS, XHL	T-box transcription factor 3
375	9606	Homo sapiens	ANXA3	ANX3	annexin A3
376	9606	Homo sapiens	THY1	CD90, CDw90	Thy-1 cell surface antigen
377	9606	Homo sapiens	DCN	CSCD, DSPG2, PG40, PGII, PGS2, SLRR1B	decorin
378	9606	Homo sapiens	CDH5	7B4, CD144	cadherin 5
379	9606	Homo sapiens	TACR1	NK1R, NKIR, SPR, TAC1R	tachykinin receptor 1
380	9606	Homo sapiens	GHRL	MTLRP	ghrelin and obestatin prepropeptide

381	9606	Homo sapiens	MIR17	MIR17-5p, MIR91, MIRN17, MIRN91, hsa-mir-17, miR-17, miR17-3p, miRNA17, miRNA91	microRNA 17
382	9606	Homo sapiens	RET	CDHF12, CDHR16, HSCR1, MEN2A, MEN2B, MTC1, PTC-ELE1, RET	ret proto-oncogene
383	9606	Homo sapiens	GJB2	CX26, DFNA3, DFNA3A, DFNB1, DFNB1A, HID, KID, NSRD1, PPK	gap junction protein beta 2
384	9606	Homo sapiens	MIR373	MIRN373, hsa-mir-373, miRNA373, mir-373	microRNA 373
385	9606	Homo sapiens	MIR31	MIRN31, hsa-mir-31, miR-31	microRNA 31
386	9606	Homo sapiens	MIR211	MIRN211, mir-211	microRNA 211
387	9606	Homo sapiens	BID	FP497	BH3 interacting domain death agonist
388	9606	Homo sapiens	ADIPOR1	ACDCR1, CGI-45, CGI45, PAQR1, TESBP1A	adiponectin receptor 1
389	9606	Homo sapiens	CCND2	KIAK0002, MPPH3	cyclin D2
390	9606	Homo sapiens	MIR195	MIRN195, miRNA195, mir-195	microRNA 195
391	9606	Homo sapiens	IL11	AGIF, IL-11	interleukin 11
392	9606	Homo sapiens	FLT3	CD135, FLK-2, FLK2, STK1	fms related tyrosine kinase 3
393	9606	Homo sapiens	FLI1	BDPLT21, EWSR2, SIC-1	Fli-1 proto-oncogene, ETS transcription factor
394	9606	Homo sapiens	OSM		oncostatin M
395	9606	Homo sapiens	FGFR3	ACH, CD333, CEK2, HSFGFR3EX, JTK4	fibroblast growth factor receptor 3
396	9606	Homo sapiens	CCN2	CTGF, HCS24, IGFBP8, NOV2	cellular communication network factor 2
397	9606	Homo sapiens	F11R	CD321, JAM, JAM1, JAMA, JCAM, KAT, PAM-1	F11 receptor
398	9606	Homo sapiens	SQSTM1	A170, DMRV, FTDALS3, NADGP, OSIL, PDB3, ZIP3, p60, p62, p62B	sequestosome 1
399	9606	Homo sapiens	PML	MYL, PP8675, RNF71, TRIM19	promyelocytic leukemia
400	9606	Homo sapiens	PDGFRB	CD140B, IBGC4, IMF1, JTK12, KOGS, PDGFR, PDGFR-1, PDGFR1, PENTT	platelet derived growth factor receptor beta
401	9606	Homo sapiens	MIR497	MIRN497, hsa-mir-497, mir-497	microRNA 497
402	9606	Homo sapiens	EGR1	AT225, G0S30, KROX-24, NGFI-A, TIS8, ZIF-268, ZNF225	early growth response 1
403	9606	Homo sapiens	KL	HFTC3	klotho
404	9606	Homo sapiens	MYD88	MYD88D	MYD88 innate immune signal transduction adaptor
405	9606	Homo sapiens	MIR494	MIRN494, hsa-mir-494, mir-494	microRNA 494

406	9606	Homo sapiens	MIR7-1	MIRN7-1, hsa-mir-7-1, mir-7-1	microRNA 7-1
407	9606	Homo sapiens	SERPINF1	EPC-1, OI12, OI6, PEDF, PIG35	serpin family F member 1
408	9606	Homo sapiens	LINC-ROR	ROR, lincRNA-RoR, lincRNA-ST8SIA3	long intergenic non-protein coding RNA, regulator of reprogramming
409	9606	Homo sapiens	TNC	150-225, DFNA56, GMEM, GP, HXB, JI, TN, TN-C	tenascin C
410	9606	Homo sapiens	TGFB2	G-TSF, LDS4, TGF-beta2	transforming growth factor beta 2
411	9606	Homo sapiens	MIR340	MIRN340, hsa-mir-340, mir-340	microRNA 340
412	9606	Homo sapiens	ABCB5	ABCB5alphabeta, EST422562, ABCB5	ATP binding cassette subfamily B member 5
413	9606	Homo sapiens	ZNF217	ZABC1	zinc finger protein 217
414	9606	Homo sapiens	ARID1A	B120, BAF250, BAF250a, BM029, C1orf4, CSS2, ELD, MRD14, OSA1, P270, SMARCF1, hELD, hOSA1	AT-rich interaction domain 1A
415	9606	Homo sapiens	CTNND1	BCDS2, CAS, CTNND, P120CAS, P120CTN, p120, p120(CAS), p120(CTN)	catenin delta 1
416	9606	Homo sapiens	MAP1LC3A	ATG8E, LC3, LC3A, MAP1ALC3, MAP1BLC3	microtubule associated protein 1 light chain 3 alpha
417	9606	Homo sapiens	DIABLO	DFNA64, SMAC	diablo IAP-binding mitochondrial protein
418	9606	Homo sapiens	NOTCH2	AGS2, HJCYS, hN2	notch receptor 2
419	9606	Homo sapiens	TAC1	Hs.2563, NK2, NKNA, NPK, TAC2	tachykinin precursor 1
420	9606	Homo sapiens	MIR137	MIRN137, miR-137	microRNA 137
421	9606	Homo sapiens	TGFB3	ARVD, ARVD1, LDS5, RNHF, TGF-beta3	transforming growth factor beta 3
422	9606	Homo sapiens	ADRB2	ADRB2R, ADRBR, B2AR, BAR, BETA2AR	adrenoceptor beta 2
423	9606	Homo sapiens	ENPP2	ATX, ATX-X, AUTOTAXIN, LysoPLD, NPP2, PD-IALPHA, PDNP2	ectonucleotide pyrophosphatase/phosphodiesterase 2
424	9606	Homo sapiens	XRCC4	SSMED	X-ray repair cross complementing 4
425	9606	Homo sapiens	IRS2	IRS-2	insulin receptor substrate 2
426	9606	Homo sapiens	MIR375	MIRN375, hsa-mir-375, miRNA375, mir-375	microRNA 375
427	9606	Homo sapiens	FOXP1	12CC4, HSPC215, MFH, QRF1, hFKH1B	forkhead box P1
428	9606	Homo sapiens	MIR101-2	MIRN101-2, mir-101-2	microRNA 101-2
429	9606	Homo sapiens	SCD	FADS5, MSTP0081, SCDOS, hSCD1, SCD	stearoyl-CoA desaturase
430	9606	Homo sapiens	MIR20A	C13orf25, MIR17HG, MIR20, MIRH1, MIRHG1, MIRN20, MIRN20A, hsa-mir-20, hsa-mir-20a, miR-20, miRNA20A, mir-20a	microRNA 20a

431	9606	Homo sapiens	EPHB4	CMAVM2, HFASD, HTK, LMPHM7, MYK1, TYRO11	EPH receptor B4
432	9606	Homo sapiens	HOXA9	ABD-B, HOX1, HOX1.7, HOX1G	homeobox A9
433	9606	Homo sapiens	DLL4	AOS6, delta4, hdelta2	delta like canonical Notch ligand 4
434	9606	Homo sapiens	HSP90AA1	EL52, HEL-S-65p, HSP86, HSP89A, HSP90A, HSP90N, HSPC1, HSPCA, HSPCAL1, HSPCAL4, HSPN, Hsp103, Hsp89, Hsp90, LAP-2, LAP2	heat shock protein 90 alpha family class A member 1
435	9606	Homo sapiens	PTPN11	BPTP3, CFC, JMML, METCDS, NS1, PTP-1D, PTP2C, SH-PTP2, SH-PTP3, SHP2	protein tyrosine phosphatase non-receptor type 11
436	9606	Homo sapiens	MIR19A	C13orf25, MIR17HG, MIRH1, MIRHG1, MIRN19A, hsa-mir-19a, miR-19a, miRNA19A	microRNA 19a
437	9606	Homo sapiens	MIR140	MIRN140, SEDN, miRNA140, mir-140	microRNA 140
438	9606	Homo sapiens	PODXL	Gp200, PC, PCLP, PCLP-1	podocalyxin like
439	9606	Homo sapiens	HMMR	CD168, IHABP, RHAMM	hyaluronan mediated motility receptor
440	9606	Homo sapiens	ENG	END, HHT1, ORW1	endoglin
441	9606	Homo sapiens	USP22	USP3L	ubiquitin specific peptidase 22
442	9606	Homo sapiens	EPO	DBAL, ECYT5, EP, MVCD2	erythropoietin
443	9606	Homo sapiens	DNMT3A	DNMT3A2, M.HsaIIIA, TBRS	DNA methyltransferase 3 alpha
444	9606	Homo sapiens	IL6R	CD126, IL-6R-1, IL-6RA, IL6QA, IL6RQ, gp80, IL6R	interleukin 6 receptor
445	9606	Homo sapiens	SIRT7	SIR2L7	sirtuin 7
446	9606	Homo sapiens	MIR125B2	MIRN125B2, mir-125b-2	microRNA 125b-2
447	9606	Homo sapiens	CASP9	APAF-3, APAF3, ICE-LAP6, MCH6, PPP1R56	caspase 9
448	9606	Homo sapiens	WRN	RECQ3, RECQL2, RECQL3	WRN RecQ like helicase
449	9606	Homo sapiens	RARA	NR1B1, RAR	retinoic acid receptor alpha
450	9606	Homo sapiens	ILK	HEL-S-28-1, ILK-2, P59, p59ILK, ILK	integrin linked kinase
451	9606	Homo sapiens	KEAP1	INrf2, KLHL19	kelch like ECH associated protein 1
452	9606	Homo sapiens	GATA4	ASD2, TACHD, TOF, VSD1	GATA binding protein 4
453	9606	Homo sapiens	PPP2CA	NEDLBA, PP2Ac, PP2CA, PP2Calpha, RP-C	protein phosphatase 2 catalytic subunit alpha
454	9606	Homo sapiens	ZEB2	HSPC082, SIP-1, SIP1, SMADIP1, ZFHX1B	zinc finger E-box binding homeobox 2
455	9606	Homo sapiens	KRT5	CK5, DDD, DDD1, EBS2, K5A, KRT5	keratin 5
456	9606	Homo sapiens	PITX2	ARP1, ASGD4, Brx1, IDG2, IGDS, IGDS2, IHG2,	paired like homeodomain 2

				IRID2, Otlx2, PTX2, RGS, RIEG, RIEG1, RS	
457	9606	Homo sapiens	SIPA1	SPA1	signal-induced proliferation-associated 1
458	9606	Homo sapiens	PIWIL4	HIWI2, MIWI2	piwi like RNA-mediated gene silencing 4
459	9606	Homo sapiens	MIR214	MIRN214, miRNA214, mir-214	microRNA 214
460	9606	Homo sapiens	KRT8	CARD2, CK-8, CK8, CYK8, K2C8, K8, KO	keratin 8
461	9606	Homo sapiens	MIR16-1	MIRN16-1, miRNA16-1, mir-16-1	microRNA 16-1
462	9606	Homo sapiens	SALL4	DRRS, HSAL4, ZNF797	spalt like transcription factor 4
463	9606	Homo sapiens	HLA-A	HLAA	major histocompatibility complex, class I, A
464	9606	Homo sapiens	MEG3	FP504, GTL2, LINC00023, NCRNA00023, PRO0518, PRO2160, onco-lncRNA-83, prebp1	maternally expressed 3
465	9606	Homo sapiens	DKK3	REIC, RIG	dickkopf WNT signaling pathway inhibitor 3
466	9606	Homo sapiens	IL4	BCGF-1, BCGF1, BSF-1, BSF1, IL-4	interleukin 4
467	9606	Homo sapiens	GAS5	NCRNA00030, SNHG2	growth arrest specific 5
468	9606	Homo sapiens	MIR29B1	MIRN29B1, miR-29b, miRNA29B1, mir-29b-1	microRNA 29b-1
469	9606	Homo sapiens	INS	IDDM, IDDM1, IDDM2, ILPR, IRDN, MODY10	insulin
470	9606	Homo sapiens	MVP	LRP, VAULT1	major vault protein
471	9606	Homo sapiens	PRKN	AR-JP, LPRS2, PARK2, PDJ	parkin RBR E3 ubiquitin protein ligase
472	9606	Homo sapiens	IDH1	HEL-216, HEL-S-26, IDCD, IDH, IDP, IDPC, PICD	isocitrate dehydrogenase (NADP(+)) 1
473	9606	Homo sapiens	IHH	BDA1, HHG2	Indian hedgehog signaling molecule
474	9606	Homo sapiens	GNL3	C77032, E2IG3, NNP47, NS	G protein nucleolar 3
475	9606	Homo sapiens	CXCL10	C7, IFI10, INP10, IP-10, SCYB10, crg-2, gIP-10, mob-1	C-X-C motif chemokine ligand 10
476	9606	Homo sapiens	MIR152	MIRN152, mir-152	microRNA 152
477	9606	Homo sapiens	MIR18A	C13orf25, MIR17HG, MIR18, MIRH1, MIRHG1, MIRN18, MIRN18A, hsa-mir-18, hsa-mir-18a, miR-18, miRNA18A, mir-18a	microRNA 18a
478	9606	Homo sapiens	GMNN	Gem, MGORS6	geminin DNA replication inhibitor
479	9606	Homo sapiens	SMYD3	KMT3E, ZMYND1, ZNFN3A1, bA74P14.1	SET and MYND domain containing 3
480	9606	Homo sapiens	CSNK2A1	CK2A1, CKII, Cka1, Cka2, OCNDS	casein kinase 2 alpha 1
481	9606	Homo sapiens	MIR146B	MIRN146B, miRNA146B, mir-146b	microRNA 146b
482	9606	Homo sapiens	LRIG1	LIG-1, LIG1	leucine rich repeats and immunoglobulin like domains 1
483	9606	Homo sapiens	MIR424	MIR322, MIRN424, hsa-mir-424, miRNA424, mir-	microRNA 424

				424	
484	9606	Homo sapiens	LGALS1	GAL1, GBP	galectin 1
485	9606	Homo sapiens	CCR2	CC-CKR-2, CCR-2A, CCR2B, CD192, CKR2, CKR2A, CKR2B, CMKBR2, MCP-1-R, CCR2	C-C motif chemokine receptor 2
486	9606	Homo sapiens	MIR19B1	C13orf25, MIR17HG, MIR19B, MIRH1, MIRHG1, MIRN19B1, miR-19b-1, miRNA19B1	microRNA 19b-1
487	9606	Homo sapiens	PAX6	AN, AN1, AN2, ASGD5, D11S812E, FVH1, MGDA, WAGR	paired box 6
488	9606	Homo sapiens	SHC1	SHC, SHCA	SHC adaptor protein 1
489	9606	Homo sapiens	MIR199A2	MIR-199-s, MIRN199A2, mir-199a-2	microRNA 199a-2
490	9606	Homo sapiens	UGCG	GCS, GLCT1	UDP-glucose ceramide glucosyltransferase
491	9606	Homo sapiens	IKBKB	IKK-beta, IKK2, IKKB, IMD15, IMD15A, IMD15B, NFKBIKB	inhibitor of nuclear factor kappa B kinase subunit beta
492	9606	Homo sapiens	MELK	HPK38	maternal embryonic leucine zipper kinase
493	9606	Homo sapiens	ITGA2	BR, CD49B, GPIa, HPA-5, VLA-2, VLAA2	integrin subunit alpha 2
494	9606	Homo sapiens	RXRA	NR2B1	retinoid X receptor alpha
495	9606	Homo sapiens	SMARCA4	BAF190, BAF190A, BRG1, CSS4, MRD16, RTPS2, SNF2, SNF2-beta, SNF2L4, SNF2LB, SWI2, hSNF2b	SWI/SNF related, matrix associated, actin dependent regulator of chromatin, subfamily a, member 4
496	9606	Homo sapiens	PREX1	P-REX1	phosphatidylinositol-3,4,5-trisphosphate dependent Rac exchange factor 1
497	9606	Homo sapiens	MME	CALLA, CD10, CMT2T, NEP, SCA43, SFE	membrane metalloendopeptidase
498	9606	Homo sapiens	BGLAP	BGP, OC, OCN	bone gamma-carboxyglutamate protein
499	9606	Homo sapiens	CXCR2	CD182, CDw128b, CMKAR2, IL8R2, IL8RA, IL8RB	C-X-C motif chemokine receptor 2
500	9606	Homo sapiens	IL27	IL-27, IL-27AA, IL27p28, IL30, p28, IL27	interleukin 27
501	9606	Homo sapiens	KAT2B	CAF, P/CAF, PCAF	lysine acetyltransferase 2B
502	9606	Homo sapiens	GATA1	ERYF1, GATA-1, GF-1, GF1, NF-E1, NFE1, XLANP, XLTDA, XLTT	GATA binding protein 1
503	9606	Homo sapiens	APP	AAA, ABETA, ABPP, AD1I, CTFgamma, CVAP, PN-II, PN2, preA4, APP	amyloid beta precursor protein
504	9606	Homo sapiens	PIM1	PIM	Pim-1 proto-oncogene, serine/threonine kinase
505	9606	Homo sapiens	MIR20B	MIRN20B, hsa-mir-20b, mir-20b	microRNA 20b

506	9606	Homo sapiens	PROCR	CCCA, CCD41, EPCR	protein C receptor
507	9606	Homo sapiens	GDNF	ATF, ATF1, ATF2, HFB1-GDNF, HSCR3	glial cell derived neurotrophic factor
508	9606	Homo sapiens	CDKN1C	BWCR, BWS, KIP2, WBS, p57, p57Kip2	cyclin dependent kinase inhibitor 1C
509	9606	Homo sapiens	TRPM7	ALSPDC, CHAK, CHAK1, LTRPC7, LTrpC-7, TRP-PLIK	transient receptor potential cation channel subfamily M member 7
510	9606	Homo sapiens	SLC6A4	5-HTT, 5-HTTLPR, 5HTT, HTT, OCD1, SERT, SERT1, hSERT	solute carrier family 6 member 4
511	9606	Homo sapiens	PRMT5	HRMT1L5, HSL7, IBP72, JBP1, SKB1, SKB1Hs	protein arginine methyltransferase 5
512	9606	Homo sapiens	VCAN	CSPG2, ERVR, GHAP, PG-M, WGN, WGN1	versican
513	9606	Homo sapiens	CD47	IAP, MER6, OA3	CD47 molecule
514	9606	Homo sapiens	CGA	CG-ALPHA, FSHA, GPA1, GPHA1, GPHa, HCG, LHA, TSHA	glycoprotein hormones, alpha polypeptide
515	9606	Homo sapiens	CD276	4Ig-B7-H3, B7-H3, B7H3, B7RP-2	CD276 molecule
516	9606	Homo sapiens	VTCN1	B7-H4, B7H4, B7S1, B7X, B7h.5, PRO1291, VCTN1	V-set domain containing T cell activation inhibitor 1
517	9606	Homo sapiens	EHMT2	BAT8, C6orf30, G9A, GAT8, KMT1C, NG36	euchromatic histone lysine methyltransferase 2
518	9606	Homo sapiens	FANCA	FA, FA-H, FA1, FAA, FACA, FAH, FANCH	FA complementation group A
519	9606	Homo sapiens	PTN	HARP, HB-GAM, HBBM, HBGF-8, HBGF8, HBNF, HBNF-1, NEGF1, OSF-1	pleiotrophin
520	9606	Homo sapiens	MACC1	7A5, SH3BP4L	MET transcriptional regulator MACC1
521	9606	Homo sapiens	EFEMP1	DHRD, DRAD, FBLN3, FBNL, FIBL-3, MLVT, MTLV, S1-5	EGF containing fibulin extracellular matrix protein 1
522	9606	Homo sapiens	DROSHA	ETOHI2, HSA242976, RANSE3L, RN3, RNASE3L, RNASEN	drosha ribonuclease III
523	9606	Homo sapiens	SCUBE2	CEGB1, CEGF1, CEGP1, scube/You	signal peptide, CUB domain and EGF like domain containing 2
524	9606	Homo sapiens	MIR26A1	MIR26A, MIRN26A1, mir-26a-1	microRNA 26a-1
525	9606	Homo sapiens	ACVR1	ACTRIA, ACVRLK2, ALK2, FOP, SKR1, TSRI, ACVR1	activin A receptor type 1
526	9606	Homo sapiens	ID2	GIG8A, ID2H, bHLHb26, ID2	inhibitor of DNA binding 2
527	9606	Homo sapiens	TLR2	CD282, TIL4	toll like receptor 2
528	9606	Homo sapiens	FOXA2	HNF3B, TCF3B	forkhead box A2

529	9606	Homo sapiens	KMT2D	AAD10, ALR, CAGL114, KABUK1, KMS, MLL2, MLL4, TNRC21	lysine methyltransferase 2D
530	9606	Homo sapiens	DRD2	D2DR, D2R	dopamine receptor D2
531	9606	Homo sapiens	NUMB	C14orf41, S171, c14_5527	NUMB endocytic adaptor protein
532	9606	Homo sapiens	MIR23A	MIRN23A, hsa-mir-23a, miRNA23A, mir-23a	microRNA 23a
533	9606	Homo sapiens	INPPL1	OPSMD, SHIP2	inositol polyphosphate phosphatase like 1
534	9606	Homo sapiens	ROBO1	DUTT1, SAX3	roundabout guidance receptor 1
535	9606	Homo sapiens	CARM1	PRMT4	coactivator associated arginine methyltransferase 1
536	9606	Homo sapiens	MIR106B	MIRN106B, mir-106b	microRNA 106b
537	9606	Homo sapiens	MIR29C	MIRN29C, miRNA29C, mir-29c	microRNA 29c
538	9606	Homo sapiens	VWF	F8VWF, VWD	von Willebrand factor
539	9606	Homo sapiens	KCNH1	EAG, EAG1, Kv10.1, TMBTS, ZLS1, h-eag, hEAG1	potassium voltage-gated channel subfamily H member 1
540	9606	Homo sapiens	ETS2	ETS2IT1	ETS proto-oncogene 2, transcription factor
541	9606	Homo sapiens	CSPG4	HMW-MAA, MCSP, MCSPG, MEL-CSPG, MSK16, NG2	chondroitin sulfate proteoglycan 4
542	9606	Homo sapiens	PRNP	ASCR, AltPrP, CD230, CJD, GSS, KURU, PRIP, PrP, PrP27-30, PrP33-35C, PrPc, p27-30	prion protein
543	9606	Homo sapiens	MBD2	DMTase, NY-CO-41	methyl-CpG binding domain protein 2
544	9606	Homo sapiens	PFKFB3	IPFK2, PFK2, iPFK-2	6-phosphofructo-2-kinase/fructose-2,6-biphosphatase 3
545	9606	Homo sapiens	SETDB1	ESET, H3-K9-HMTase4, KG1T, KMT1E, TDRD21	SET domain bifurcated histone lysine methyltransferase 1
546	9606	Homo sapiens	MECP2	AUTSX3, MRX16, MRX79, MRXS13, MRXSL, PPMX, RS, RTS, RTT	methyl-CpG binding protein 2
547	9606	Homo sapiens	BIK	BIP1, BP4, NBK	BCL2 interacting killer
548	9606	Homo sapiens	FABP7	B-FABP, BLBP, FABPB, MRG	fatty acid binding protein 7
549	9606	Homo sapiens	OPRM1	LMOR, M-OR-1, MOP, MOR, MOR1, OPRM	opioid receptor mu 1
550	9606	Homo sapiens	MIR99A	MIRN99A, mir-99a	microRNA 99a
551	9606	Homo sapiens	C3	AHUS5, ARMD9, ASPa, C3b, CPAMD1, HEL-S-62p, C3	complement C3
552	9606	Homo sapiens	MIR134	MIRN134, mir-134	microRNA 134

553	9606	Homo sapiens	SP7	OI11, OI12, OSX, osterix	Sp7 transcription factor
554	9606	Homo sapiens	PCNA	ATLD2	proliferating cell nuclear antigen
555	9606	Homo sapiens	GDF15	GDF-15, MIC-1, MIC1, NAG-1, PDF, PLAB, PTGFB	growth differentiation factor 15
556	9606	Homo sapiens	CLDN6		claudin 6
557	9606	Homo sapiens	TNFRSF1B	CD120b, TBPII, TNF-R-II, TNF-R75, TNFBR, TNFR1B, TNFR2, TNFR80, p75, p75TNFR	TNF receptor superfamily member 1B
558	9606	Homo sapiens	CAMP	CAP-18, CAP18, CRAMP, FALL-39, FALL39, HSD26, LL37	cathelicidin antimicrobial peptide
559	9606	Homo sapiens	SUMO1	DAP1, GMP1, OFC10, PIC1, SENP2, SMT3, SMT3C, SMT3H3, UBL1	small ubiquitin like modifier 1
560	9606	Homo sapiens	HDAC2	HD2, KDAC2, RPD3, YAF1	histone deacetylase 2
561	9606	Homo sapiens	PGF	D12S1900L, PIGF, PLGF, PlGF-2, SHGC-10760, PGF	placental growth factor
562	9606	Homo sapiens	MIR122	MIR122A, MIRN122, MIRN122A, hsa-mir-122, miRNA122, miRNA122A, mir-122	microRNA 122
563	9606	Homo sapiens	HAVCR2	CD366, HAVcr-2, KIM-3, SPTCL, TIM3, TIMD-3, TIMD3, Tim-3	hepatitis A virus cellular receptor 2
564	9606	Homo sapiens	DYRK2		dual specificity tyrosine phosphorylation regulated kinase 2
565	9606	Homo sapiens	MAP2K1	CFC3, MAPKK1, MEK1, MKK1, PRKMK1	mitogen-activated protein kinase kinase 1
566	9606	Homo sapiens	ORAI1	CRACM1, IMD9, ORAT1, TAM2, TMEM142A	ORAI calcium release-activated calcium modulator 1
567	9606	Homo sapiens	MIAT	C22orf35, GOMAFU, LINC00066, NCRNA00066, RNCR2, lncRNA-MIAT	myocardial infarction associated transcript
568	9606	Homo sapiens	BMPR2	BMPR-II, BMPR3, BMR2, BRK-3, POVD1, PPH1, T-ALK	bone morphogenetic protein receptor type 2
569	9606	Homo sapiens	LYN	JTK8, p53Lyn, p56Lyn	LYN proto-oncogene, Src family tyrosine kinase
570	9606	Homo sapiens	AGO2	CASC7, EIF2C2, LINC00980, PPD, Q10	argonaute RISC catalytic component 2
571	9606	Homo sapiens	THRB	C-ERBA-2, C-ERBA-BETA, ERBA2, GRTH, NR1A2, PRTH, THR11, THRB2, THRB	thyroid hormone receptor beta
572	9606	Homo sapiens	PRDM14	PFM11	PR/SET domain 14
573	9606	Homo sapiens	KITLG	DCUA, DFNA69, FPH2, FPHH, KL-1, Kitl, MGF,	KIT ligand

				SCF, SF, SHEP7, SLF	
574	9606	Homo sapiens	MIR223	MIRN223, miRNA223, mir-223	microRNA 223
575	9606	Homo sapiens	IQGAP1	HUMORFA01, SAR1, p195	IQ motif containing GTPase activating protein 1
576	9606	Homo sapiens	MDK	ARAP, MK, NEGF2	midkine
577	9606	Homo sapiens	TNFRSF10A	APO2, CD261, DR4, TRAILR-1, TRAILR1	TNF receptor superfamily member 10a
578	9606	Homo sapiens	TRIM28	KAP1, PPP1R157, RNF96, TF1B, TIF1B	tripartite motif containing 28
579	9606	Homo sapiens	TFAP2A	AP-2, AP-2alpha, AP2TF, BOFS, TFAP2	transcription factor AP-2 alpha
580	9606	Homo sapiens	SOD3	EC-SOD	superoxide dismutase 3
581	9606	Homo sapiens	ERN1	IRE1, IRE1P, IRE1a, hIRE1p	endoplasmic reticulum to nucleus signaling 1
582	9606	Homo sapiens	KLF6	BCD1, CBA1, COPEB, CPBP, GBF, PAC1, ST12, ZF9	Kruppel like factor 6
583	9606	Homo sapiens	COMP	EDM1, EPD1, MED, PSACH, THBS5, TSP5	cartilage oligomeric matrix protein
584	9606	Homo sapiens	HOXA10	HOX1, HOX1.8, HOX1H, PL	homeobox A10
585	9606	Homo sapiens	IGFBP7	AGM, FSTL2, IBP-7, IGFBP-7, IGFBP-7v, IGFBPRP1, MAC25, PSF, RAMSVPS, TAF	insulin like growth factor binding protein 7
586	9606	Homo sapiens	ELK1		ETS transcription factor ELK1
587	9606	Homo sapiens	FTH1	FHC, FTH, FTHL6, HFE5, PIG15, PLIF	ferritin heavy chain 1
588	9606	Homo sapiens	ALOX12	12-LOX, 12S-LOX, LOG12	arachidonate 12-lipoxygenase, 12S type
589	9606	Homo sapiens	CD200	MOX1, MOX2, MRC, OX-2	CD200 molecule
590	9606	Homo sapiens	MIR451A	MIR451, MIRN451, hsa-mir-451, hsa-mir-451a, mir-451a	microRNA 451a
591	9606	Homo sapiens	NEDD9	CAS-L, CAS2, CASL, CASS2, HEF1	neural precursor cell expressed, developmentally down-regulated 9
592	9606	Homo sapiens	MAP1LC3B	ATG8F, LC3B, MAP1A/1BLC3-a, MAP1LC3B	microtubule associated protein 1 light chain 3 beta
593	9606	Homo sapiens	MIR142	MIRN142, mir-142	microRNA 142
594	9606	Homo sapiens	RELB	I-REL, IMD53, IREL, REL-B	RELB proto-oncogene, NF-kB subunit
595	9606	Homo sapiens	TUG1	LINC00080, NCRNA00080, TI-227H	taurine up-regulated 1
596	9606	Homo sapiens	PTGER2	EP2	prostaglandin E receptor 2
597	9606	Homo sapiens	LOXL2	LOR, LOR2, WS9-14	lysyl oxidase like 2
598	9606	Homo sapiens	XIST	DXS1089, DXS399E, LINC00001, NCRNA00001, SXI1, swd66	X inactive specific transcript

599	9606	Homo sapiens	LIN28B	CSDD2	lin-28 homolog B
600	9606	Homo sapiens	CPT1A	CPT1, CPT1-L, L-CPT1	carnitine palmitoyltransferase 1A
601	9606	Homo sapiens	GFER	ALR, ERV1, HERV1, HPO, HPO1, HPO2, HSS	growth factor, augmenter of liver regeneration
602	9606	Homo sapiens	SATB2	GLSS	SATB homeobox 2
603	9606	Homo sapiens	HDAC8	CDA07, CDLS5, HD8, HDACL1, KDAC8, MRXS6, RPD3, WTS	histone deacetylase 8
604	9606	Homo sapiens	ADORA2B	ADORA2	adenosine A2b receptor
605	9606	Homo sapiens	PSMD10	dJ889N15.2, p28, p28(GANK)	proteasome 26S subunit, non-ATPase 10
606	9606	Homo sapiens	MSI1		musashi RNA binding protein 1
607	9606	Homo sapiens	ADORA3	A3AR	adenosine A3 receptor
608	9606	Homo sapiens	NTF3	HDNF, NGF-2, NGF2, NT-3, NT3	neurotrophin 3
609	9606	Homo sapiens	ROR1	NTRKR1, dJ537F10.1	receptor tyrosine kinase like orphan receptor 1
610	9606	Homo sapiens	SDCBP	MDA-9, MDA9, ST1, SYCL, TACIP18	syndecan binding protein
611	9606	Homo sapiens	MIR192	MIRN192, miR-192, miRNA192	microRNA 192
612	9606	Homo sapiens	SOD1	ALS, ALS1, HEL-S-44, IPOA, SOD, STAHP, hSod1, homodimer	superoxide dismutase 1
613	9606	Homo sapiens	SOX17	VUR3	SRY-box transcription factor 17
614	9606	Homo sapiens	PPARGC1B	ERRL1, PERC, PGC-1(beta), PGC1B	PPARG coactivator 1 beta
615	9606	Homo sapiens	RNF8	hRNF8	ring finger protein 8
616	9606	Homo sapiens	CDT1	DUP, RIS2	chromatin licensing and DNA replication factor 1
617	9606	Homo sapiens	PIWIL1	CT80.1, HIWI, MIWI, PIWI	piwi like RNA-mediated gene silencing 1
618	9606	Homo sapiens	MIR302A	MIRN302, MIRN302A, hsa-mir-302, mir-302a	microRNA 302a
619	9606	Homo sapiens	ZFX	ZNF926	zinc finger protein X-linked
620	9606	Homo sapiens	MIR191	MIRN191, miR-191	microRNA 191
621	9606	Homo sapiens	IL1RN	DIRA, ICIL-1RA, IL-1RN, IL-1ra, IL-1ra3, IL1F3, IL1RA, IRAP, MVCD4	interleukin 1 receptor antagonist
622	9606	Homo sapiens	ITGA9	ALPHA-RLC, ITGA4L, RLC	integrin subunit alpha 9
623	9606	Homo sapiens	NPM1	B23, NPM	nucleophosmin 1
624	9606	Homo sapiens	NLRP3	AGTAVPRL, AII, AVP, C1orf7, CIAS1, CLR1.1, DFNA34, FCAS, FCAS1, FCU, KEFH, MWS,	NLR family pyrin domain containing 3

				NALP3, PYPAF1	
625	9606	Homo sapiens	SEMA3C	SEMAE, SemE	semaphorin 3C
626	9606	Homo sapiens	TNFRSF1A	CD120a, FPF, TBP1, TNF-R, TNF-R-I, TNF-R55, TNFAR, TNFR1, TNFR55, TNFR60, p55, p55-R, p60	TNF receptor superfamily member 1A
627	9606	Homo sapiens	SOX1		SRY-box transcription factor 1
628	9606	Homo sapiens	LMO4		LIM domain only 4
629	9606	Homo sapiens	NR2E1	TLL, TLX, XTLL	nuclear receptor subfamily 2 group E member 1
630	9606	Homo sapiens	GNAS	AHO, C20orf451, GPSA, GSA, GSP, NESP, PITA3, POH, SCG6, SgVI, GNAS	GNAS complex locus
631	9606	Homo sapiens	CD40LG	CD154, CD40L, HIGM1, IGM, IMD3, T-BAM, TNFSF5, TRAP, gp39, hCD40L	CD40 ligand
632	9606	Homo sapiens	MIR608	MIRN608, hsa-mir-608	microRNA 608
633	9606	Homo sapiens	CORO1C	HCRNN4	coronin 1C
634	9606	Homo sapiens	IL2RA	CD25, IDDM10, IL2R, IMD41, TCGFR, p55	interleukin 2 receptor subunit alpha
635	9606	Homo sapiens	P2RX7	P2X7	purinergic receptor P2X 7
636	9606	Homo sapiens	CALR	CRT, HEL-S-99n, RO, SSA, cC1qR	calreticulin
637	9606	Homo sapiens	IL15	IL-15	interleukin 15
638	9606	Homo sapiens	PRKACA	PKACA, PPNAD4	protein kinase cAMP-activated catalytic subunit alpha
639	9606	Homo sapiens	ELK3	ERP, NET, SAP-2, SAP2	ETS transcription factor ELK3
640	9606	Homo sapiens	ADM	AM, PAMP	adrenomedullin
641	9606	Homo sapiens	FYN	SLK, SYN, p59-FYN	FYN proto-oncogene, Src family tyrosine kinase
642	9606	Homo sapiens	TSC2	LAM, PPP1R160, TSC4	TSC complex subunit 2
643	9606	Homo sapiens	TXN	TRDX, TRX, TRX1	thioredoxin
644	9606	Homo sapiens	CDX2	CDX-3/AS, CDX3, CDX2	caudal type homeobox 2
645	9606	Homo sapiens	HSPA8	HEL-33, HEL-S-72p, HSC54, HSC70, HSC71, HSP71, HSP73, HSPA10, LAP-1, LAP1, NIP71	heat shock protein family A (Hsp70) member 8
646	9606	Homo sapiens	NF2	ACN, BANF, SCH	neurofibromin 2
647	9606	Homo sapiens	ADAR	ADAR1, AGS6, DRADA, DSH, DSRAD, G1P1, IFI-4, IFI4, K88DSRBP, P136	adenosine deaminase RNA specific

648	9606	Homo sapiens	ABCC2	ABC30, CMOAT, DJS, MRP2, cMRP	ATP binding cassette subfamily C member 2
649	9606	Homo sapiens	NCL	C23, Nsr1	nucleolin
650	9606	Homo sapiens	GZMB	C11, CCPI, CGL-1, CGL1, CSP-B, CSPB, CTLA1, CTSGL1, HLP, SECT	granzyme B
651	9606	Homo sapiens	PTCH1	BCNS, NBCCS, PTC, PTC1, PTCH	patched 1
652	9606	Homo sapiens	IL7	IL-7	interleukin 7
653	9606	Homo sapiens	CXCR3	CD182, CD183, CKR-L2, CMKAR3, GPR9, IP10-R, Mig-R, MigR	C-X-C motif chemokine receptor 3
654	9606	Homo sapiens	SMAD7	CRCS3, MADH7, MADH8	SMAD family member 7
655	9606	Homo sapiens	CCL20	CKb4, Exodus, LARC, MIP-3-alpha, MIP-3a, MIP3A, SCYA20, ST38	C-C motif chemokine ligand 20
656	9606	Homo sapiens	MAP3K5	ASK1, MAPKKK5, MEKK5	mitogen-activated protein kinase kinase kinase 5
657	9606	Homo sapiens	KCNMA1	BKTM, CADEDS, IEG16, KCa1.1, MaxiK, PNKD3, SAKCA, SLO, SLO-ALPHA, SLO1, bA205K10.1, hSlo, mSLO1	potassium calcium-activated channel subfamily M alpha 1
658	9606	Homo sapiens	CD9	BTCC-1, DRAP-27, MIC3, MRP-1, TSPAN-29, TSPAN29	CD9 molecule
659	9606	Homo sapiens	VTN	V75, VN, VNT	vitronectin
660	9606	Homo sapiens	ATF4	CREB-2, CREB2, TAXREB67, TXREB	activating transcription factor 4
661	9606	Homo sapiens	HDAC4	AHO3, BDMR, HA6116, HD4, HDAC-4, HDAC-A, HDACA	histone deacetylase 4
662	9606	Homo sapiens	TSPO	BPBS, BZRP, DBI, IBP, MBR, PBR, PBS, PKBS, PTBR, mDRC, pk18	translocator protein
663	9606	Homo sapiens	CSK		C-terminal Src kinase
664	9606	Homo sapiens	GAS6	AXLLG, AXSF	growth arrest specific 6
665	9606	Homo sapiens	NFATC1	NF-ATC, NF-ATc1.2, NFAT2, NFATc	nuclear factor of activated T cells 1
666	9606	Homo sapiens	PADI4	PAD, PAD4, PADI5, PDI4, PDI5	peptidyl arginine deiminase 4
667	9606	Homo sapiens	DDX3X	CAP-Rf, DBX, DDX14, DDX3, HLP2, MRX102	DEAD-box helicase 3 X-linked
668	9606	Homo sapiens	FTL	LFTD, NBIA3	ferritin light chain
669	9606	Homo sapiens	BUB1B	BUB1beta, BUBR1, Bub1A, MAD3L, MVA1, SSK1, hBUBR1	BUB1 mitotic checkpoint serine/threonine kinase B
670	9606	Homo sapiens	UHRF1	ICBP90, Np95, RNF106, TDRD22, hNP95, hUHRF1, huNp95	ubiquitin like with PHD and ring finger domains 1

671	9606	Homo sapiens	DLK1	DLK, DLK-1, Delta1, FA1, PREF1, Pref-1, ZOG, pG2	delta like non-canonical Notch ligand 1
672	9606	Homo sapiens	APAF1	APAF-1, CED4	apoptotic peptidase activating factor 1
673	9606	Homo sapiens	AXIN1	AXIN, PPP1R49	axin 1
674	9606	Homo sapiens	CDC25C	CDC25, PPP1R60	cell division cycle 25C
675	9606	Homo sapiens	THRA	AR7, CHNG6, EAR7, ERB-T-1, ERBA, ERBA1, NR1A11, THRA2, c-ERBA-1, THRA	thyroid hormone receptor alpha
676	9606	Homo sapiens	FAP	DPPIVA, FAPalpha, SIMP, FAP	fibroblast activation protein alpha
677	9606	Homo sapiens	RAPGEF3	CAMP-GEFI, EPAC, EPAC1, HSU79275, bcm910	Rap guanine nucleotide exchange factor 3
678	9606	Homo sapiens	CXADR	CAR, CAR4/6, HCAR	CXADR Ig-like cell adhesion molecule
679	9606	Homo sapiens	DMBT1	GP340, SAG, SALSA, muclin	deleted in malignant brain tumors 1
680	9606	Homo sapiens	CD99	HBA71, MIC2, MIC2X, MIC2Y, MSK5X	CD99 molecule (Xg blood group)
681	9606	Homo sapiens	CD99	HBA71, MIC2, MIC2X, MIC2Y, MSK5X	CD99 molecule (Xg blood group)
682	9606	Homo sapiens	NUP98	ADIR2, NUP196, NUP96	nucleoporin 98
683	9606	Homo sapiens	LILRB1	CD85J, ILT-2, ILT2, LIR-1, LIR1, MIR-7, MIR7, PIR-B, PIRB	leukocyte immunoglobulin like receptor B1
684	9606	Homo sapiens	MIR181A1	MIR213, MIRN181A1, MIRN213, hsa-mir-181a-1, mir-181a-1, mir-213	microRNA 181a-1
685	9606	Homo sapiens	PTPN2	PTN2, PTPT, TC-PTP, TCELLPTP, TCPTP	protein tyrosine phosphatase non-receptor type 2
686	9606	Homo sapiens	RBL2	P130, Rb2	RB transcriptional corepressor like 2
687	9606	Homo sapiens	PRKAB1	AMPK, HAMPKb	protein kinase AMP-activated non-catalytic subunit beta 1
688	9606	Homo sapiens	TGFBR3	BGCAN, betaglycan	transforming growth factor beta receptor 3
689	9606	Homo sapiens	ARHGDIA	GDIA1, HEL-S-47e, NPHS8, RHOGDI, RHOGDI-1	Rho GDP dissociation inhibitor alpha
690	9606	Homo sapiens	MIR1-1	MIRN1-1, hsa-mir-1-1, miRNA1-1, mir-1-1	microRNA 1-1
691	9606	Homo sapiens	SOX11	CSS9, MRD27	SRY-box transcription factor 11
692	9606	Homo sapiens	DDB2	DDBB, UV-DDB2, XPE	damage specific DNA binding protein 2
693	9606	Homo sapiens	SFRP2	FRP-2, SARP1, SDF-5	secreted frizzled related protein 2
694	9606	Homo sapiens	KAT2A	GCN5, GCN5L2, PCAF-b, hGCN5	lysine acetyltransferase 2A
695	9606	Homo sapiens	FBLN1	FBLN, FIBL1	fibulin 1
696	9606	Homo sapiens	CCNA1	CT146	cyclin A1
697	9606	Homo sapiens	SENP1	SuPr-2	SUMO specific peptidase 1

698	9606	Homo sapiens	NOG	SYM1, SYNS1, SYNS1A	noggin
699	9606	Homo sapiens	SRSF3	SFRS3, SRp20	serine and arginine rich splicing factor 3
700	9606	Homo sapiens	TBXT	SAVA, T, TFT	T-box transcription factor T
701	9606	Homo sapiens	CTNNBIP1	ICAT	catenin beta interacting protein 1
702	9606	Homo sapiens	DLL1	DELTA1, DL1, Delta	delta like canonical Notch ligand 1
703	9606	Homo sapiens	MIR138-1	MIRN138-1, mir-138-1	microRNA 138-1
704	9606	Homo sapiens	MIR23B	MIRN23B, hsa-mir-23b, miRNA23B, mir-23b	microRNA 23b
705	9606	Homo sapiens	MIRLET7B	LET7B, MIRNLET7B, hsa-let-7b, let-7b	microRNA let-7b
706	9606	Homo sapiens	LIFR	CD118, LIF-R, SJS2, STWS, SWS	LIF receptor subunit alpha
707	9606	Homo sapiens	BHLHE40	BHLHB2, Clast5, DEC1, HLHB2, SHARP-2, SHARP2, STRA13, Stra14	basic helix-loop-helix family member e40
708	9606	Homo sapiens	ACLY	ACL, ATPCL, CLATP	ATP citrate lyase
709	9606	Homo sapiens	MIR106A	MIRN106A, mir-106, mir-106a	microRNA 106a
710	9606	Homo sapiens	CDH11	CAD11, CDHOB, ESWS, OB, OSF-4	cadherin 11
711	9606	Homo sapiens	LPAR1	EDG2, GPR26, Gpcr26, LPA1, Mrec1.3, VZG1, edg-2, rec.1.3, vzg-1	lysophosphatidic acid receptor 1
712	9606	Homo sapiens	CYTOR	C2orf59, LINC00152, NCRNA00152	cytoskeleton regulator RNA
713	9606	Homo sapiens	ISL1	ISLET1, Isl-1	ISL LIM homeobox 1
714	9606	Homo sapiens	MIR10A	MIRN10A, hsa-mir-10a, miRNA10A, mir-10a	microRNA 10a
715	9606	Homo sapiens	S1PR3	EDG-3, EDG3, LPB3, S1P3	sphingosine-1-phosphate receptor 3
716	9606	Homo sapiens	MIR128-1	MIR128A, MIRN128-1, MIRN128A, mir-128-1	microRNA 128-1
717	9606	Homo sapiens	SMAD6	AOVD2, HsT17432, MADH6, MADH7	SMAD family member 6
718	9606	Homo sapiens	ALDH1A3	ALDH1A6, ALDH6, MCOP8, RALDH3	aldehyde dehydrogenase 1 family member A3
719	9606	Homo sapiens	HOXA5	HOX1, HOX1.3, HOX1C	homeobox A5
720	9606	Homo sapiens	OLFM4	GC1, GW112, OLM4, OlfD, UNQ362, bA209J19.1, hGC-1, hOLfD	olfactomedin 4
721	9606	Homo sapiens	MSI2	MSI2H	musashi RNA binding protein 2
722	9606	Homo sapiens	MIR135B	MIRN135B, mir-135b	microRNA 135b
723	9606	Homo sapiens	HOXA11	HOX1, HOX1I, RUSAT1	homeobox A11
724	9606	Homo sapiens	HLA-B	AS, B-4901, HLAB	major histocompatibility complex, class I, B
725	9606	Homo sapiens	JMJD6	PSR, PTDSR, PTDSR1	jumonji domain containing 6, arginine demethylase and lysine hydroxylase

726	9606	Homo sapiens	SLC34A2	NAPI-3B, NAPI-IIb, NPTIIb, PULAM	solute carrier family 34 member 2
727	9606	Homo sapiens	MIR423	MIRN423, hsa-mir-423, mir-423	microRNA 423
728	9606	Homo sapiens	BRD7	BP75, CELTIX1, NAG4	bromodomain containing 7
729	9606	Homo sapiens	PIP5K1C	LCCS3, PIP5K-GAMMA, PIP5K1-gamma, PIP5Kgamma	phosphatidylinositol-4-phosphate 5-kinase type 1 gamma
730	9606	Homo sapiens	KDM2A	CXXC8, FBL11, FBL7, FBXL11, JHDM1A, LILINA	lysine demethylase 2A
731	9606	Homo sapiens	MIR495	MIRN495, hsa-mir-495, mir-495	microRNA 495
732	9606	Homo sapiens	CD4	CD4mut	CD4 molecule
733	9606	Homo sapiens	IBSP	BNSP, BSP, BSP-II, SP-II	integrin binding sialoprotein
734	9606	Homo sapiens	NMI		N-myc and STAT interactor
735	9606	Homo sapiens	FXYD5	DYSAD, HSPC113, IWU1, KCT1, OIT2, PRO6241, RIC	FXYD domain containing ion transport regulator 5
736	9606	Homo sapiens	FBXO5	EMI1, FBX5, Fbxo31	F-box protein 5
737	9606	Homo sapiens	TET3	hCG_40738	tet methylcytosine dioxygenase 3
738	9606	Homo sapiens	MIR1246	MIRN1246, hsa-mir-1246	microRNA 1246
739	9606	Homo sapiens	APOA1	HPALP2, apo(a)	apolipoprotein A1
740	9606	Homo sapiens	NOD2	ACUG, BLAU, BLAUS, CARD15, CD, CLR16.3, IBD1, NLRC2B, PSORAS1, YAOS, NOD2	nucleotide binding oligomerization domain containing 2
741	9606	Homo sapiens	HBB	CD113t-C, ECYT6, beta-globin	hemoglobin subunit beta
742	9606	Homo sapiens	F8	AHF, DXS1253EB, F8C, FVIII, HEMA, F8	coagulation factor VIII
743	9606	Homo sapiens	MIRLET7D	LET7D, MIRNLET7D, hsa-let-7d, let-7d	microRNA let-7d
744	9606	Homo sapiens	VHL	HRCA1, RCA11, pVHL, VHL	von Hippel-Lindau tumor suppressor
745	9606	Homo sapiens	PDLIM1	CLIM1, CLP-36, CLP36, HEL-S-112, hCLIM1	PDZ and LIM domain 1
746	9606	Homo sapiens	FMR1	FMRP, FRAXA, POF, POF1	FMRP translational regulator 1
747	9606	Homo sapiens	HTT	HD, IT15, LOMARS	huntingtin
748	9606	Homo sapiens	LCOR	C10orf12, MLR2	ligand dependent nuclear receptor corepressor
749	9606	Homo sapiens	DPP4	ADABP, ADCP2, CD26, DPPIV, TP103	dipeptidyl peptidase 4
750	9606	Homo sapiens	MIR448	MIRN448, hsa-mir-448, miRNA448	microRNA 448
751	9606	Homo sapiens	CALCA	CALC1, CGRP, CGRP-I, CGRP1, CT, KC, PCT	calcitonin related polypeptide alpha
752	9606	Homo sapiens	TNFSF13B	BAFF, BLYS, CD257, DTL, TALL-1, TALL1, THANK, TNFSF20, TNLG7A, ZTNF4	TNF superfamily member 13b
753	9606	Homo sapiens	SELP	CD62, CD62P, GMP140, GRMP, LECAM3,	selectin P

				PADGEM, PSEL	
754	9606	Homo sapiens	TNFAIP3	A20, AISBL, OTUD7C, TNFA1P2	TNF alpha induced protein 3
755	9606	Homo sapiens	CBS	CBSL, HIP4	cystathionine beta-synthase
756	9606	Homo sapiens	POLG	MDP1, MIRAS, MTDPS4A, MTDPS4B, PEO1, POLGA, SANDO, SCAE, POLG	DNA polymerase gamma, catalytic subunit
757	9606	Homo sapiens	CYBB	AMCBX2, CGD, GP91-1, GP91-PHOX, GP91PHOX, IMD34, NOX2, p91-PHOX	cytochrome b-245 beta chain
758	9606	Homo sapiens	TFRC	CD71, IMD46, T9, TFR, TFR1, TR, TRFR, p90	transferrin receptor
759	9606	Homo sapiens	IKBKG	AMCBX1, EDAID1, FIP-3, FIP3, Fip3p, IKK-gamma, IKKAP1, IKKG, IMD33, IP, IP1, IP2, IPD2, NEMO, ZC2HC9	inhibitor of nuclear factor kappa B kinase regulatory subunit gamma
760	9606	Homo sapiens	CHUK	IKBKA, IKK-alpha, IKK1, IKKA, NFKBIKA, TCF16	component of inhibitor of nuclear factor kappa B kinase complex
761	9606	Homo sapiens	BTK	AGMX1, AT, ATK, BPK, IGHD3, IMD1, PSCTK1, XLA	Bruton tyrosine kinase
762	9606	Homo sapiens	ELANE	ELA2, GE, HLE, HNE, NE, PMN-E, SCN1	elastase, neutrophil expressed
763	9606	Homo sapiens	COL3A1	EDS4A, EDSVASC, PMGEDSV	collagen type III alpha 1 chain
764	9606	Homo sapiens	NR1H4	BAR, FXR, HRR-1, HRR1, PFIC5, RIP14	nuclear receptor subfamily 1 group H member 4
765	9606	Homo sapiens	MAP3K7	CSCF, FMD2, MEKK7, TAK1, TGF1a	mitogen-activated protein kinase kinase kinase 7
766	9606	Homo sapiens	TEK	CD202B, GLC3E, TIE-2, TIE2, VMCM, VMCM1	TEK receptor tyrosine kinase
767	9606	Homo sapiens	IL6ST	CD130, CDW130, GP130, HIES4, IL-6RB, sGP130	interleukin 6 signal transducer
768	9606	Homo sapiens	C5	C5Da, C5b, CPAMD4, ECLZB, C5	complement C5
769	9606	Homo sapiens	CCL3	G0S19-1, LD78ALPHA, MIP-1-alpha, MIP1A, SCYA3	C-C motif chemokine ligand 3
770	9606	Homo sapiens	CTSL	CATL1, MEP, CTSL	cathepsin L
771	9606	Homo sapiens	TCF4	E2-2, FECD3, ITF-2, ITF2, PTHS, SEF-2, SEF2, SEF2-1, SEF2-1A, SEF2-1B, SEF2-1D, TCF-4, bHLHb19	transcription factor 4
772	9606	Homo sapiens	LIF	CDF, DIA, HILDA, MLPLI	LIF interleukin 6 family cytokine
773	9606	Homo sapiens	ACTA2	ACTSA	actin alpha 2, smooth muscle
774	9606	Homo sapiens	USP7	HAUSP, TEF1	ubiquitin specific peptidase 7

775	9606	Homo sapiens	KAT5	ESA1, HTATIP, HTATIP1, PLIP, TIP, TIP60, ZC2HC5, cPLA2	lysine acetyltransferase 5
776	9606	Homo sapiens	TNFSF13	APRIL, CD256, TALL-2, TALL2, TNLG7B, TRDL-1, UNQ383/PRO715, ZTNF2	TNF superfamily member 13
777	9606	Homo sapiens	S1PR1	CD363, CHEDG1, D1S3362, ECGF1, EDG-1, EDG1, S1P1	sphingosine-1-phosphate receptor 1
778	9606	Homo sapiens	ARRB1	ARB1, ARR1	arrestin beta 1
779	9606	Homo sapiens	BBC3	JFY-1, JFY1, PUMA	BCL2 binding component 3
780	9606	Homo sapiens	LRP6	ADCAD2, STHAG7	LDL receptor related protein 6
781	9606	Homo sapiens	LGALS9	HUATA, LGALS9	galectin 9
782	9606	Homo sapiens	PRDX2	HEL-S-2a, NKEF-B, NKEFB, PRP, PRX2, PRXII, PTX1, TDPX1, TPX1, TSA	peroxiredoxin 2
783	9606	Homo sapiens	NR4A2	HZF-3, NOT, NURR1, RNR1, TINUR	nuclear receptor subfamily 4 group A member 2
784	9606	Homo sapiens	HSP90AB1	D6S182, HSP84, HSP90B, HSPC2, HSPCB	heat shock protein 90 alpha family class B member 1
785	9606	Homo sapiens	CXCR1	C-C, C-C-CKR-1, CD128, CD181, CDw128a, CKR-1, CMKAR1, IL8R1, IL8RA, IL8RBA	C-X-C motif chemokine receptor 1
786	9606	Homo sapiens	MAPK9	JNK-55, JNK2, JNK2A, JNK2ALPHA, JNK2B, JNK2BETA, PRKM9, SAPK, SAPK1a, p54a, p54aSAPK	mitogen-activated protein kinase 9
787	9606	Homo sapiens	HK2	HKII, HXK2	hexokinase 2
788	9606	Homo sapiens	CD63	LAMP-3, ME491, MLA1, OMA81H, TSPAN30	CD63 molecule
789	9606	Homo sapiens	ANPEP	APN, CD13, GP150, LAP1, P150, PEPN	alanyl aminopeptidase, membrane
790	9606	Homo sapiens	BNIP3	NIP3	BCL2 interacting protein 3
791	9606	Homo sapiens	PDGFA	PDGF-A, PDGF1	platelet derived growth factor subunit A
792	9606	Homo sapiens	GLUD1	GDH, GDH1, GLUD	glutamate dehydrogenase 1
793	9606	Homo sapiens	DHX9	DDX9, LKP, NDH2, NDHII, RHA	DExH-box helicase 9
794	9606	Homo sapiens	EPHB2	BDPLT22, CAPB, DRT, EK5, EPHT3, ERK, Hek5, PCBC, Tyro5	EPH receptor B2
795	9606	Homo sapiens	WIF1	WIF-1	WNT inhibitory factor 1
796	9606	Homo sapiens	C1QA		complement C1q A chain
797	9606	Homo sapiens	E2F3	E2F-3	E2F transcription factor 3

798	9606	Homo sapiens	GREM1	C15DUPq, CKTSF1B1, CRAC1, CRCS4, DAND2, DRM, DUP15q, GREMLIN, HMPS, HMPS1, IHG-2, MPSH, PIG2	gremlin 1, DAN family BMP antagonist
799	9606	Homo sapiens	ULK1	ATG1, ATG1A, UNC51, Unc51.1, hATG1	unc-51 like autophagy activating kinase 1
800	9606	Homo sapiens	PROX1		prospero homeobox 1
801	9606	Homo sapiens	MAP3K14	FTDCR1B, HS, HSNIK, NIK	mitogen-activated protein kinase kinase kinase 14
802	9606	Homo sapiens	SMO	CRJS, FZD11, GxH, SMO	smoothened, frizzled class receptor
803	9606	Homo sapiens	HDAC5	HD5, NY-CO-9	histone deacetylase 5
804	9606	Homo sapiens	ADIPOR2	ACDCR2, PAQR2	adiponectin receptor 2
805	9606	Homo sapiens	CTAG1B	CT6.1, CTAG, CTAG1, ESO1, LAGE-2, LAGE2B, NY-ESO-1	cancer/testis antigen 1B
806	9606	Homo sapiens	HAX1	HCLSBP1, HS1BP1, SCN3	HCLS1 associated protein X-1
807	9606	Homo sapiens	RORC	IMD42, NR1F3, RORG, RZR-GAMMA, RZRG, TOR	RAR related orphan receptor C
808	9606	Homo sapiens	SKI	SGS, SKV	SKI proto-oncogene
809	9606	Homo sapiens	NOS1AP	6330408P19Rik, CAPON	nitric oxide synthase 1 adaptor protein
810	9606	Homo sapiens	LAMB3	AI1A, BM600-125KDA, LAM5, LAMNB1	laminin subunit beta 3
811	9606	Homo sapiens	MAP3K8	AURA2, COT, EST, ESTF, MEKK8, TPL2, Tpl-2, c-COT	mitogen-activated protein kinase kinase kinase 8
812	9606	Homo sapiens	CSNK1E	CKIe, CKIepsilon, HCKIE	casein kinase 1 epsilon
813	9606	Homo sapiens	ODC1	ODC	ornithine decarboxylase 1
814	9606	Homo sapiens	LAMA3	BM600, E170, LAMNA, LOCS	laminin subunit alpha 3
815	9606	Homo sapiens	E2F4	E2F-4	E2F transcription factor 4
816	9606	Homo sapiens	H2AZ1	H2A.Z-1, H2A.z, H2A/z, H2AFZ, H2AZ	H2A.Z variant histone 1
817	9606	Homo sapiens	RICTOR	AVO3, PIA, hAVO3	RPTOR independent companion of MTOR complex 2
818	9606	Homo sapiens	CDCP1	CD318, SIMA135, TRASK	CUB domain containing protein 1
819	9606	Homo sapiens	PRKAG2	AAKG, AAKG2, CMH6, H91620p, WPWS	protein kinase AMP-activated non-catalytic subunit gamma 2
820	9606	Homo sapiens	SLC2A3	GLUT3	solute carrier family 2 member 3
821	9606	Homo sapiens	CHD4	CHD-4, Mi-2b, Mi2-BETA, SIHIWES	chromodomain helicase DNA binding protein 4

822	9606	Homo sapiens	KDM6A	KABUK2, UTX, bA386N14.2	lysine demethylase 6A
823	9606	Homo sapiens	TAGLN	SM22, SM22-alpha, SMCC1, WS3-10, TAGLN	transgelin
824	9606	Homo sapiens	KDM6B	JMJD3, NEDCFSA	lysine demethylase 6B
825	9606	Homo sapiens	MIR133A1	MIRN133A1, mir-133a-1	microRNA 133a-1
826	9606	Homo sapiens	MSX2	CRS2, FPP, HOX8, MSH, PFM, PFM1	msh homeobox 2
827	9606	Homo sapiens	WNT4	SERKAL, WNT-4	Wnt family member 4
828	9606	Homo sapiens	LRP8	APOER2, HSZ75190, LRP-8, MCI1	LDL receptor related protein 8
829	9606	Homo sapiens	UBR5	DD5, EDD, EDD1, HYD	ubiquitin protein ligase E3 component n-recognin 5
830	9606	Homo sapiens	CEBPD	C/EBP-delta, CELF, CRP3, NF-IL6-beta	CCAAT enhancer binding protein delta
831	9606	Homo sapiens	CSH1	CS-1, CSA, CSMT, GHB3, PL, hCS-1, hCS-A	chorionic somatomammotropin hormone 1
832	9606	Homo sapiens	MIR196A1	MIRN196-1, MIRN196A1, mir-196a-1	microRNA 196a-1
833	9606	Homo sapiens	WNT2	INT1L1, IRP	Wnt family member 2
834	9606	Homo sapiens	MIR181B1	MIRN181B1, mir-181b-1	microRNA 181b-1
835	9606	Homo sapiens	MACROH2A1	H2A.y, H2A/y, H2AF12M, H2AFY.1, mH2A1, macroH2A1.2, MACROH2A1	macroH2A.1 histone
836	9606	Homo sapiens	MIR429	MIRN429, hsa-mir-429, mir-429	microRNA 429
837	9606	Homo sapiens	MYOCD	MYCD	myocardin
838	9606	Homo sapiens	PTPRZ1	HPTPZ, HPTPzeta, PTP-ZETA, PTP18, PTPRZ, PTPZ, R-PTP-zeta-2, RPTPB, RPTPbeta, phosphacan	protein tyrosine phosphatase receptor type Z1
839	9606	Homo sapiens	SDHC	CYB560, CYBL, PGL3, QPS1, SDH3	succinate dehydrogenase complex subunit C
840	9606	Homo sapiens	OLIG2	BHLHB1, OLIGO2, PRKCBP2, RACK17, bHLHe19	oligodendrocyte transcription factor 2
841	9606	Homo sapiens	TEAD1	AA, NTEF-1, REF1, TCF-13, TCF13, TEAD-1, TEF-1	TEA domain transcription factor 1
842	9606	Homo sapiens	EPHA4	EK8, HEK8, SEK, TYRO1	EPH receptor A4
843	9606	Homo sapiens	CD109	CPAMD7, p180, r150	CD109 molecule
844	9606	Homo sapiens	PFKM	ATP-PFK, GSD7, PFK-1, PFK-A, PFK1, PFKA, PFKX, PPP1R122	phosphofructokinase, muscle
845	9606	Homo sapiens	KDM5A	RBBP-2, RBBP2, RBP2	lysine demethylase 5A
846	9606	Homo sapiens	WWP2	AIP2, WWp2-like	WW domain containing E3 ubiquitin protein ligase 2

847	9606	Homo sapiens	SCO2	CEMCOX1, ECGF1, Gliostatin, MYP6, PD-ECGF, SCO1L, TP, TYMP, TdRPase	synthesis of cytochrome C oxidase 2
848	9606	Homo sapiens	PCLAF	KIAA0101, L5, NS5ATP9, OEATC, OEATC-1, OEATC1, PAF, PAF15, p15(PAF), p15/PAF, p15PAF	PCNA clamp associated factor
849	9606	Homo sapiens	GIT1		GIT ArfGAP 1
850	9606	Homo sapiens	BACE2	AEPLC, ALP56, ASP1, ASP21, BAE2, CDA13, CEAP1, DRAP	beta-secretase 2
851	9606	Homo sapiens	MIR486-1	MIR486, MIRN486, hsa-mir-486, hsa-mir-486-1, mir-486-1	microRNA 486-1
852	9606	Homo sapiens	TMPRSS4	CAPH2, MT-SP2, TMPRSS3	transmembrane serine protease 4
853	9606	Homo sapiens	MT-ND3	MTND3, ND3	mitochondrially encoded NADH dehydrogenase 3
854	9606	Homo sapiens	DANCR	AGU2, ANCR, KIAA0114, SNHG13, lncRNA-ANCR	differentiation antagonizing non-protein coding RNA
855	9606	Homo sapiens	MYBBP1A	P160, PAP2, Pol5	MYB binding protein 1a
856	9606	Homo sapiens	BIRC6	APOLLON, BRUCE	baculoviral IAP repeat containing 6
857	9606	Homo sapiens	ATF5	ATFX, HMFN0395	activating transcription factor 5
858	9606	Homo sapiens	WNT10B	SHFM6, STHAG8, WNT-12	Wnt family member 10B
859	9606	Homo sapiens	KDM2B	CXXC2, FBXL10, Fbl10, JHDM1B, PCCX2	lysine demethylase 2B
860	9606	Homo sapiens	METTL3	IME4, M6A, MT-A70, Spo8, hMETTL3	methyltransferase like 3
861	9606	Homo sapiens	PHLDA1	DT1P1B11, PHRIP, TDAG51	pleckstrin homology like domain family A member 1
862	9606	Homo sapiens	RBFOX2	FOX2, Fox-2, HNRBP2, HRNBP2, RBM9, RTA, dJ106I20.3, fxh	RNA binding fox-1 homolog 2
863	9606	Homo sapiens	NFIC	CTF, CTF5, NF-I, NFI	nuclear factor I C
864	9606	Homo sapiens	MIR16-2	MIRN16-2, mir-16-2, mir-16-3	microRNA 16-2
865	9606	Homo sapiens	SOX2-OT	NCRNA00043, SOX2OT	SOX2 overlapping transcript
866	9606	Homo sapiens	MIR30E	MIRN30E, mir-30e	microRNA 30e
867	9606	Homo sapiens	PUM2	PUMH2, PUML2	pumilio RNA binding family member 2
868	9606	Homo sapiens	CERK	LK4, dA59H18.2, dA59H18.3, hCERK	ceramide kinase
869	9606	Homo sapiens	MIR128-2	MIR128B, MIRN128-2, MIRN128B, mir-128-2, mir-128b	microRNA 128-2

870	9606	Homo sapiens	MAGEC1	CT7, CT7.1	MAGE family member C1
871	9606	Homo sapiens	ERAS	HRAS2, HRASP	ES cell expressed Ras
872	9606	Homo sapiens	MIR30C2	MIRN30C2, mir-30c-2	microRNA 30c-2
873	9606	Homo sapiens	POU5F1B	OCT4-PG1, OCT4PG1, OTF3C, OTF3P1, POU5F1P1, POU5F1P4, POU5FLC20, POU5FLC8	POU class 5 homeobox 1B
874	9606	Homo sapiens	TRIM59	IFT80L, MRF1, RNF104, TRIM57, TSBF1	tripartite motif containing 59
875	9606	Homo sapiens	MIR136	MIRN136, miRNA136, mir-136	microRNA 136
876	9606	Homo sapiens	MIR452	MIRN452, hsa-mir-452, mir-452	microRNA 452
877	9606	Homo sapiens	MIR302B	MIRN302B, mir-302b	microRNA 302b
878	9606	Homo sapiens	MIRLET7E	LET7E, MIRNLET7E, hsa-let-7e, let-7e	microRNA let-7e
879	9606	Homo sapiens	USP44		ubiquitin specific peptidase 44
880	9606	Homo sapiens	MIR379	MIRN379, hsa-mir-379, mir-379	microRNA 379
881	9606	Homo sapiens	CFTR	ABC35, ABCC7, CF/MRP, MRP7, TNR-CFTR, dJ760C5.1, CFTR	CF transmembrane conductance regulator
882	9606	Homo sapiens	IL17B	IL-17B, IL-20, NIRF, ZCYTO7	interleukin 17B
883	9606	Homo sapiens	RMST	LINC00054, NCRMS, NCRNA00054	rhabdomyosarcoma 2 associated transcript
884	9606	Homo sapiens	CYP2C9	CPC9, CYP2C, CYP2C10, CYPIIC9, P450IIC9	cytochrome P450 family 2 subfamily C member 9
885	9606	Homo sapiens	MIR301B	MIRN301B, mir-301b	microRNA 301b
886	9606	Homo sapiens	TARDBP	ALS10, TDP-43	TAR DNA binding protein
887	9606	Homo sapiens	IL13	IL-13, P600	interleukin 13
888	9606	Homo sapiens	HSPA4	APG-2, HEL-S-5a, HS24/P52, HSPH2, RY, hsp70, hsp70RY	heat shock protein family A (Hsp70) member 4
889	9606	Homo sapiens	IL12B	CLMF, CLMF2, IL-12B, IMD28, IMD29, NKSF, NKSF2	interleukin 12B
890	9606	Homo sapiens	S100B	NEF, S100, S100-B, S100beta	S100 calcium binding protein B
891	9606	Homo sapiens	G6PD	G6PD1	glucose-6-phosphate dehydrogenase
892	9606	Homo sapiens	ITGB2	CD18, LAD, LCAMB, LFA-1, MAC-1, MF17, MFI7	integrin subunit beta 2
893	9606	Homo sapiens	TUNAR	HI-LNC78, LINC00617, TUNA	TCL1 upstream neural differentiation-associated RNA
894	9606	Homo sapiens	TRAF6	MGC:3310, RNF85	TNF receptor associated factor 6
895	9606	Homo sapiens	UCP2	BMIQ4, SLC25A8, UCPH	uncoupling protein 2

896	9606	Homo sapiens	HSPD1	CPN60, GROEL, HLD4, HSP-60, HSP60, HSP65, HuCHA60, SPG13	heat shock protein family D (Hsp60) member 1
897	9606	Homo sapiens	IFNB1	IFB, IFF, IFN-beta, IFNB	interferon beta 1
898	9606	Homo sapiens	IL4R	CD124, IL-4RAA, IL4R	interleukin 4 receptor
899	9606	Homo sapiens	IL12A	CLMF, IL-12A, NFSK, NKSF1, P35	interleukin 12A
900	9606	Homo sapiens	COL1A2	EDSARTH2, EDSCV, OI4	collagen type I alpha 2 chain
901	9606	Homo sapiens	FGF21		fibroblast growth factor 21
902	9606	Homo sapiens	ALPL	AP-TNAP, APTNAP, HOPS, TNALP, TNAP, TNSALP	alkaline phosphatase, biomineralization associated
903	9606	Homo sapiens	APLN	APEL, XNPEP2	apelin
904	9606	Homo sapiens	A2M	A2MD, CPAMD5, FWP007, S863-7	alpha-2-macroglobulin
905	9606	Homo sapiens	MIR4319		microRNA 4319
906	9606	Homo sapiens	ITGA4	CD49D, IA4	integrin subunit alpha 4
907	9606	Homo sapiens	TBK1	FTDALS4, IIAE8, NAK, T2K	TANK binding kinase 1
908	9606	Homo sapiens	GATA2	DCML, IMD21, MONOMAC, NFE1B	GATA binding protein 2
909	9606	Homo sapiens	TAP1	ABC17, ABCB2, APT1, D6S114E, PSF-1, PSF1, RING4*0102N, TAP1N, TAP1	transporter 1, ATP binding cassette subfamily B member
910	9606	Homo sapiens	ANGPTL4	ARP4, FIAF, HARP, HFARP, NL2, PGAR, TGQTL, UNQ171, pp1158	angiopoietin like 4
911	9606	Homo sapiens	CSF3	C17orf33OS, GCSF, CSF3	colony stimulating factor 3
912	9606	Homo sapiens	TSC1	LAM, TSC	TSC complex subunit 1
913	9606	Homo sapiens	BAG3	BAG-3, BIS, CAIR-1, MFM6	BAG cochaperone 3
914	9606	Homo sapiens	NOD1	CARD4, CLR7.1, NLRC1	nucleotide binding oligomerization domain containing 1
915	9606	Homo sapiens	IRAK1	IRAK, pelle	interleukin 1 receptor associated kinase 1
916	9606	Homo sapiens	IL5	EDF, IL-5, TRF	interleukin 5
917	9606	Homo sapiens	ABCC4	MOAT-B, MOATB, MRP4	ATP binding cassette subfamily C member 4
918	9606	Homo sapiens	SF3B1	Hsh155, MDS, PRP10, PRPF10, SAP155, SF3b155	splicing factor 3b subunit 1
919	9606	Homo sapiens	ITGAL	CD11A, LFA-1, LFA1A	integrin subunit alpha L
920	9606	Homo sapiens	INHA		inhibin subunit alpha
921	9606	Homo sapiens	CCR6	BN-1, C-C CKR-6, CC-CKR-6, CCR-6, CD196, CKR-L3, CKRL3, CMKBR6, DCR2, DRY6,	C-C motif chemokine receptor 6

				GPR29, GPRCY4, STRL22	
922	9606	Homo sapiens	VCL	CMD1W, CMH15, HEL114, MV, MVCL	vinculin
923	9606	Homo sapiens	CXCL9	CMK, Humig, MIG, SCYB9, crg-10	C-X-C motif chemokine ligand 9
924	9606	Homo sapiens	MSX1	ECTD3, HOX7, HYD1, STHAG1	msh homeobox 1
925	9606	Homo sapiens	GPR55	LPIR1	G protein-coupled receptor 55
926	9606	Homo sapiens	CXCL5	ENA-78, SCYB5	C-X-C motif chemokine ligand 5
927	9606	Homo sapiens	REL	C-Rel	REL proto-oncogene, NF-kB subunit
928	9606	Homo sapiens	GATA6		GATA binding protein 6
929	9606	Homo sapiens	LAMC2	B2T, BM600, CSF, EBR2, EBR2A, LAMB2T, LAMNB2	laminin subunit gamma 2
930	9606	Homo sapiens	TUBA1A	B-ALPHA-1, LIS3, TUBA3	tubulin alpha 1a
931	9606	Homo sapiens	FPR1	FMLP, FPR	formyl peptide receptor 1
932	9606	Homo sapiens	CENPA	CENP-A, CenH3	centromere protein A
933	9606	Homo sapiens	TMSB4X	FX, PTMB4, TB4X, TMSB4	thymosin beta 4 X-linked
934	9606	Homo sapiens	MAPKAPK2	MAPKAP-K2, MK-2, MK2	MAPK activated protein kinase 2
935	9606	Homo sapiens	SERPINB2	HsT1201, PAI, PAI-2, PAI2, PLANH2	serpin family B member 2
936	9606	Homo sapiens	RAB27A	GS2, HsT18676, RAB27, RAM	RAB27A, member RAS oncogene family
937	9606	Homo sapiens	IGF2BP1	CRD-BP, CRDBP, IMP-1, IMP1, VICKZ1, ZBP1	insulin like growth factor 2 mRNA binding protein 1
938	9606	Homo sapiens	ROR2	BDB, BDB1, NTRKR2	receptor tyrosine kinase like orphan receptor 2
939	9606	Homo sapiens	PCBP1	HEL-S-85, HNRPE1, HNRPX, hnRNP-E1, hnRNP-X	poly(rC) binding protein 1
940	9606	Homo sapiens	ITGAX	CD11C, SLEB6	integrin subunit alpha X
941	9606	Homo sapiens	NTS	NMN-125, NN, NT, NT/N1, NTS	neurotensin
942	9606	Homo sapiens	PRDX3	AOP-1, AOP1, HBC189, MER5, PRO1748, SP-22, prx-III	peroxiredoxin 3
943	9606	Homo sapiens	HDGF	HMG1L2	heparin binding growth factor
944	9606	Homo sapiens	YES1	HsT441, P61-YES, Yes, c-yes	YES proto-oncogene 1, Src family tyrosine kinase
945	9606	Homo sapiens	MIR133B	MIRN133B, miRNA133B, mir-133b	microRNA 133b
946	9606	Homo sapiens	CCN3	IBP-9, IGFBP-9, IGFBP9, NOV, NOVh	cellular communication network factor 3
947	9606	Homo sapiens	NEUROD1	BETA2, BHF-1, MODY6, NEUROD, bHLHa3	neuronal differentiation 1

948	9606	Homo sapiens	NNMT		nicotinamide N-methyltransferase
949	9606	Homo sapiens	CBFB	PEBP2B	core-binding factor subunit beta
950	9606	Homo sapiens	IGFBP4	BP-4, HT29-IGFBP, IBP4, IGFBP-4	insulin like growth factor binding protein 4
951	9606	Homo sapiens	RAD9A	RAD9	RAD9 checkpoint clamp component A
952	9606	Homo sapiens	LTBP1		latent transforming growth factor beta binding protein 1
953	9606	Homo sapiens	RNF2	BAP-1, BAP1, DING, HIPI3, RING1B, RING2	ring finger protein 2
954	9606	Homo sapiens	SETD2	HBP231, HIF-1, HIP-1, HSPC069, HYPB, KMT3A, LLS, SET2, p231HBP	SET domain containing 2, histone lysine methyltransferase
955	9606	Homo sapiens	RAC2	EN-7, Gx, HSPC022, p21-Rac2	Rac family small GTPase 2
956	9606	Homo sapiens	RBL1	CP107, PRB1, p107	RB transcriptional corepressor like 1
957	9606	Homo sapiens	CLDN3	C7orf1, CPE-R2, CPETR2, HRVP1, RVP1	claudin 3
958	9606	Homo sapiens	IL3RA	CD123, IL3RY, IL3RX, IL3RY, hIL-3Ra, IL3RA	interleukin 3 receptor subunit alpha
959	9606	Homo sapiens	IL3RA	CD123, IL3RY, IL3RX, IL3RY, hIL-3Ra, IL3RA	interleukin 3 receptor subunit alpha
960	9606	Homo sapiens	GABARAP	ATG8A-a, MM46, GABARAP	GABA type A receptor-associated protein
961	9606	Homo sapiens	EFNB1	CFND, CFNS, EFB1, EFL3, EPLG2, Elk-L, LERK2	ephrin B1
962	9606	Homo sapiens	GJA3	CTRCT14, CX46, CZP3	gap junction protein alpha 3
963	9606	Homo sapiens	HOXA1	BSAS, HOX1, HOX1F	homeobox A1
964	9606	Homo sapiens	LILRB2	CD85D, ILT-4, ILT4, LIR-2, LIR2, MIR-10, MIR10	leukocyte immunoglobulin like receptor B2
965	9606	Homo sapiens	EPHA3	EK4, ETK, ETK1, HEK, HEK4, TYRO4	EPH receptor A3
966	9606	Homo sapiens	MIR33A	MIR33, MIRN33, MIRN33A, hsa-mir-33, hsa-mir-33a, miR-33, miRNA33A, mir-33a	microRNA 33a
967	9606	Homo sapiens	MBNL1	EXP, MBNL	muscleblind like splicing regulator 1
968	9606	Homo sapiens	PTK7	CCK-4, CCK4	protein tyrosine kinase 7 (inactive)
969	9606	Homo sapiens	E2F2	E2F-2	E2F transcription factor 2
970	9606	Homo sapiens	BCAP31	6C6-AG, BAP31, CDM, DDCH, DXS1357E	B cell receptor associated protein 31
971	9606	Homo sapiens	HMGB2	HMG2	high mobility group box 2
972	9606	Homo sapiens	DGCR8	C22orf12, DGCRK6, Gy1, pasha	DGCR8 microprocessor complex subunit
973	9606	Homo sapiens	MIR130B	MIRN130B, mir-130b	microRNA 130b
974	9606	Homo sapiens	BMX	ETK, PSCTK2, PSCTK3	BMX non-receptor tyrosine kinase
975	9606	Homo sapiens	AGO1	EIF2C, EIF2C1, GERP95, Q99, hAgo1	argonaute RISC component 1
976	9606	Homo sapiens	PALLD	CGI-151, CGI151, MYN, PNCA1, SIH002	palladin, cytoskeletal associated protein

977	9606	Homo sapiens	PDK1		pyruvate dehydrogenase kinase 1
978	9606	Homo sapiens	SEMA3F	SEMA-IV, SEMA4, SEMAK	semaphorin 3F
979	9606	Homo sapiens	KLF9	BTEB, BTEB1	Kruppel like factor 9
980	9606	Homo sapiens	AHNAK	AHNAKRS, PM227	AHNAK nucleoprotein
981	9606	Homo sapiens	MAGED1	DLXIN-1, NRAGE	MAGE family member D1
982	9606	Homo sapiens	DEPTOR	DEP.6, DEPDC6	DEP domain containing MTOR interacting protein
983	9606	Homo sapiens	TCF12	CRS3, HEB, HTF4, HsT17266, TCF-12, bHLHb20, p64	transcription factor 12
984	9606	Homo sapiens	TWIST2	AMS, BBRSAY, DERMO1, FFDD3, SETLSS, bHLHa39	twist family bHLH transcription factor 2
985	9606	Homo sapiens	SOX5	L-SOX5, L-SOX5B, L-SOX5F, LAMSHF	SRY-box transcription factor 5
986	9606	Homo sapiens	WNT3	INT4, TETAMS	Wnt family member 3
987	9606	Homo sapiens	MAPK13	MAPK 13, MAPK-13, PRKM13, SAPK4, p38delta	mitogen-activated protein kinase 13
988	9606	Homo sapiens	ACVR2B	ACTRIIB, ActR-IIB, HTX4	activin A receptor type 2B
989	9606	Homo sapiens	CAVIN1	CAVIN, CGL4, FKSG13, PTRF, cavin-1	caveolae associated protein 1
990	9606	Homo sapiens	MIR181A2	MIRN181A, MIRN181A2, hsa-mir-181a-2, mir-181a-2	microRNA 181a-2
991	9606	Homo sapiens	PLK3	CNK, FNK, PLK-3, PRK	polo like kinase 3
992	9606	Homo sapiens	TNFRSF10D	CD264, DCR2, TRAIL-R4, TRAILR4, TRUNDD	TNF receptor superfamily member 10d
993	9606	Homo sapiens	PTPRK	R-PTP-kappa	protein tyrosine phosphatase receptor type K
994	9606	Homo sapiens	MIR30B	MIRN30B, mir-30b	microRNA 30b
995	9606	Homo sapiens	CD200R1	CD200R, HCRTR2, MOX2R, OX2R	CD200 receptor 1
996	9606	Homo sapiens	PPP1R9B	PPP1R6, PPP1R9, SPINO, Spn	protein phosphatase 1 regulatory subunit 9B
997	9606	Homo sapiens	ATAD3A	HAYOS	ATPase family AAA domain containing 3A
998	9606	Homo sapiens	ESRRB	DFNB35, ERR2, ERRb, ESRL2, NR3B2	estrogen related receptor beta
999	9606	Homo sapiens	SPRY1	hSPRY1	sprouty RTK signaling antagonist 1
1000	9606	Homo sapiens	FGF5	HBGF-5, Smag-82, TCMGLY	fibroblast growth factor 5
1001	9606	Homo sapiens	BTC		betacellulin
1002	9606	Homo sapiens	GDF11	BMP-11, BMP11	growth differentiation factor 11
1003	9606	Homo sapiens	FANCL	FAAP43, PHF9, POG	FA complementation group L
1004	9606	Homo sapiens	PRKD3	EPK2, PKC-NU, PKD3, PRKCN, nPKC-NU	protein kinase D3

1005	9606	Homo sapiens	FOXD3	AIS1, Genesis, HFH2, VAMAS2	forkhead box D3
1006	9606	Homo sapiens	MIR675	MIRN675, hsa-mir-675	microRNA 675
1007	9606	Homo sapiens	WNT11	HWNT11	Wnt family member 11
1008	9606	Homo sapiens	MIR216A	MIR216, MIRN216, MIRN216A, miRNA216, mir-216, mir-216a	microRNA 216a
1009	9606	Homo sapiens	GPRC5A	GPCR5A, PEIG-1, RAI3, RAIG1, TIG1	G protein-coupled receptor class C group 5 member A
1010	9606	Homo sapiens	GPSM2	CMCS, DFNB82, LGN, PINS	G protein signaling modulator 2
1011	9606	Homo sapiens	RSPO3	CRISTIN1, PWTSR, THSD2	R-spondin 3
1012	9606	Homo sapiens	CXCL3	CINC-2b, GRO3, GROg, MIP-2b, MIP2B, SCYB3	C-X-C motif chemokine ligand 3
1013	9606	Homo sapiens	ACBD3	GCP60, GOCAP1, GOLPH1, PAP7	acyl-CoA binding domain containing 3
1014	9606	Homo sapiens	WNT5B		Wnt family member 5B
1015	9606	Homo sapiens	MIR491	MIRN491, hsa-mir-491, mir-491	microRNA 491
1016	9606	Homo sapiens	MIR613	MIRN613, hsa-mir-613	microRNA 613
1017	9606	Homo sapiens	STEAP1	PRSS24, STEAP	STEAP family member 1
1018	9606	Homo sapiens	GAS7		growth arrest specific 7
1019	9606	Homo sapiens	L3MBTL1	H-L(3)MBT, L3MBTL, ZC2HC3, dJ138B7.3	L3MBTL histone methyl-lysine binding protein 1
1020	9606	Homo sapiens	MIR32	MIRN32, hsa-mir-32, miR-32, miRNA32	microRNA 32
1021	9606	Homo sapiens	DDX43	CT13, HAGE	DEAD-box helicase 43
1022	9606	Homo sapiens	CDR1-AS	CDR1NAT, CDR1as, ciRS-7	CDR1 antisense RNA
1023	9606	Homo sapiens	UBASH3B	STS-1, STS1, TULA-2, TULA2, p70	ubiquitin associated and SH3 domain containing B
1024	9606	Homo sapiens	ZNF423	Ebfaz, JBTS19, NPHP14, OAZ, Roaz, ZFP423, Zfp104, hOAZ	zinc finger protein 423
1025	9606	Homo sapiens	BRAT1	BAAT1, C7orf27, NEDCAS, RMFSL	BRCA1 associated ATM activator 1
1026	9606	Homo sapiens	NAP1L4	NAP1L4b, NAP2, NAP2L, hNAP2	nucleosome assembly protein 1 like 4
1027	9606	Homo sapiens	NKX6-1	NKX6.1, NKX6A	NK6 homeobox 1
1028	9606	Homo sapiens	CTDSP1	NIF3, NLI-IF, NLIIF, SCP1	CTD small phosphatase 1
1029	9606	Homo sapiens	MIR7-3	MIRN7-3, hsa-mir-7-3, mir-7-3	microRNA 7-3
1030	9606	Homo sapiens	MIR7-2	MIRN7-2, hsa-mir-7-2, mir-7-2	microRNA 7-2
1031	9606	Homo sapiens	PLP2	A4, A4LSB	proteolipid protein 2
1032	9606	Homo sapiens	GDF3	KFS3, MCOP7, MCOPCB6	growth differentiation factor 3

1033	9606	Homo sapiens	ARID3B	BDP, DRIL2	AT-rich interaction domain 3B
1034	9606	Homo sapiens	PCID2	F10	PCI domain containing 2
1035	9606	Homo sapiens	GPM6B	M6B	glycoprotein M6B
1036	9606	Homo sapiens	IQGAP3		IQ motif containing GTPase activating protein 3
1037	9606	Homo sapiens	MIR18B	MIRN18B, hsa-mir-18b, mir-18b	microRNA 18b
1038	9606	Homo sapiens	PHF20L1	CGI-72, TDRD20B, URLC1	PHD finger protein 20 like 1
1039	9606	Homo sapiens	FBXL14	Fbl14	F-box and leucine rich repeat protein 14
1040	9606	Homo sapiens	MIR519C	MIRN519C, mir-519c	microRNA 519c
1041	63221	Homo sapiens neanderthalensis	COX2		cytochrome c oxidase subunit II

Table S4. Hesperetin potential targets (HTs) in BCSCs

No	Gene Symbol
1	APOE
2	APOA1
3	INS
4	IGF1
5	IGFBP3
6	AR
7	LEP
8	ADIPOQ
9	ESR1
10	PRL
11	CYP19A1
12	CDKN1A
13	CCNE1
14	RB1
15	PCNA
16	TP53
17	CCND2
18	CDKN1B
19	CDKN1C
20	RBL2
21	CCND1
22	CTNNB1
23	NUMB
24	MYC
25	ADAM17
26	HES1
27	CDKN2A
28	E2F4
29	CDKN2B
30	RBL1
31	SMAD3
32	PRMT5
33	RXRA
34	CEBPB
35	TNF
36	CEBPA
37	PPARG
38	IL6
39	ADIPOR2
40	ADIPOR1
41	CPT1A
42	ANGPTL4
43	ITGAV

Table S5. GO enrichment of HTs

Term	PValue	Genes
**Biological Process**		
GO:0042127~regulation of cell proliferation	2.01E-19	CEBPA, IL6, E2F4, TNF, RXRA, PPARG, TP53, SMAD3, IGF1, RB1, CTNNB1, HES1, CDKN1C, CCND1, CDKN1A, CDKN2A, CDKN1B, CDKN2B, CCND2, INS, APOE, ADAM17, PRL, IGFBP3, MYC
GO:0019220~regulation of phosphate metabolic process	4.84E-17	IL6, TNF, RBL2, RBL1, SMAD3, IGF1, RB1, ADIPOQ, CDKN1C, CCND1, CDKN1A, CDKN2A, CDKN1B, APOA1, CDKN2B, CCND2, APOE, INS, ADAM17, IGFBP3
GO:0051174~regulation of phosphorus metabolic process	4.84E-17	IL6, TNF, RBL2, RBL1, SMAD3, IGF1, RB1, ADIPOQ, CDKN1C, CCND1, CDKN1A, CDKN2A, CDKN1B, APOA1, CDKN2B, CCND2, APOE, INS, ADAM17, IGFBP3
GO:0042325~regulation of phosphorylation	5.32E-16	IL6, TNF, RBL2, RBL1, IGF1, RB1, ADIPOQ, CDKN1C, CCND1, CDKN1A, CDKN2A, CDKN1B, APOA1, CDKN2B, CCND2, APOE, INS, ADAM17, IGFBP3
GO:0010033~response to organic substance	4.45E-15	IL6, AR, TNF, RXRA, PPARG, ADIPOR2, ESR1, SMAD3, ADIPOR1, ADIPOQ, CTNNB1, LEP, CCNE1, CCND1, CDKN1A, CDKN2B, CCND2, INS, APOE, ADAM17, MYC
GO:0032583~regulation of gene-specific transcription	4.66E-15	CEBPA, IL6, TNF, RXRA, PPARG, TP53, IGF1, SMAD3, CTNNB1, HES1, CDKN1C, CDKN1B, INS
GO:0010604~positive regulation of macromolecule metabolic process	8.21E-15	CEBPA, AR, IL6, TNF, CEBPB, RXRA, PPARG, TP53, ESR1, SMAD3, IGF1, RB1, ADIPOQ, CTNNB1, HES1, CCNE1, CCND1, CCND2, INS, APOE, ADAM17, MYC
GO:0009719~response to endogenous stimulus	2.53E-14	IL6, AR, TNF, RXRA, PPARG, ADIPOR2, ESR1, ADIPOR1, ADIPOQ, CTNNB1, LEP, CCNE1, CCND1, CDKN1A, CCND2, INS, ADAM17
GO:0051726~regulation of cell cycle	2.68E-14	TNF, TP53, SMAD3, IGF1, RB1, CDKN1C, CCND1, CDKN1A, CDKN2A, CDKN1B, CDKN2B, CCND2, INS, PRMT5, ADAM17, MYC
GO:0040008~regulation of growth	4.14E-14	PPARG, ADIPOR2, TP53, SMAD3, ADIPOR1, IGF1, RB1, LEP, CDKN1A, CDKN2A, CDKN1B, APOE, INS, ADAM17, IGFBP3, PRL
GO:0009725~response to hormone stimulus	1.21E-13	IL6, AR, TNF, RXRA, PPARG, ADIPOR2, ESR1, ADIPOR1, ADIPOQ, CTNNB1, LEP, CCNE1, CCND1, CDKN1A, CCND2, INS
GO:0051338~regulation of transferase activity	1.48E-13	TNF, RBL2, RBL1, PPARG, RB1, CDKN1C, CCND1, CDKN1A, CDKN2A, CDKN1B, APOA1, CDKN2B, CCND2, APOE, INS, ADAM17
GO:0001558~regulation of cell growth	4.08E-13	CDKN1A, CDKN2A, CDKN1B, INS, APOE, PPARG, TP53, ADIPOR2, SMAD3, ADAM17, ADIPOR1, RB1, IGFBP3
GO:0051329~interphase of mitotic cell cycle	5.04E-13	CDKN1C, CCNE1, CDKN1A, CCND1, CDKN1B, CDKN2A, E2F4, CDKN2B, CCND2, ADAM17, RB1
GO:0051325~interphase	6.76E-13	CDKN1C, CCNE1, CDKN1A, CCND1, CDKN1B, CDKN2A, E2F4, CDKN2B, CCND2, ADAM17, RB1

GO:0008361~regulation of cell size	8.35E-13	AR, CDKN1A, CDKN2A, E2F4, CDKN1B, INS, PPARG, TP53, ADIPOR2, SMAD3, ADAM17, ADIPOR1, RB1
GO:0010605~negative regulation of macromolecule metabolic process	1.40E-12	CEBPA, IL6, TNF, RXRA, RBL1, PPARG, TP53, SMAD3, RB1, ADIPOQ, CTNNB1, CDKN1C, HES1, CDKN2A, CDKN1B, INS, ITGAV, IGFBP3, MYC
GO:0008285~negative regulation of cell proliferation	1.94E-12	CEBPA, IL6, TNF, RXRA, PPARG, TP53, SMAD3, RB1, CDKN1C, CDKN1A, CDKN2A, CDKN1B, CDKN2B, APOE, IGFBP3
GO:0009890~negative regulation of biosynthetic process	5.22E-12	CEBPA, IL6, TNF, RXRA, RBL1, PPARG, TP53, SMAD3, RB1, ADIPOQ, CTNNB1, CDKN1C, HES1, CDKN1B, APOE, INS, ITGAV
GO:0000082~G1/S transition of mitotic cell cycle	5.42E-12	CCNE1, CDKN1A, CCND1, CDKN1B, CDKN2A, E2F4, CCND2, ADAM17, RB1
GO:0045935~positive regulation of nucleobase, nucleoside, nucleotide and nucleic acid metabolic process	1.89E-11	CEBPA, IL6, AR, TNF, CEBPB, RXRA, PPARG, TP53, SMAD3, IGF1, RB1, CTNNB1, HES1, CCNE1, APOE, INS, MYC
GO:0032535~regulation of cellular component size	2.12E-11	AR, CDKN1A, CDKN2A, E2F4, CDKN1B, INS, PPARG, TP53, ADIPOR2, SMAD3, ADAM17, ADIPOR1, RB1
GO:0007346~regulation of mitotic cell cycle	2.57E-11	CCND1, CDKN2A, TNF, CDKN2B, INS, PRMT5, TP53, SMAD3, IGF1, RB1, MYC
GO:0051094~positive regulation of developmental process	2.86E-11	LEP, CCNE1, IL6, TNF, CDKN2B, INS, PPARG, NUMB, SMAD3, RB1, IGFBP3, CTNNB1, ANGPTL4
GO:0051173~positive regulation of nitrogen compound metabolic process	3.03E-11	CEBPA, IL6, AR, TNF, CEBPB, RXRA, PPARG, TP53, SMAD3, IGF1, RB1, CTNNB1, HES1, CCNE1, APOE, INS, MYC
GO:0043549~regulation of kinase activity	3.10E-11	TNF, RBL2, RBL1, RB1, CDKN1C, CCND1, CDKN1A, CDKN2A, CDKN1B, CDKN2B, APOE, INS, CCND2, ADAM17
GO:0019216~regulation of lipid metabolic process	4.68E-11	LEP, TNF, APOA1, RBL2, INS, APOE, PPARG, RBL1, RB1, ADIPOQ
GO:0045944~positive regulation of transcription from RNA polymerase II promoter	5.01E-11	CEBPA, IL6, AR, CEBPB, TNF, RXRA, PPARG, TP53, SMAD3, IGF1, RB1, CTNNB1, HES1, MYC
GO:0042981~regulation of apoptosis	7.61E-11	IL6, TNF, CEBPB, RXRA, TP53, ESR1, SMAD3, IGF1, ADIPOQ, CDKN1A, CDKN2A, CDKN1B, INS, APOE, ADAM17, IGFBP3, MYC, ANGPTL4
GO:0031328~positive regulation of cellular biosynthetic process	7.62E-11	CEBPA, IL6, AR, TNF, CEBPB, RXRA, PPARG, TP53, SMAD3, IGF1, RB1, CTNNB1, HES1, CCNE1, APOE, INS, MYC
GO:0045893~positive regulation of transcription, DNA-dependent	8.12E-11	CEBPA, IL6, AR, CEBPB, TNF, RXRA, PPARG, TP53, SMAD3, IGF1, RB1, CTNNB1, HES1, CCNE1, MYC
GO:0010628~positive regulation of gene expression	8.78E-11	CEBPA, IL6, AR, CEBPB, TNF, RXRA, PPARG, TP53, ESR1, SMAD3, IGF1, RB1, CTNNB1, HES1, CCNE1, MYC
GO:0043067~regulation of programmed cell death	8.89E-11	IL6, TNF, CEBPB, RXRA, TP53, ESR1, SMAD3, IGF1, ADIPOQ, CDKN1A, CDKN2A, CDKN1B, INS, APOE, ADAM17, IGFBP3, MYC, ANGPTL4
GO:0051254~positive regulation of RNA metabolic process	9.07E-11	CEBPA, IL6, AR, CEBPB, TNF, RXRA, PPARG, TP53, SMAD3, IGF1, RB1, CTNNB1, HES1, CCNE1, MYC

GO:0010941~regulation of cell death	9.42E-11	IL6, TNF, CEBPB, RXRA, TP53, ESR1, SMAD3, IGF1, ADIPOQ, CDKN1A, CDKN2A, CDKN1B, INS, APOE, ADAM17, IGFBP3, MYC, ANGPTL4
GO:0009891~positive regulation of biosynthetic process	9.45E-11	CEBPA, IL6, AR, TNF, CEBPB, RXRA, PPARG, TP53, SMAD3, IGF1, RB1, CTNNB1, HES1, CCNE1, APOE, INS, MYC
GO:0006357~regulation of transcription from RNA polymerase II promoter	1.84E-10	CEBPA, IL6, AR, TNF, CEBPB, E2F4, RXRA, RBL1, PPARG, TP53, SMAD3, IGF1, RB1, CTNNB1, CDKN1C, HES1, MYC
GO:0043193~positive regulation of gene-specific transcription	2.08E-10	CEBPA, HES1, IL6, TNF, PPARG, TP53, SMAD3, IGF1, CTNNB1
GO:0048545~response to steroid hormone stimulus	2.60E-10	CCNE1, CDKN1A, CCND1, IL6, TNF, CCND2, RXRA, PPARG, ESR1, ADIPOQ, CTNNB1
GO:0000079~regulation of cyclin-dependent protein kinase activity	2.61E-10	CDKN1C, CDKN1A, CCND1, CDKN1B, CDKN2A, CDKN2B, CCND2, ADAM17
GO:0030308~negative regulation of cell growth	3.28E-10	CDKN1A, CDKN1B, CDKN2A, PPARG, TP53, ADIPOR2, SMAD3, ADIPOR1, RB1
GO:0010557~positive regulation of macromolecule biosynthetic process	4.59E-10	CEBPA, IL6, AR, CEBPB, TNF, RXRA, PPARG, TP53, SMAD3, IGF1, RB1, CTNNB1, HES1, CCNE1, INS, MYC
GO:0043066~negative regulation of apoptosis	4.67E-10	IL6, TNF, CEBPB, TP53, ESR1, IGF1, SMAD3, CDKN1A, APOE, INS, ADAM17, MYC, ANGPTL4
GO:0007049~cell cycle	4.82E-10	E2F4, RBL2, RBL1, TP53, SMAD3, RB1, CTNNB1, CDKN1C, CCNE1, CCND1, CDKN1A, CDKN2A, CDKN1B, CDKN2B, CCND2, ADAM17, MYC
GO:0010558~negative regulation of macromolecule biosynthetic process	4.92E-10	CDKN1C, CEBPA, HES1, IL6, TNF, CDKN1B, ITGAV, RXRA, PPARG, RBL1, TP53, SMAD3, RB1, ADIPOQ, CTNNB1
GO:0043069~negative regulation of programmed cell death	5.49E-10	IL6, TNF, CEBPB, TP53, ESR1, IGF1, SMAD3, CDKN1A, APOE, INS, ADAM17, MYC, ANGPTL4
GO:0060548~negative regulation of cell death	5.66E-10	IL6, TNF, CEBPB, TP53, ESR1, IGF1, SMAD3, CDKN1A, APOE, INS, ADAM17, MYC, ANGPTL4
GO:0045792~negative regulation of cell size	5.94E-10	CDKN1A, CDKN1B, CDKN2A, PPARG, TP53, ADIPOR2, SMAD3, ADIPOR1, RB1
GO:0031327~negative regulation of cellular biosynthetic process	6.85E-10	CDKN1C, CEBPA, HES1, IL6, TNF, CDKN1B, INS, RXRA, PPARG, RBL1, TP53, SMAD3, RB1, ADIPOQ, CTNNB1
GO:0045941~positive regulation of transcription	7.34E-10	CEBPA, IL6, AR, CEBPB, TNF, RXRA, PPARG, TP53, SMAD3, IGF1, RB1, CTNNB1, HES1, CCNE1, MYC
GO:0022402~cell cycle process	7.51E-10	E2F4, TP53, SMAD3, RB1, CTNNB1, CDKN1C, CCNE1, CCND1, CDKN1A, CDKN2A, CDKN1B, CDKN2B, CCND2, ADAM17, MYC
GO:0007050~cell cycle arrest	8.17E-10	CDKN1C, CDKN1A, CDKN1B, CDKN2A, CDKN2B, TP53, SMAD3, RB1, MYC
GO:0009967~positive regulation of signal transduction	1.03E-09	LEP, CDKN1C, IL6, TNF, CDKN2B, INS, ESR1, ADAM17, IGF1, PRL, ADIPOQ, CTNNB1

GO:0045926~negative regulation of growth	1.39E-09	CDKN1A, CDKN1B, CDKN2A, PPARG, TP53, ADIPOR2, SMAD3, ADIPOR1, RB1
GO:0045597~positive regulation of cell differentiation	1.45E-09	LEP, CCNE1, IL6, CDKN2B, INS, NUMB, PPARG, SMAD3, RB1, IGFBP3, CTNNB1
GO:0001932~regulation of protein amino acid phosphorylation	2.30E-09	CCND1, IL6, TNF, APOA1, CCND2, INS, ADAM17, IGF1, IGFBP3, ADIPOQ
GO:0010647~positive regulation of cell communication	3.25E-09	LEP, CDKN1C, IL6, TNF, CDKN2B, INS, ESR1, ADAM17, IGF1, PRL, ADIPOQ, CTNNB1
GO:0045859~regulation of protein kinase activity	5.34E-09	CDKN1C, CDKN1A, CCND1, CDKN1B, CDKN2A, TNF, CDKN2B, CCND2, INS, APOE, ADAM17, RB1
GO:0000122~negative regulation of transcription from RNA polymerase II promoter	6.17E-09	CDKN1C, CEBPA, HES1, TNF, RXRA, PPARG, RBL1, TP53, SMAD3, RB1, CTNNB1
GO:0045892~negative regulation of transcription, DNA-dependent	7.41E-09	CDKN1C, CEBPA, HES1, CDKN1B, TNF, RXRA, PPARG, RBL1, TP53, SMAD3, RB1, CTNNB1
GO:0051253~negative regulation of RNA metabolic process	8.81E-09	CDKN1C, CEBPA, HES1, CDKN1B, TNF, RXRA, PPARG, RBL1, TP53, SMAD3, RB1, CTNNB1
GO:0006355~regulation of transcription, DNA-dependent	8.93E-09	CEBPA, AR, IL6, TNF, CEBPB, E2F4, RXRA, PPARG, RBL1, TP53, ESR1, SMAD3, IGF1, RB1, CTNNB1, CDKN1C, HES1, CCNE1, CDKN2A, CDKN1B, INS, MYC
GO:0043086~negative regulation of catalytic activity	9.10E-09	CDKN1C, CDKN1A, CDKN1B, CDKN2A, APOA1, INS, APOE, PPARG, TP53, RB1, ANGPTL4
GO:0001934~positive regulation of protein amino acid phosphorylation	9.39E-09	CCND1, IL6, TNF, CCND2, INS, ADAM17, IGF1, ADIPOQ
GO:0032268~regulation of cellular protein metabolic process	1.27E-08	IL6, TNF, IGF1, ADIPOQ, CCND1, CDKN2A, APOA1, APOE, INS, CCND2, ITGAV, ADAM17, IGFBP3
GO:0001889~liver development	1.27E-08	CEBPA, HES1, CCNE1, CCND1, CCND2, RXRA, CTNNB1
GO:0051252~regulation of RNA metabolic process	1.34E-08	CEBPA, AR, IL6, TNF, CEBPB, E2F4, RXRA, PPARG, RBL1, TP53, ESR1, SMAD3, IGF1, RB1, CTNNB1, CDKN1C, HES1, CCNE1, CDKN2A, CDKN1B, INS, MYC
GO:0051348~negative regulation of transferase activity	1.60E-08	CDKN1C, CDKN1A, CDKN1B, CDKN2A, INS, APOE, PPARG, RB1
GO:0031399~regulation of protein modification process	1.66E-08	CCND1, IL6, CDKN2A, TNF, APOA1, CCND2, INS, ADAM17, IGF1, IGFBP3, ADIPOQ
GO:0042327~positive regulation of phosphorylation	1.72E-08	CCND1, IL6, TNF, CCND2, INS, ADAM17, IGF1, ADIPOQ
GO:0009991~response to extracellular stimulus	1.89E-08	LEP, CCNE1, CDKN1A, CCND1, CDKN2B, RXRA, PPARG, TP53, ADIPOR2, ADIPOQ
GO:0045937~positive regulation of phosphate metabolic process	2.13E-08	CCND1, IL6, TNF, CCND2, INS, ADAM17, IGF1, ADIPOQ
GO:0010562~positive regulation of phosphorus metabolic process	2.13E-08	CCND1, IL6, TNF, CCND2, INS, ADAM17, IGF1, ADIPOQ

GO:0010629~negative regulation of gene expression	2.50E-08	CDKN1C, CEBPA, HES1, TNF, CDKN1B, RXRA, PPARG, RBL1, TP53, SMAD3, RB1, MYC, CTNNB1
GO:0008284~positive regulation of cell proliferation	3.52E-08	HES1, CDKN1A, CCND1, IL6, CDKN1B, TNF, CCND2, INS, ADAM17, IGF1, PRL, MYC
GO:0043065~positive regulation of apoptosis	5.18E-08	CDKN1A, CDKN1B, CDKN2A, CEBPB, TNF, APOE, RXRA, TP53, SMAD3, IGFBP3, MYC, ADIPOQ
GO:0044092~negative regulation of molecular function	5.37E-08	CDKN1C, CDKN1A, CDKN1B, CDKN2A, APOA1, INS, APOE, PPARG, TP53, RB1, ANGPTL4
GO:0043068~positive regulation of programmed cell death	5.56E-08	CDKN1A, CDKN1B, CDKN2A, CEBPB, TNF, APOE, RXRA, TP53, SMAD3, IGFBP3, MYC, ADIPOQ
GO:0010942~positive regulation of cell death	5.83E-08	CDKN1A, CDKN1B, CDKN2A, CEBPB, TNF, APOE, RXRA, TP53, SMAD3, IGFBP3, MYC, ADIPOQ
GO:0007242~intracellular signaling cascade	6.83E-08	AR, TNF, RXRA, ADIPOR2, TP53, ESR1, ADIPOR1, IGF1, RB1, CTNNB1, LEP, CCNE1, CCND1, APOA1, APOE, INS, PCNA, PRL
GO:0001817~regulation of cytokine production	7.04E-08	IL6, CEBPB, TNF, APOA1, INS, PPARG, ADAM17, SMAD3, ADIPOQ
GO:0050678~regulation of epithelial cell proliferation	7.62E-08	CDKN1C, CDKN1B, CDKN2B, CCND2, SMAD3, IGF1, PRL
GO:0014070~response to organic cyclic substance	8.00E-08	CCNE1, CDKN1A, CCND1, CDKN2B, CCND2, RXRA, PPARG, CTNNB1
GO:0016481~negative regulation of transcription	1.01E-07	CDKN1C, CEBPA, HES1, CDKN1B, TNF, RXRA, PPARG, RBL1, TP53, SMAD3, RB1, CTNNB1
GO:0051384~response to glucocorticoid stimulus	1.34E-07	CCNE1, CDKN1A, CCND1, IL6, TNF, RXRA, ADIPOQ
GO:0031667~response to nutrient levels	1.35E-07	LEP, CCNE1, CCND1, CDKN2B, RXRA, PPARG, TP53, ADIPOR2, ADIPOQ
GO:0000278~mitotic cell cycle	1.40E-07	CDKN1C, CCNE1, CDKN1A, CCND1, CDKN1B, CDKN2A, E2F4, CDKN2B, CCND2, ADAM17, RB1
GO:0060284~regulation of cell development	1.84E-07	HES1, CCND2, INS, APOE, NUMB, PPARG, TP53, SMAD3, IGFBP3
GO:0031960~response to corticosteroid stimulus	2.25E-07	CCNE1, CDKN1A, CCND1, IL6, TNF, RXRA, ADIPOQ
GO:0042326~negative regulation of phosphorylation	2.51E-07	CDKN1C, CDKN1A, CDKN1B, CDKN2A, CDKN2B, IGFBP3
GO:0045596~negative regulation of cell differentiation	2.75E-07	HES1, CCND1, ITGAV, PPARG, TP53, SMAD3, IGF1, ADIPOQ, CTNNB1
GO:0045834~positive regulation of lipid metabolic process	2.81E-07	TNF, APOA1, INS, APOE, PPARG, ADIPOQ
GO:0045934~negative regulation of nucleobase, nucleoside, nucleotide and nucleic acid metabolic process	3.02E-07	CDKN1C, CEBPA, HES1, CDKN1B, TNF, RXRA, PPARG, RBL1, TP53, SMAD3, RB1, CTNNB1
GO:0045449~regulation of transcription	3.05E-07	CEBPA, AR, IL6, TNF, CEBPB, E2F4, RBL2, RXRA, PPARG, RBL1, TP53, ESR1, SMAD3, IGF1, RB1, CTNNB1, CDKN1C, HES1, CCNE1, CDKN2A, CDKN1B, INS, PRMT5, MYC
GO:0033673~negative regulation of kinase activity	3.17E-07	CDKN1C, CDKN1A, CDKN1B, CDKN2A, INS, APOE, RB1

GO:0051172~negative regulation of nitrogen compound metabolic process	3.46E-07	CDKN1C, CEBPA, HES1, CDKN1B, TNF, RXRA, PPARG, RBL1, TP53, SMAD3, RB1, CTNNB1
GO:0010563~negative regulation of phosphorus metabolic process	3.50E-07	CDKN1C, CDKN1A, CDKN1B, CDKN2A, CDKN2B, IGFBP3
GO:0032582~negative regulation of gene-specific transcription	3.50E-07	CDKN1C, HES1, CDKN1B, RXRA, PPARG, SMAD3
GO:0045936~negative regulation of phosphate metabolic process	3.50E-07	CDKN1C, CDKN1A, CDKN1B, CDKN2A, CDKN2B, IGFBP3
GO:0022403~cell cycle phase	3.96E-07	CDKN1C, CCNE1, CDKN1A, CCND1, CDKN1B, CDKN2A, E2F4, CDKN2B, CCND2, ADAM17, RB1
GO:0010551~regulation of specific transcription from RNA polymerase II promoter	4.11E-07	CEBPA, HES1, RXRA, PPARG, TP53, SMAD3, CTNNB1
GO:0032270~positive regulation of cellular protein metabolic process	4.89E-07	CCND1, IL6, TNF, CCND2, INS, APOE, ADAM17, IGF1, ADIPOQ
GO:0032101~regulation of response to external stimulus	5.18E-07	LEP, IL6, INS, APOE, PPARG, ADAM17, SMAD3, ADIPOQ
GO:0008283~cell proliferation	6.37E-07	AR, APOA1, PRMT5, RXRA, NUMB, TP53, PCNA, IGF1, PRL, MYC, CTNNB1
GO:0051241~negative regulation of multicellular organismal process	6.39E-07	IL6, TNF, APOA1, INS, APOE, PPARG, ADAM17, ADIPOQ
GO:0051247~positive regulation of protein metabolic process	6.73E-07	CCND1, IL6, TNF, CCND2, INS, APOE, ADAM17, IGF1, ADIPOQ
GO:0010740~positive regulation of protein kinase cascade	7.22E-07	LEP, IL6, TNF, INS, IGF1, PRL, ADIPOQ, CTNNB1
GO:0060341~regulation of cellular localization	7.85E-07	LEP, IL6, CDKN2A, TNF, APOA1, INS, SMAD3, ADIPOQ, CPT1A
GO:0045787~positive regulation of cell cycle	8.38E-07	CCND1, TNF, CCND2, INS, ADAM17, IGF1
GO:0010552~positive regulation of specific transcription from RNA polymerase II promoter	8.38E-07	CEBPA, HES1, PPARG, TP53, SMAD3, CTNNB1
GO:0044093~positive regulation of molecular function	1.15E-06	CCND1, IL6, CDKN2A, TNF, APOA1, CCND2, INS, APOE, TP53, ADAM17, SMAD3, MYC
GO:0031401~positive regulation of protein modification process	1.54E-06	CCND1, IL6, TNF, CCND2, INS, ADAM17, IGF1, ADIPOQ
GO:0051270~regulation of cell motion	1.91E-06	IL6, CDKN1B, INS, APOE, ADAM17, SMAD3, IGF1, IGFBP3
GO:0032368~regulation of lipid transport	2.07E-06	LEP, APOA1, APOE, ITGAV, ADIPOQ
GO:0031099~regeneration	2.19E-06	CCNE1, CDKN1A, CCND1, RXRA, PPARG, IGF1
GO:0043085~positive regulation of catalytic activity	3.13E-06	CCND1, CDKN2A, TNF, APOA1, CCND2, INS, APOE, TP53, ADAM17, SMAD3, MYC
GO:0030522~intracellular receptor-mediated signaling	3.32E-06	CCNE1, AR, RXRA, ESR1, RB1, CTNNB1

pathway		
GO:0033043~regulation of organelle organization	4.15E-06	CDKN1B, TNF, INS, PRMT5, SMAD3, IGF1, MYC, CTNNB1
GO:0007584~response to nutrient	4.26E-06	CCNE1, CCND1, CDKN2B, RXRA, PPARG, ADIPOR2, ADIPOQ
GO:0045786~negative regulation of cell cycle	4.86E-06	CDKN1A, CDKN1B, CDKN2A, TP53, SMAD3, RB1
GO:0006917~induction of apoptosis	5.26E-06	CDKN1A, CDKN1B, CDKN2A, CEBPB, TNF, APOE, TP53, SMAD3, MYC
GO:0012502~induction of programmed cell death	5.38E-06	CDKN1A, CDKN1B, CDKN2A, CEBPB, TNF, APOE, TP53, SMAD3, MYC
GO:0001818~negative regulation of cytokine production	5.49E-06	TNF, APOA1, PPARG, ADAM17, ADIPOQ
GO:0006469~negative regulation of protein kinase activity	6.91E-06	CDKN1A, CDKN1B, CDKN2A, INS, APOE, RB1
GO:0010627~regulation of protein kinase cascade	1.02E-05	LEP, IL6, TNF, INS, IGF1, PRL, ADIPOQ, CTNNB1
GO:0030334~regulation of cell migration	1.26E-05	IL6, INS, APOE, ADAM17, SMAD3, IGF1, IGFBP3
GO:0042592~homeostatic process	1.26E-05	IL6, APOA1, E2F4, INS, APOE, PPARG, TP53, IGF1, RB1, MYC, ADIPOQ, CTNNB1
GO:0032868~response to insulin stimulus	1.37E-05	LEP, AR, CCND2, INS, RXRA, PPARG
GO:0043410~positive regulation of MAPKKK cascade	1.41E-05	LEP, IL6, TNF, INS, CTNNB1
GO:0032102~negative regulation of response to external stimulus	1.67E-05	LEP, INS, APOE, PPARG, ADIPOQ
GO:0043627~response to estrogen stimulus	1.73E-05	CCNE1, CCND1, CCND2, PPARG, ESR1, CTNNB1
GO:0045444~fat cell differentiation	2.10E-05	CEBPA, CCND1, CEBPB, PPARG, ADIPOQ
GO:0006919~activation of caspase activity	2.27E-05	CDKN2A, TNF, TP53, SMAD3, MYC
GO:0010564~regulation of cell cycle process	2.58E-05	CDKN2A, TNF, INS, PRMT5, IGF1, RB1
GO:0051960~regulation of nervous system development	2.59E-05	HES1, CCND2, APOE, NUMB, PPARG, TP53, IGF1
GO:0040012~regulation of locomotion	2.59E-05	IL6, INS, APOE, ADAM17, SMAD3, IGF1, IGFBP3
GO:0030518~steroid hormone receptor signaling pathway	3.02E-05	CCNE1, AR, ESR1, RB1, CTNNB1
GO:0032371~regulation of sterol transport	3.06E-05	LEP, APOA1, APOE, ADIPOQ
GO:0032374~regulation of cholesterol transport	3.06E-05	LEP, APOA1, APOE, ADIPOQ
GO:0010952~positive regulation of peptidase activity	3.23E-05	CDKN2A, TNF, TP53, SMAD3, MYC
GO:0043280~positive regulation of caspase activity	3.23E-05	CDKN2A, TNF, TP53, SMAD3, MYC
GO:0051046~regulation of secretion	3.45E-05	LEP, IL6, TNF, APOA1, INS, ADIPOQ, CPT1A
GO:0006916~anti-apoptosis	3.85E-05	CEBPB, TNF, APOE, ESR1, ADAM17, IGF1, MYC
GO:0045471~response to ethanol	4.46E-05	CCNE1, CCND1, CCND2, APOE, ADIPOQ
GO:0042493~response to drug	5.02E-05	CCNE1, CDKN1A, CCND1, PPARG, TP53, ADAM17, ADIPOQ

GO:0002521~leukocyte differentiation	5.03E-05	CEBPA, TNF, PPARG, TP53, ADAM17, CTNNB1
GO:0032870~cellular response to hormone stimulus	5.41E-05	CCND2, INS, RXRA, PPARG, ADIPOR2, ADIPOR1
GO:0019221~cytokine-mediated signaling pathway	6.34E-05	LEP, CEBPA, IL6, TNF, ADIPOQ
GO:0045637~regulation of myeloid cell differentiation	6.34E-05	LEP, TNF, RB1, ADIPOQ, CTNNB1
GO:0031100~organ regeneration	6.88E-05	CCNE1, CDKN1A, CCND1, PPARG
GO:0030097~hemopoiesis	8.20E-05	CEBPA, TNF, PPARG, TP53, ADAM17, RB1, CTNNB1
GO:0048469~cell maturation	8.31E-05	CDKN1C, CEBPA, HES1, PPARG, CTNNB1
GO:0050727~regulation of inflammatory response	8.76E-05	IL6, INS, APOE, PPARG, ADIPOQ
GO:0010871~negative regulation of receptor biosynthetic process	9.35E-05	ITGAV, PPARG, ADIPOQ
GO:0043281~regulation of caspase activity	1.02E-04	CDKN2A, TNF, TP53, SMAD3, MYC
GO:0006006~glucose metabolic process	1.05E-04	LEP, TNF, INS, MYC, ADIPOQ, CPT1A
GO:0043434~response to peptide hormone stimulus	1.08E-04	LEP, AR, CCND2, INS, RXRA, PPARG
GO:0008637~apoptotic mitochondrial changes	1.18E-04	CDKN2A, TP53, SMAD3, MYC
GO:0050728~negative regulation of inflammatory response	1.18E-04	INS, APOE, PPARG, ADIPOQ
GO:0052548~regulation of endopeptidase activity	1.18E-04	CDKN2A, TNF, TP53, SMAD3, MYC
GO:0010638~positive regulation of organelle organization	1.23E-04	CDKN1B, TNF, INS, SMAD3, IGF1
GO:0048534~hemopoietic or lymphoid organ development	1.40E-04	CEBPA, TNF, PPARG, TP53, ADAM17, RB1, CTNNB1
GO:0052547~regulation of peptidase activity	1.42E-04	CDKN2A, TNF, TP53, SMAD3, MYC
GO:0050767~regulation of neurogenesis	1.54E-04	HES1, CCND2, APOE, NUMB, PPARG, TP53
GO:0030335~positive regulation of cell migration	1.62E-04	IL6, INS, ADAM17, SMAD3, IGF1
GO:0010906~regulation of glucose metabolic process	1.70E-04	LEP, INS, IGF1, ADIPOQ
GO:0000075~cell cycle checkpoint	1.76E-04	CCND1, CDKN2A, CDKN2B, TP53, RB1
GO:0030521~androgen receptor signaling pathway	1.85E-04	CCNE1, AR, RB1, CTNNB1
GO:0019218~regulation of steroid metabolic process	1.85E-04	LEP, TNF, APOA1, APOE
GO:0031331~positive regulation of cellular catabolic process	1.85E-04	TNF, INS, APOE, IGF1
GO:0031348~negative regulation of defense response	1.85E-04	INS, APOE, PPARG, ADIPOQ
GO:0002520~immune system development	1.93E-04	CEBPA, TNF, PPARG, TP53, ADAM17, RB1, CTNNB1
GO:0032722~positive regulation of chemokine production	1.96E-04	IL6, TNF, ADAM17
GO:0010675~regulation of cellular carbohydrate metabolic	2.18E-04	LEP, INS, IGF1, ADIPOQ

process		
GO:0051345~positive regulation of hydrolase activity	2.19E-04	CDKN2A, TNF, APOA1, TP53, SMAD3, MYC
GO:0051130~positive regulation of cellular component organization	2.31E-04	CDKN1B, TNF, INS, NUMB, SMAD3, IGF1
GO:0051272~positive regulation of cell motion	2.35E-04	IL6, INS, ADAM17, SMAD3, IGF1
GO:0040017~positive regulation of locomotion	2.35E-04	IL6, INS, ADAM17, SMAD3, IGF1
GO:0034440~lipid oxidation	2.35E-04	ADIPOR2, ADIPOR1, ADIPOQ, CPT1A
GO:0019395~fatty acid oxidation	2.35E-04	ADIPOR2, ADIPOR1, ADIPOQ, CPT1A
GO:0017015~regulation of transforming growth factor beta receptor signaling pathway	2.35E-04	CDKN1C, CDKN2B, ADAM17, SMAD3
GO:0006109~regulation of carbohydrate metabolic process	2.35E-04	LEP, INS, IGF1, ADIPOQ
GO:0001775~cell activation	2.39E-04	IL6, TNF, INS, TP53, ADAM17, SMAD3, CTNNB1
GO:0048585~negative regulation of response to stimulus	2.54E-04	LEP, INS, APOE, PPARG, ADIPOQ
GO:0045737~positive regulation of cyclin-dependent protein kinase activity	2.60E-04	CCND1, CCND2, ADAM17
GO:0006111~regulation of gluconeogenesis	2.60E-04	LEP, INS, ADIPOQ
GO:0006351~transcription, DNA-dependent	2.62E-04	CEBPA, AR, CDKN2A, CEBPB, ESR1, TP53, MYC
GO:0021700~developmental maturation	2.63E-04	CDKN1C, CEBPA, HES1, PPARG, CTNNB1
GO:0010553~negative regulation of specific transcription from RNA polymerase II promoter	2.73E-04	HES1, RXRA, PPARG, SMAD3
GO:0002761~regulation of myeloid leukocyte differentiation	2.73E-04	TNF, RB1, ADIPOQ, CTNNB1
GO:0032774~RNA biosynthetic process	2.82E-04	CEBPA, AR, CDKN2A, CEBPB, ESR1, TP53, MYC
GO:0001649~osteoblast differentiation	2.94E-04	INS, SMAD3, IGF1, IGFBP3
GO:0008219~cell death	2.95E-04	AR, IL6, CDKN1B, CDKN2A, TNF, INS, TP53, SMAD3, MYC, CTNNB1
GO:0019318~hexose metabolic process	3.04E-04	LEP, TNF, INS, MYC, ADIPOQ, CPT1A
GO:0016265~death	3.10E-04	AR, IL6, CDKN1B, CDKN2A, TNF, INS, TP53, SMAD3, MYC, CTNNB1
GO:0006350~transcription	3.18E-04	CEBPA, AR, CEBPB, E2F4, RBL2, RXRA, RBL1, PPARG, TP53, ESR1, SMAD3, RB1, CTNNB1, HES1, CDKN2A, PRMT5, MYC
GO:0010869~regulation of receptor biosynthetic process	3.34E-04	ITGAV, PPARG, ADIPOQ
GO:0043408~regulation of MAPKKK cascade	3.53E-04	LEP, IL6, TNF, INS, CTNNB1
GO:0046890~regulation of lipid biosynthetic process	3.61E-04	LEP, TNF, INS, APOE

GO:0048660~regulation of smooth muscle cell proliferation	3.85E-04	IL6, TNF, IGF1, IGFBP3
GO:0008406~gonad development	3.91E-04	LEP, CCNE1, CCND1, AR, CCND2
GO:0050731~positive regulation of peptidyl-tyrosine phosphorylation	4.10E-04	IL6, INS, IGF1, ADIPOQ
GO:0043550~regulation of lipid kinase activity	4.17E-04	RBL2, RBL1, RB1
GO:0051223~regulation of protein transport	4.18E-04	IL6, TNF, APOA1, INS, SMAD3
GO:0051052~regulation of DNA metabolic process	4.18E-04	INS, TP53, PCNA, IGF1, MYC
GO:0009896~positive regulation of catabolic process	4.64E-04	TNF, INS, APOE, IGF1
GO:0045736~negative regulation of cyclin-dependent protein kinase activity	5.08E-04	CDKN1A, CDKN1B, CDKN2A
GO:0055088~lipid homeostasis	5.22E-04	APOA1, INS, APOE, PPARG
GO:0070201~regulation of establishment of protein localization	5.24E-04	IL6, TNF, APOA1, INS, SMAD3
GO:0051336~regulation of hydrolase activity	5.62E-04	CDKN2A, TNF, APOA1, TP53, SMAD3, MYC, ANGPTL4
GO:0005996~monosaccharide metabolic process	5.90E-04	LEP, TNF, INS, MYC, ADIPOQ, CPT1A
GO:0051050~positive regulation of transport	6.02E-04	IL6, TNF, INS, APOE, SMAD3, ADIPOQ
GO:0010745~negative regulation of foam cell differentiation	6.09E-04	ITGAV, PPARG, ADIPOQ
GO:0042110~T cell activation	6.11E-04	INS, TP53, ADAM17, SMAD3, CTNNB1
GO:0048608~reproductive structure development	6.11E-04	LEP, CCNE1, CCND1, AR, CCND2
GO:0045137~development of primary sexual characteristics	6.29E-04	LEP, CCNE1, CCND1, AR, CCND2
GO:0051048~negative regulation of secretion	6.53E-04	TNF, APOA1, INS, ADIPOQ
GO:0007088~regulation of mitosis	6.88E-04	TNF, INS, PRMT5, IGF1
GO:0051783~regulation of nuclear division	6.88E-04	TNF, INS, PRMT5, IGF1
GO:0032770~positive regulation of monooxygenase activity	7.18E-04	TNF, INS, APOE
GO:0030511~positive regulation of transforming growth factor beta receptor signaling pathway	7.18E-04	CDKN1C, CDKN2B, ADAM17
GO:0010565~regulation of cellular ketone metabolic process	7.25E-04	LEP, INS, PPARG, ADIPOQ
GO:0050708~regulation of protein secretion	7.62E-04	IL6, TNF, APOA1, INS
GO:0048732~gland development	7.91E-04	HES1, AR, APOA1, IGF1, PRL
GO:0051051~negative regulation of transport	7.91E-04	TNF, APOA1, INS, ITGAV, ADIPOQ
GO:0045940~positive regulation of steroid metabolic process	8.36E-04	TNF, APOA1, APOE

GO:0051347~positive regulation of transferase activity	8.39E-04	CCND1, TNF, APOA1, CCND2, INS, ADAM17
GO:0001890~placenta development	8.42E-04	CEBPA, CEBPB, RXRA, PPARG
GO:0007005~mitochondrion organization	8.59E-04	CEBPA, CDKN2A, TP53, SMAD3, MYC
GO:0032880~regulation of protein localization	8.59E-04	IL6, TNF, APOA1, INS, SMAD3
GO:0065003~macromolecular complex assembly	8.73E-04	APOA1, APOE, PRMT5, TP53, SMAD3, MYC, ADIPOQ, CTNNB1, ANGPTL4
GO:0031329~regulation of cellular catabolic process	8.84E-04	TNF, INS, APOE, IGF1
GO:0040014~regulation of multicellular organism growth	8.84E-04	LEP, APOE, IGF1, PRL
GO:0048878~chemical homeostasis	9.11E-04	APOA1, INS, APOE, PPARG, TP53, IGF1, MYC, ADIPOQ
GO:0006275~regulation of DNA replication	9.27E-04	INS, TP53, PCNA, IGF1
GO:0070482~response to oxygen levels	9.31E-04	CDKN1A, ADAM17, SMAD3, ADIPOQ, ANGPTL4
GO:0032642~regulation of chemokine production	9.63E-04	IL6, TNF, ADAM17
GO:0031668~cellular response to extracellular stimulus	0.001016354	CCNE1, CDKN1A, CDKN2B, TP53
GO:0034381~lipoprotein particle clearance	0.001098488	APOA1, APOE, ADIPOQ
GO:0050709~negative regulation of protein secretion	0.001098488	TNF, APOA1, INS
GO:0051412~response to corticosterone stimulus	0.001098488	CCNE1, CDKN1A, CCND1
GO:0033273~response to vitamin	0.001111475	CCNE1, CCND1, RXRA, PPARG
GO:0046883~regulation of hormone secretion	0.001111475	LEP, TNF, ADIPOQ, CPT1A
GO:0009611~response to wounding	0.001114668	IL6, CEBPB, TNF, INS, RXRA, ADAM17, SMAD3, IGF1
GO:0051222~positive regulation of protein transport	0.001161074	IL6, TNF, INS, SMAD3
GO:0007548~sex differentiation	0.00120068	LEP, CCNE1, CCND1, AR, CCND2
GO:0032869~cellular response to insulin stimulus	0.00121205	CCND2, INS, RXRA, PPARG
GO:0050730~regulation of peptidyl-tyrosine phosphorylation	0.00121205	IL6, INS, IGF1, ADIPOQ
GO:0031575~G1/S transition checkpoint	0.001242506	CCND1, CDKN2B, TP53
GO:0030258~lipid modification	0.001264418	ADIPOR2, ADIPOR1, ADIPOQ, CPT1A
GO:0043933~macromolecular complex subunit organization	0.001334028	APOA1, APOE, PRMT5, TP53, SMAD3, MYC, ADIPOQ, CTNNB1, ANGPTL4
GO:0045445~myoblast differentiation	0.001556132	IGF1, RB1, CTNNB1
GO:0051385~response to mineralocorticoid stimulus	0.001725638	CCNE1, CDKN1A, CCND1
GO:0034097~response to cytokine stimulus	0.001867785	CCNE1, CDKN2B, ADIPOQ, CTNNB1
GO:0002673~regulation of acute inflammatory response	0.001903537	IL6, INS, PPARG
GO:0043255~regulation of carbohydrate biosynthetic process	0.001903537	LEP, INS, ADIPOQ

GO:0001836~release of cytochrome c from mitochondria	0.001903537	TP53, SMAD3, MYC
GO:0001701~in utero embryonic development	0.002107516	CEBPA, HES1, AR, CEBPB, TP53
GO:0030182~neuron differentiation	0.00217274	LEP, CDKN1C, HES1, IL6, CEBPB, RXRA, NUMB
GO:0050873~brown fat cell differentiation	0.002487081	CEBPA, PPARG, ADIPOQ
GO:0012501~programmed cell death	0.002517525	IL6, CDKN1B, CDKN2A, TNF, TP53, SMAD3, MYC, CTNNB1
GO:0010743~regulation of foam cell differentiation	0.002698042	ITGAV, PPARG, ADIPOQ
GO:0045840~positive regulation of mitosis	0.002698042	TNF, INS, IGF1
GO:0032768~regulation of monooxygenase activity	0.002698042	TNF, INS, APOE
GO:0050810~regulation of steroid biosynthetic process	0.002698042	LEP, TNF, APOE
GO:0050680~negative regulation of epithelial cell proliferation	0.002698042	CDKN1C, CDKN1B, CDKN2B
GO:0051785~positive regulation of nuclear division	0.002698042	TNF, INS, IGF1
GO:0001819~positive regulation of cytokine production	0.002709955	IL6, TNF, ADAM17, SMAD3
GO:0042060~wound healing	0.002834476	IL6, INS, ADAM17, SMAD3, IGF1
GO:0008203~cholesterol metabolic process	0.002884384	LEP, APOA1, APOE, RXRA
GO:0046427~positive regulation of JAK-STAT cascade	0.002917142	IL6, IGF1, PRL
GO:0051353~positive regulation of oxidoreductase activity	0.002917142	TNF, INS, APOE
GO:0001501~skeletal system development	0.00293922	INS, SMAD3, IGF1, IGFBP3, MYC, CTNNB1
GO:0030099~myeloid cell differentiation	0.002974135	CEBPA, TNF, PPARG, RB1
GO:0033077~T cell differentiation in the thymus	0.003144331	TP53, ADAM17, CTNNB1
GO:0006631~fatty acid metabolic process	0.003226247	LEP, ADIPOR2, ADIPOR1, ADIPOQ, CPT1A
GO:0009894~regulation of catabolic process	0.003253671	TNF, INS, APOE, IGF1
GO:0046649~lymphocyte activation	0.003285081	INS, TP53, ADAM17, SMAD3, CTNNB1
GO:0045833~negative regulation of lipid metabolic process	0.003379559	TNF, INS, APOE
GO:0008202~steroid metabolic process	0.003465984	LEP, APOA1, APOE, RXRA, CYP19A1
GO:0030856~regulation of epithelial cell differentiation	0.003622776	CCND1, CDKN2B, CTNNB1
GO:0050921~positive regulation of chemotaxis	0.003622776	IL6, ADAM17, SMAD3
GO:0010035~response to inorganic substance	0.003653578	CDKN1A, CCND1, APOE, RXRA, CTNNB1
GO:0016125~sterol metabolic process	0.003754454	LEP, APOA1, APOE, RXRA
GO:0002697~regulation of immune effector process	0.003754454	IL6, TNF, APOA1, INS

GO:0048666~neuron development	0.003813486	LEP, CDKN1C, HES1, IL6, RXRA, NUMB
GO:0050707~regulation of cytokine secretion	0.003873933	TNF, APOA1, INS
GO:0010608~posttranscriptional regulation of gene expression	0.004049314	IL6, CDKN2A, TNF, APOA1, SMAD3
GO:0048661~positive regulation of smooth muscle cell proliferation	0.004132981	IL6, TNF, IGF1
GO:0050920~regulation of chemotaxis	0.004132981	IL6, ADAM17, SMAD3
GO:0046324~regulation of glucose import	0.004674553	TNF, INS, ADIPOQ
GO:0045860~positive regulation of protein kinase activity	0.004926223	CCND1, TNF, CCND2, INS, ADAM17
GO:0002573~myeloid leukocyte differentiation	0.004956979	CEBPA, TNF, PPARG
GO:0048520~positive regulation of behavior	0.004956979	IL6, ADAM17, SMAD3
GO:0000060~protein import into nucleus, translocation	0.004956979	TNF, TP53, PRL
GO:0010827~regulation of glucose transport	0.004956979	TNF, INS, ADIPOQ
GO:0048145~regulation of fibroblast proliferation	0.005247102	CDKN1A, TP53, IGF1
GO:0009628~response to abiotic stimulus	0.005394284	CDKN1A, CCND1, TNF, PPARG, TP53, MYC
GO:0001503~ossification	0.005398001	INS, SMAD3, IGF1, IGFBP3
GO:0009062~fatty acid catabolic process	0.005544872	LEP, ADIPOQ, CPT1A
GO:0032675~regulation of interleukin-6 production	0.005544872	IL6, CEBPB, TNF
GO:0046425~regulation of JAK-STAT cascade	0.005544872	IL6, IGF1, PRL
GO:0033674~positive regulation of kinase activity	0.005577025	CCND1, TNF, CCND2, INS, ADAM17
GO:0048584~positive regulation of response to stimulus	0.006011774	IL6, TNF, INS, ADAM17, SMAD3
GO:0033197~response to vitamin E	0.006199933	CCNE1, CCND1
GO:0042692~muscle cell differentiation	0.0062156	RXRA, IGF1, RB1, CTNNB1
GO:0060348~bone development	0.006503689	INS, SMAD3, IGF1, IGFBP3
GO:0045321~leukocyte activation	0.006562728	INS, TP53, ADAM17, SMAD3, CTNNB1
GO:0051240~positive regulation of multicellular organismal process	0.006753606	IL6, TNF, INS, ADAM17, SMAD3
GO:0051341~regulation of oxidoreductase activity	0.006811472	TNF, INS, APOE
GO:0006953~acute-phase response	0.006811472	IL6, CEBPB, INS
GO:0050796~regulation of insulin secretion	0.006811472	LEP, TNF, CPT1A
GO:0051224~negative regulation of protein transport	0.006811472	TNF, APOA1, INS

GO:0002700~regulation of production of molecular mediator of immune response	0.007146765	IL6, TNF, APOA1
GO:0050679~positive regulation of epithelial cell proliferation	0.007146765	CCND2, IGF1, PRL
GO:0050714~positive regulation of protein secretion	0.007146765	IL6, TNF, INS
GO:0045667~regulation of osteoblast differentiation	0.007839393	IL6, SMAD3, CTNNB1
GO:0007093~mitotic cell cycle checkpoint	0.007839393	CCND1, CDKN2B, TP53
GO:0045664~regulation of neuron differentiation	0.008063277	HES1, CCND2, APOE, NUMB
GO:0031669~cellular response to nutrient levels	0.008196635	CCNE1, CDKN2B, TP53
GO:0001666~response to hypoxia	0.008230309	ADAM17, SMAD3, ADIPOQ, ANGPTL4
GO:0003006~reproductive developmental process	0.008639925	LEP, CCNE1, CCND1, AR, CCND2
GO:0030155~regulation of cell adhesion	0.008743649	CDKN2A, TNF, SMAD3, ADIPOQ
GO:0002791~regulation of peptide secretion	0.008932744	LEP, TNF, CPT1A
GO:0050795~regulation of behavior	0.008932744	IL6, ADAM17, SMAD3
GO:0045165~cell fate commitment	0.009096134	HES1, PPARG, TP53, CTNNB1
GO:0070587~regulation of cell-cell adhesion involved in gastrulation	0.009285813	TNF, ADIPOQ
GO:0045721~negative regulation of gluconeogenesis	0.009285813	INS, ADIPOQ
GO:0034114~regulation of heterotypic cell-cell adhesion	0.009285813	TNF, ADIPOQ
GO:0045715~negative regulation of low-density lipoprotein receptor biosynthetic process	0.009285813	ITGAV, ADIPOQ
GO:0010470~regulation of gastrulation	0.009285813	TNF, ADIPOQ
GO:0051346~negative regulation of hydrolase activity	0.00969738	APOA1, TP53, ANGPTL4
GO:0033500~carbohydrate homeostasis	0.010090281	INS, PPARG, ADIPOQ
GO:0042593~glucose homeostasis	0.010090281	INS, PPARG, ADIPOQ
GO:0019217~regulation of fatty acid metabolic process	0.010090281	INS, PPARG, ADIPOQ
GO:0006915~apoptosis	0.010148168	IL6, CDKN2A, TNF, TP53, SMAD3, MYC, CTNNB1
GO:0050864~regulation of B cell activation	0.010897024	CDKN1A, IL6, CDKN2A
GO:0030162~regulation of proteolysis	0.011731389	TNF, INS, APOE
GO:0051098~regulation of binding	0.011796833	IL6, CDKN2A, TNF, SMAD3
GO:0008629~induction of apoptosis by intracellular signals	0.012158817	CDKN1A, TP53, MYC
GO:0002740~negative regulation of cytokine secretion during	0.012362337	TNF, APOA1

immune response		
GO:0055094~response to lipoprotein stimulus	0.012362337	PPARG, ADAM17
GO:0002739~regulation of cytokine secretion during immune response	0.012362337	TNF, APOA1
GO:0032800~receptor biosynthetic process	0.012362337	TNF, PPARG
GO:0032488~Cdc42 protein signal transduction	0.012362337	APOA1, APOE
GO:0002674~negative regulation of acute inflammatory response	0.012362337	INS, PPARG
GO:0045714~regulation of low-density lipoprotein receptor biosynthetic process	0.012362337	ITGAV, ADIPOQ
GO:0010876~lipid localization	0.012644502	TNF, APOA1, APOE, PPARG
GO:0051301~cell division	0.01293704	CCNE1, CCND1, CDKN2A, CCND2, RB1
GO:0046822~regulation of nucleocytoplasmic transport	0.01393581	CDKN2A, TNF, SMAD3
GO:0009411~response to UV	0.014396659	CDKN1A, CCND1, TP53
GO:0048598~embryonic morphogenesis	0.014786646	HES1, TP53, SMAD3, MYC, CTNNB1
GO:0044057~regulation of system process	0.015110415	LEP, TNF, APOA1, INS, APOE
GO:0007494~midgut development	0.015429535	RXRA, CTNNB1
GO:0034380~high-density lipoprotein particle assembly	0.015429535	APOA1, APOE
GO:0022602~ovulation cycle process	0.015818354	LEP, CCNE1, CCND2
GO:0010243~response to organic nitrogen	0.016305159	CCNE1, CDKN1A, CCND1
GO:0051271~negative regulation of cell motion	0.016305159	CDKN1B, APOE, IGFBP3
GO:0016042~lipid catabolic process	0.016379517	LEP, APOE, ADIPOQ, CPT1A
GO:0051259~protein oligomerization	0.016631411	TP53, ADIPOQ, CTNNB1, ANGPTL4
GO:0032103~positive regulation of response to external stimulus	0.016798347	IL6, ADAM17, SMAD3
GO:0008585~female gonad development	0.016798347	LEP, CCNE1, CCND2
GO:0050865~regulation of cell activation	0.01688549	CDKN1A, IL6, CDKN2A, APOE
GO:0030217~T cell differentiation	0.017297874	TP53, ADAM17, CTNNB1
GO:0006954~inflammatory response	0.017863629	IL6, CEBPB, TNF, INS, RXRA
GO:0032269~negative regulation of cellular protein metabolic process	0.018188718	CDKN2A, INS, ITGAV, IGFBP3

GO:0042698~ovulation cycle	0.01831578	LEP, CCNE1, CCND2
GO:0000083~regulation of transcription of G1/S-phase of mitotic cell cycle	0.018487433	E2F4, RB1
GO:0030300~regulation of intestinal cholesterol absorption	0.018487433	LEP, APOA1
GO:0010875~positive regulation of cholesterol efflux	0.018487433	APOE, ADIPOQ
GO:0034384~high-density lipoprotein particle clearance	0.018487433	APOA1, APOE
GO:0006955~immune response	0.018863965	IL6, CEBPB, TNF, PPARG, TP53, ADAM17, SMAD3
GO:0043009~chordate embryonic development	0.01897204	CEBPA, HES1, AR, CEBPB, TP53
GO:0040007~growth	0.01899698	CCNE1, AR, RXRA, IGF1
GO:0046660~female sex differentiation	0.019358542	LEP, CCNE1, CCND2
GO:0046545~development of primary female sexual characteristics	0.019358542	LEP, CCNE1, CCND2
GO:0035270~endocrine system development	0.019358542	HES1, IL6, APOA1
GO:0010720~positive regulation of cell development	0.019358542	NUMB, PPARG, SMAD3
GO:0006461~protein complex assembly	0.019377255	TP53, SMAD3, MYC, ADIPOQ, CTNNB1, ANGPTL4
GO:0070271~protein complex biogenesis	0.019377255	TP53, SMAD3, MYC, ADIPOQ, CTNNB1, ANGPTL4
GO:0009792~embryonic development ending in birth or egg hatching	0.019542026	CEBPA, HES1, AR, CEBPB, TP53
GO:0003013~circulatory system process	0.01982503	E2F4, APOE, PPARG, ADIPOQ
GO:0008015~blood circulation	0.01982503	E2F4, APOE, PPARG, ADIPOQ
GO:0007369~gastrulation	0.01988914	TP53, SMAD3, CTNNB1
GO:0051248~negative regulation of protein metabolic process	0.020105449	CDKN2A, INS, ITGAV, IGFBP3
GO:0032386~regulation of intracellular transport	0.020425829	CDKN2A, TNF, SMAD3
GO:0032376~positive regulation of cholesterol transport	0.02153606	APOE, ADIPOQ
GO:0032373~positive regulation of sterol transport	0.02153606	APOE, ADIPOQ
GO:0045821~positive regulation of glycolysis	0.02153606	INS, IGF1
GO:0010873~positive regulation of cholesterol esterification	0.02153606	APOA1, APOE
GO:0010874~regulation of cholesterol efflux	0.02153606	APOE, ADIPOQ
GO:0042035~regulation of cytokine biosynthetic process	0.022072024	IL6, CEBPB, TNF
GO:0044242~cellular lipid catabolic process	0.023199191	LEP, ADIPOQ, CPT1A

GO:0010769~regulation of cell morphogenesis involved in differentiation	0.023771564	APOE, NUMB, SMAD3
GO:0009314~response to radiation	0.02395161	CDKN1A, CCND1, TP53, MYC
GO:0000902~cell morphogenesis	0.024048867	HES1, E2F4, RXRA, NUMB, CTNNB1
GO:0030278~regulation of ossification	0.024349744	IL6, SMAD3, CTNNB1
GO:0010872~regulation of cholesterol esterification	0.024575442	APOA1, APOE
GO:0033032~regulation of myeloid cell apoptosis	0.024575442	ADAM17, ADIPOQ
GO:0031670~cellular response to nutrient	0.024575442	CCNE1, CDKN2B
GO:0050670~regulation of lymphocyte proliferation	0.027326361	CDKN1A, IL6, CDKN2A
GO:0002446~neutrophil mediated immunity	0.027605607	IL6, ADAM17
GO:0051023~regulation of immunoglobulin secretion	0.027605607	IL6, TNF
GO:0045912~negative regulation of carbohydrate metabolic process	0.027605607	INS, ADIPOQ
GO:0051000~positive regulation of nitric-oxide synthase activity	0.027605607	INS, APOE
GO:0010677~negative regulation of cellular carbohydrate metabolic process	0.027605607	INS, ADIPOQ
GO:0051044~positive regulation of membrane protein ectodomain proteolysis	0.027605607	TNF, APOE
GO:0032944~regulation of mononuclear cell proliferation	0.027938553	CDKN1A, IL6, CDKN2A
GO:0070663~regulation of leukocyte proliferation	0.027938553	CDKN1A, IL6, CDKN2A
GO:0006606~protein import into nucleus	0.029179497	TNF, TP53, PRL
GO:0033554~cellular response to stress	0.029913171	CDKN1A, CCND1, TNF, RXRA, TP53, PCNA
GO:0051170~nuclear import	0.030442263	TNF, TP53, PRL
GO:0051146~striated muscle cell differentiation	0.030442263	RXRA, IGF1, RB1
GO:0035295~tube development	0.03059707	CEBPA, HES1, IGF1, CTNNB1
GO:0045073~regulation of chemokine biosynthetic process	0.030626582	IL6, TNF
GO:0030858~positive regulation of epithelial cell differentiation	0.030626582	CDKN2B, CTNNB1
GO:0050710~negative regulation of cytokine secretion	0.030626582	TNF, APOA1
GO:0033700~phospholipid efflux	0.030626582	APOA1, APOE
GO:0002690~positive regulation of leukocyte chemotaxis	0.030626582	IL6, ADAM17

GO:0014074~response to purine	0.030626582	CCNE1, PPARG
GO:0043523~regulation of neuron apoptosis	0.031726547	TNF, ESR1, TP53
GO:0032370~positive regulation of lipid transport	0.033638395	APOE, ADIPOQ
GO:0002688~regulation of leukocyte chemotaxis	0.033638395	IL6, ADAM17
GO:0045649~regulation of macrophage differentiation	0.033638395	RB1, ADIPOQ
GO:0031571~G1 DNA damage checkpoint	0.033638395	CCND1, TP53
GO:0044058~regulation of digestive system process	0.033638395	LEP, APOA1
GO:0007423~sensory organ development	0.033874479	HES1, CDKN1B, MYC, CTNNB1
GO:0032989~cellular component morphogenesis	0.034027538	HES1, E2F4, RXRA, NUMB, CTNNB1
GO:0034504~protein localization in nucleus	0.034358468	TNF, TP53, PRL
GO:0043583~ear development	0.035029429	HES1, CDKN1B, MYC
GO:0019932~second-messenger-mediated signaling	0.036157689	TNF, APOE, PCNA, IGF1
GO:0014068~positive regulation of phosphoinositide 3-kinase cascade	0.036641072	INS, IGF1
GO:0065005~protein-lipid complex assembly	0.036641072	APOA1, APOE
GO:0010888~negative regulation of lipid storage	0.036641072	ITGAV, PPARG
GO:0034377~plasma lipoprotein particle assembly	0.036641072	APOA1, APOE
GO:0006110~regulation of glycolysis	0.036641072	INS, IGF1
GO:0014910~regulation of smooth muscle cell migration	0.036641072	IGF1, IGFBP3
GO:0002526~acute inflammatory response	0.037072879	IL6, CEBPB, INS
GO:0007267~cell-cell signaling	0.037118341	LEP, AR, IL6, INS, APOE, CTNNB1
GO:0030324~lung development	0.037764096	CEBPA, HES1, CTNNB1
GO:0034613~cellular protein localization	0.037917059	TNF, TP53, PCNA, PRL, CTNNB1
GO:0070727~cellular macromolecule localization	0.03878283	TNF, TP53, PCNA, PRL, CTNNB1
GO:0042993~positive regulation of transcription factor import into nucleus	0.039634641	TNF, SMAD3
GO:0014066~regulation of phosphoinositide 3-kinase cascade	0.039634641	INS, IGF1
GO:0002021~response to dietary excess	0.039634641	LEP, APOE
GO:0051043~regulation of membrane protein ectodomain proteolysis	0.039634641	TNF, APOE

GO:0045995~regulation of embryonic development	0.039634641	TNF, ADIPOQ
GO:0050995~negative regulation of lipid catabolic process	0.039634641	TNF, INS
GO:0000904~cell morphogenesis involved in differentiation	0.039728888	HES1, RXRA, NUMB, CTNNB1
GO:0030323~respiratory tube development	0.039867444	CEBPA, HES1, CTNNB1
GO:0001568~blood vessel development	0.040136472	APOE, ITGAV, CTNNB1, ANGPTL4
GO:0030098~lymphocyte differentiation	0.04057834	TP53, ADAM17, CTNNB1
GO:0051090~regulation of transcription factor activity	0.04057834	IL6, CDKN2A, TNF
GO:0006952~defense response	0.040613362	IL6, CEBPB, TNF, INS, RXRA, PPARG
GO:0007265~Ras protein signal transduction	0.042014588	APOA1, APOE, IGF1
GO:0009651~response to salt stress	0.042619128	TNF, TP53
GO:0001944~vasculature development	0.042626997	APOE, ITGAV, CTNNB1, ANGPTL4
GO:0060541~respiratory system development	0.044204576	CEBPA, HES1, CTNNB1
GO:0051047~positive regulation of secretion	0.04494393	IL6, TNF, INS
GO:0002444~myeloid leukocyte mediated immunity	0.045594561	IL6, ADAM17
GO:0002687~positive regulation of leukocyte migration	0.045594561	IL6, ADAM17
GO:0010165~response to X-ray	0.045594561	CCND1, TP53
GO:0007568~aging	0.045687906	CDKN2A, RXRA, TP53
GO:0007565~female pregnancy	0.045687906	RXRA, ADIPOR2, PRL
GO:0046395~carboxylic acid catabolic process	0.046436469	LEP, ADIPOQ, CPT1A
GO:0016054~organic acid catabolic process	0.046436469	LEP, ADIPOQ, CPT1A
GO:0043470~regulation of carbohydrate catabolic process	0.048560966	INS, IGF1
GO:0043691~reverse cholesterol transport	0.048560966	APOA1, APOE
GO:0043471~regulation of cellular carbohydrate catabolic process	0.048560966	INS, IGF1
GO:0045598~regulation of fat cell differentiation	0.048560966	PPARG, ADIPOQ
GO:0050999~regulation of nitric-oxide synthase activity	0.048560966	INS, APOE
**Term**	**PValue**	**Genes**
**Cellular component**		
GO:0005654~nucleoplasm	1.11E-06	CEBPA, E2F4, RBL2, RBL1, TP53, SMAD3, RB1, CTNNB1, CCNE1, CCND1, CDKN1A, CDKN2A, PCNA, MYC

GO:0043233~organelle lumen	1.67E-06	CEBPA, CEBPB, E2F4, RBL2, RBL1, TP53, SMAD3, IGF1, RB1, CTNNB1, CDKN1C, CCNE1, CCND1, CDKN1A, CDKN2A, APOA1, INS, PCNA, MYC
GO:0031974~membrane-enclosed lumen	2.23E-06	CEBPA, CEBPB, E2F4, RBL2, RBL1, TP53, SMAD3, IGF1, RB1, CTNNB1, CDKN1C, CCNE1, CCND1, CDKN1A, CDKN2A, APOA1, INS, PCNA, MYC
GO:0005667~transcription factor complex	2.88E-06	CEBPA, E2F4, RBL2, RBL1, TP53, SMAD3, RB1, CTNNB1
GO:0000307~cyclin-dependent protein kinase holoenzyme complex	4.26E-06	CDKN1A, CCND1, CCND2, PCNA
GO:0070013~intracellular organelle lumen	6.19E-06	CEBPA, CEBPB, E2F4, RBL2, RBL1, TP53, SMAD3, RB1, CTNNB1, CDKN1C, CCNE1, CCND1, CDKN1A, CDKN2A, APOA1, INS, PCNA, MYC
GO:0031981~nuclear lumen	1.10E-05	CEBPA, CEBPB, E2F4, RBL2, RBL1, TP53, SMAD3, RB1, CTNNB1, CDKN1C, CCNE1, CCND1, CDKN1A, CDKN2A, PCNA, MYC
GO:0005615~extracellular space	1.72E-04	LEP, IL6, TNF, APOA1, INS, APOE, IGF1, IGFBP3, ADIPOQ, ANGPTL4
GO:0044451~nucleoplasm part	2.24E-04	CEBPA, E2F4, RBL2, RBL1, TP53, SMAD3, RB1, MYC, CTNNB1
GO:0044421~extracellular region part	0.001987952	LEP, IL6, TNF, APOA1, INS, APOE, IGF1, IGFBP3, ADIPOQ, ANGPTL4
GO:0019897~extrinsic to plasma membrane	0.010132634	APOE, NUMB, CTNNB1
GO:0016942~insulin-like growth factor binding protein complex	0.012150338	IGF1, IGFBP3
GO:0034399~nuclear periphery	0.014821751	CEBPB, TP53, PCNA
GO:0043005~neuron projection	0.019826451	AR, APOE, RXRA, ESR1, CTNNB1
GO:0043235~receptor complex	0.048661009	IL6, ITGAV, SMAD3
**Term**	**PValue**	**Genes**
**Molecular function**		
GO:0016538~cyclin-dependent protein kinase regulator activity	1.67E-09	CDKN1C, CDKN1A, CCND1, CDKN1B, CDKN2A, CDKN2B
GO:0004861~cyclin-dependent protein kinase inhibitor activity	1.96E-08	CDKN1C, CDKN1A, CDKN1B, CDKN2A, CDKN2B
GO:0030291~protein serine/threonine kinase inhibitor activity	2.18E-07	CDKN1C, CDKN1A, CDKN1B, CDKN2A, CDKN2B
GO:0010843~promoter binding	1.02E-06	CEBPA, RXRA, ESR1, TP53, SMAD3, CTNNB1
GO:0008134~transcription factor binding	4.00E-06	CEBPA, CCNE1, CDKN2A, E2F4, RXRA, PPARG, RBL1, TP53, SMAD3, RB1, CTNNB1
GO:0004860~protein kinase inhibitor activity	5.17E-06	CDKN1C, CDKN1A, CDKN1B, CDKN2A, CDKN2B
GO:0019210~kinase inhibitor activity	5.78E-06	CDKN1C, CDKN1A, CDKN1B, CDKN2A, CDKN2B
GO:0019887~protein kinase regulator activity	5.92E-06	CDKN1C, CDKN1A, CCND1, CDKN1B, CDKN2A, CDKN2B

GO:0051427~hormone receptor binding	8.91E-06	LEP, CCNE1, RXRA, PPARG, RB1, CTNNB1
GO:0019207~kinase regulator activity	1.17E-05	CDKN1C, CDKN1A, CCND1, CDKN1B, CDKN2A, CDKN2B
GO:0003690~double-stranded DNA binding	1.43E-05	RXRA, TP53, PCNA, SMAD3, MYC, CTNNB1
GO:0019900~kinase binding	2.19E-05	CCND1, CDKN2A, CDKN2B, CCND2, SMAD3, RB1, CTNNB1
GO:0032403~protein complex binding	3.66E-05	CCNE1, CDKN1A, CCND1, INS, PCNA, ADAM17, IGF1
GO:0003700~transcription factor activity	4.02E-05	CEBPA, AR, CDKN2A, E2F4, CEBPB, RXRA, PPARG, ESR1, TP53, SMAD3, RB1, MYC, CTNNB1
GO:0030528~transcription regulator activity	4.22E-05	CEBPA, AR, CEBPB, E2F4, RXRA, PPARG, TP53, ESR1, SMAD3, RB1, CTNNB1, HES1, CDKN1C, CCNE1, CDKN2A, MYC
GO:0016563~transcription activator activity	4.25E-05	CDKN1C, CCNE1, AR, CEBPB, RXRA, PPARG, SMAD3, RB1, CTNNB1
GO:0046983~protein dimerization activity	4.68E-05	CEBPA, AR, CEBPB, APOE, PPARG, TP53, ADIPOR2, SMAD3, ADIPOR1, ADIPOQ
GO:0043566~structure-specific DNA binding	9.89E-05	RXRA, TP53, PCNA, SMAD3, MYC, CTNNB1
GO:0035257~nuclear hormone receptor binding	1.08E-04	CCNE1, RXRA, PPARG, RB1, CTNNB1
GO:0043565~sequence-specific DNA binding	1.12E-04	CEBPA, AR, CEBPB, RXRA, PPARG, ESR1, TP53, SMAD3, MYC, CTNNB1
GO:0019899~enzyme binding	2.31E-04	CCND1, CDKN2A, APOA1, CDKN2B, CCND2, TP53, SMAD3, RB1, CTNNB1
GO:0005179~hormone activity	3.97E-04	LEP, INS, IGF1, PRL, ADIPOQ
GO:0003707~steroid hormone receptor activity	5.23E-04	AR, RXRA, PPARG, ESR1
GO:0042562~hormone binding	5.23E-04	AR, ESR1, ADIPOR2, ADIPOR1
GO:0046982~protein heterodimerization activity	5.28E-04	CEBPB, APOE, PPARG, TP53, ADIPOR2, ADIPOR1
GO:0004879~ligand-dependent nuclear receptor activity	8.58E-04	AR, RXRA, PPARG, ESR1
GO:0042802~identical protein binding	8.89E-04	CEBPA, CEBPB, TNF, APOA1, APOE, ADIPOR2, SMAD3, ADIPOR1, ADIPOQ
GO:0005496~steroid binding	0.001143476	AR, APOA1, RXRA, ESR1
GO:0019901~protein kinase binding	0.00126415	CCND1, CDKN2A, CDKN2B, CCND2, SMAD3
GO:0004857~enzyme inhibitor activity	0.001699577	CDKN1C, CDKN1A, CDKN1B, CDKN2A, CDKN2B, ANGPTL4
GO:0050681~androgen receptor binding	0.003160934	CCNE1, RB1, CTNNB1
GO:0003677~DNA binding	0.004665383	CEBPA, AR, CEBPB, E2F4, RBL2, RXRA, PPARG, TP53, ESR1, SMAD3, RB1, CTNNB1, HES1, CDKN2A, PCNA, MYC
GO:0035258~steroid hormone receptor binding	0.006671667	CCNE1, RB1, CTNNB1
GO:0003704~specific RNA polymerase II transcription factor activity	0.008482733	CEBPA, RXRA, SMAD3
GO:0008289~lipid binding	0.014483883	AR, APOA1, APOE, RXRA, PPARG, ESR1

GO:0008083~growth factor activity	0.015087312	LEP, IL6, INS, IGF1
GO:0060228~phosphatidylcholine-sterol O-acyltransferase activator activity	0.016073144	APOA1, APOE
GO:0045294~alpha-catenin binding	0.016073144	NUMB, CTNNB1
GO:0016564~transcription repressor activity	0.01863935	CDKN1C, HES1, PPARG, SMAD3, RB1
GO:0042803~protein homodimerization activity	0.022338611	CEBPA, CEBPB, APOE, SMAD3, ADIPOQ
GO:0005138~interleukin-6 receptor binding	0.022431542	IL6, ADAM17
GO:0003713~transcription coactivator activity	0.031653703	CCNE1, RXRA, RB1, CTNNB1
GO:0005072~transforming growth factor beta receptor, cytoplasmic mediator activity	0.035028233	CDKN1B, SMAD3
GO:0005159~insulin-like growth factor receptor binding	0.038152564	INS, IGF1
GO:0017127~cholesterol transporter activity	0.038152564	APOA1, APOE
GO:0001540~beta-amyloid binding	0.041267018	APOA1, APOE
GO:0003702~RNA polymerase II transcription factor activity	0.043993834	CEBPA, CEBPB, RXRA, SMAD3
GO:0015248~sterol transporter activity	0.044371628	APOA1, APOE
GO:0070325~lipoprotein receptor binding	0.047466423	APOA1, APOE

Table S6. KEGG pathway enrichment of HTs.

Term	PValue	Genes
hsa04110:Cell cycle	4.68E-15	E2F4, RBL2, RBL1, TP53, SMAD3, RB1, CDKN1C, CCNE1, CCND1, CDKN1A, CDKN2A, CDKN1B, CDKN2B, CCND2, PCNA, MYC
hsa05200:Pathways in cancer	5.35E-11	CEBPA, IL6, AR, RXRA, PPARG, TP53, SMAD3, IGF1, RB1, CTNNB1, CCNE1, CCND1, CDKN1A, CDKN2A, CDKN1B, CDKN2B, ITGAV, MYC
hsa05215:Prostate cancer	1.41E-08	CCNE1, CDKN1A, CCND1, AR, CDKN1B, INS, TP53, IGF1, RB1, CTNNB1
hsa05222:Small cell lung cancer	1.61E-07	CCNE1, CCND1, CDKN1B, CDKN2B, ITGAV, RXRA, TP53, RB1, MYC
hsa04115:p53 signaling pathway	6.12E-07	CCNE1, CDKN1A, CCND1, CDKN2A, CCND2, TP53, IGF1, IGFBP3
hsa05220:Chronic myeloid leukemia	1.21E-06	CDKN1A, CCND1, CDKN1B, CDKN2A, TP53, SMAD3, RB1, MYC
hsa05216:Thyroid cancer	2.11E-06	CCND1, RXRA, PPARG, TP53, MYC, CTNNB1
hsa04920:Adipocytokine signaling pathway	9.67E-06	LEP, TNF, RXRA, ADIPOR2, ADIPOR1, ADIPOQ, CPT1A
hsa05219:Bladder cancer	1.41E-05	CDKN1A, CCND1, CDKN2A, TP53, RB1, MYC
hsa04350:TGF-beta signaling pathway	4.37E-05	TNF, E2F4, RBL2, CDKN2B, RBL1, SMAD3, MYC
hsa05214:Glioma	1.03E-04	CDKN1A, CCND1, CDKN2A, TP53, IGF1, RB1
hsa03320:PPAR signaling pathway	1.60E-04	APOA1, RXRA, PPARG, ADIPOQ, CPT1A, ANGPTL4
hsa05218:Melanoma	1.83E-04	CDKN1A, CCND1, CDKN2A, TP53, IGF1, RB1
hsa05223:Non-small cell lung cancer	7.10E-04	CCND1, CDKN2A, RXRA, TP53, RB1
hsa05212:Pancreatic cancer	0.002090518	CCND1, CDKN2A, TP53, SMAD3, RB1
hsa05210:Colorectal cancer	0.003670702	CCND1, TP53, SMAD3, MYC, CTNNB1
hsa04310:Wnt signaling pathway	0.005507714	CCND1, CCND2, TP53, SMAD3, MYC, CTNNB1
hsa04630:Jak-STAT signaling pathway	0.006149005	LEP, CCND1, IL6, CCND2, PRL, MYC
hsa05213:Endometrial cancer	0.007112706	CCND1, TP53, MYC, CTNNB1
hsa05410:Hypertrophic cardiomyopathy (HCM)	0.026731316	IL6, TNF, ITGAV, IGF1
hsa04930:Type II diabetes mellitus	0.049900633	TNF, INS, ADIPOQ
hsa04330:Notch signaling pathway	0.049900633	HES1, NUMB, ADAM17
